# Malonyl-CoA is a conserved endogenous ATP-competitive mTORC1 inhibitor

**DOI:** 10.1038/s41556-023-01198-6

**Published:** 2023-08-10

**Authors:** Raffaele Nicastro, Laura Brohée, Josephine Alba, Julian Nüchel, Gianluca Figlia, Stefanie Kipschull, Peter Gollwitzer, Jesus Romero-Pozuelo, Stephanie A. Fernandes, Andreas Lamprakis, Stefano Vanni, Aurelio A. Teleman, Claudio De Virgilio, Constantinos Demetriades

**Affiliations:** 1 Department of Biology, University of Fribourg, Fribourg, Switzerland; 2 Max Planck Institute for Biology of Ageing (MPI-AGE), Cologne, Germany; 3 German Cancer Research Center (DKFZ), Heidelberg, Germany; 4 Heidelberg University, Heidelberg, Germany; 5 University of Cologne, Cologne Excellence Cluster on Cellular Stress Responses in Aging-Associated Diseases (CECAD), Cologne, Germany; 6 Present Address: Unidad de Investigación Biomedica, Universidad Alfonso X El Sabio (UAX), Madrid, Spain

**Keywords:** TOR signalling, Nutrient signalling, Fatty acids

## Abstract

Cell growth is regulated by the mammalian/mechanistic target of rapamycin complex 1 (mTORC1), which functions both as a nutrient sensor and a master controller of virtually all biosynthetic pathways. This ensures that cells are metabolically active only when conditions are optimal for growth. Notably, although mTORC1 is known to regulate fatty acid biosynthesis, how and whether the cellular lipid biosynthetic capacity signals back to fine-tune mTORC1 activity remains poorly understood. Here we show that mTORC1 senses the capacity of a cell to synthesise fatty acids by detecting the levels of malonyl-CoA, an intermediate of this biosynthetic pathway. We find that, in both yeast and mammalian cells, this regulation is direct, with malonyl-CoA binding to the mTOR catalytic pocket and acting as a specific ATP-competitive inhibitor. When fatty acid synthase (FASN) is downregulated/inhibited, elevated malonyl-CoA levels are channelled to proximal mTOR molecules that form direct protein–protein interactions with acetyl-CoA carboxylase 1 (ACC1) and FASN. Our findings represent a conserved and unique homeostatic mechanism whereby impaired fatty acid biogenesis leads to reduced mTORC1 activity to coordinately link this metabolic pathway to the overall cellular biosynthetic output. Moreover, they reveal the existence of a physiological metabolite that directly inhibits the activity of a signalling kinase in mammalian cells by competing with ATP for binding.

## Abstract (web summary)

Nicastro, Brohée et al. find that the fatty acid synthesis intermediate malonyl-CoA inhibits mTORC1, showing mTORC1 senses the capacity of a cell to synthesise fatty acids and linking fatty acid generation with the overall biosynthetic output through mTORC1.

## Main

Cell growth is a high energy-consuming and hence, tightly regulated process. Cells accumulate mass by taking up essential nutrients from their environment and using them to build macromolecules, such as proteins, lipids and sugars^1^. The mammalian/mechanistic target of rapamycin complex 1 (mTORC1) is a central integration point in cellular signalling, linking metabolic cues to cell growth and homeostasis. Work over the last 15 years has identified complex signalling cascades through which growth factors, nutrients (like amino acids) and energy availability regulate mTORC1 (reviewed in refs. ^1–4^). Cholesterol levels were previously shown to influence mTORC1 activity at the lysosomal surface via mechanisms that involve the Niemann–Pick C1 (NPC1), SLC38A9 and Rag GTPase proteins^5–7^, with the latter also playing a central role in amino-acid and glucose sensing by recruiting mTOR at this subcellular location^8–10^. In turn, mTORC1 upregulates multiple metabolic processes, including protein and cholesterol biosynthesis^3,4^. Similarly, active mTORC1 promotes fatty acid (FA) biosynthesis by driving the expression of key enzymes in this process—like fatty acid synthase (FASN)—in response to growth factor signalling^11^. However, whether and how FA synthesis controls mTORC1 activity remains enigmatic.

**Fig. 1:**
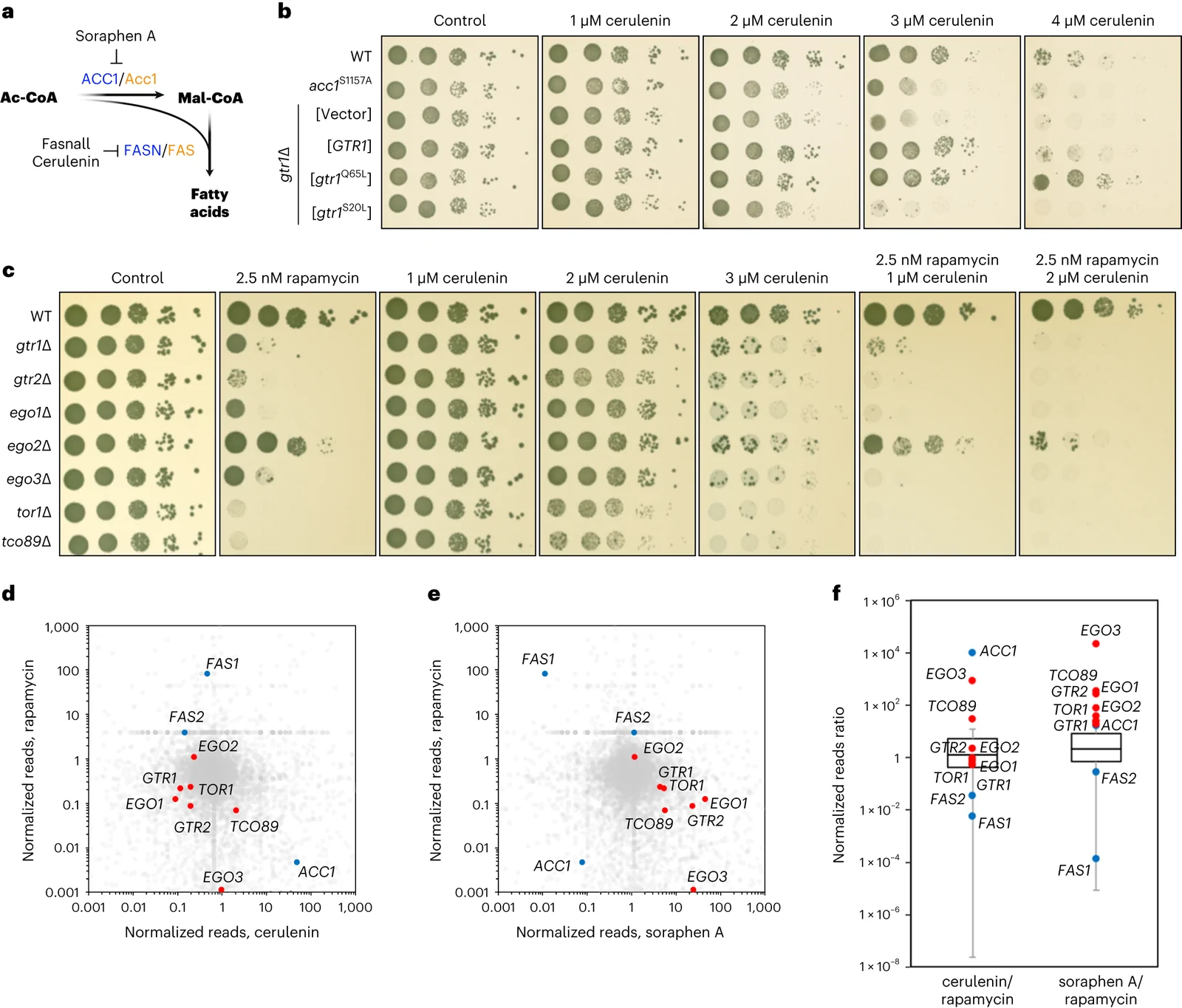
Functional pharmacogenetic interactions between TOR-pathway genes, and Acc1 and Fas1 activity in yeast. **a**, Schematic diagram of de novo FA biosynthesis. Yeast and mammalian proteins are shown in orange and blue, respectively. **b**, Yeast Rag GTPase mutants that impair or promote TORC1 activity are cerulenin-sensitive or cerulenin-resistant, respectively. Tenfold serial dilutions of wild-type (WT), hyperactive Acc1^S1157A^-expressing (*acc1*^*S1157A*^) and *gtr1Δ* cells expressing plasmid-encoded WT *GTR1*, *gtr1*^Q65L^ (TORC1-activating) or *gtr1*^S20L^ (TORC1-inactivating), or containing an empty vector were spotted on plates with the indicated concentrations of cerulenin or vehicle (control) and cultured at 30 °C for 2 d. **c**, TORC1 and EGOC mutants are sensitive to cerulenin and hypersensitive to combined cerulenin and rapamycin treatment. Drop spot assays were performed as in **b** using the indicated cell strains and plates containing the indicated concentrations of rapamycin and/or cerulenin. **d**, Positive correlation between rapamycin- and cerulenin-induced SATAY transposition profiles in TORC1 and EGOC genes. **e**, Negative correlation between rapamycin- and soraphen A-induced SATAY transposition profiles in TORC1 and EGOC genes. **d**,**e**, The dot plots show the ratio of transposition events (reads) per coding gene in rapamycin- and cerulenin-treated (**d**), and rapamycin- and soraphen A-treated (**e**) versus previously published untreated SATAY libraries^24,25^. **f**, Summary of the pairwise correlations of the transposon profiles of TORC1, EGOC (red dots) and FA biosynthesis (blue dots) genes shown in **d**,**e**. Boxplots: central line, median; box, interquartile range (IQR; 25th (Q1)–75th (Q3) percentile); and whiskers, Q3 + 1.5 × IQR and Q1 − 1.5 × IQR. Source numerical data are provided. Source data

Fatty acids serve both structural and regulatory roles in cells (for example, by participating in membrane formation and post-translationally modifying proteins, respectively), while also functioning as energy storage molecules^12,13^. In de novo FA biosynthesis, acetyl-CoA (generated from glucose catabolism) is converted to malonyl-CoA (Mal-CoA) by acetyl-CoA carboxylase 1 (ACC1)-mediated and ATP-dependent carboxylation, which is then used by FASN to produce palmitate, the precursor of longer FAs^14,15^. This process is well conserved from human to yeast cells, in which the orthologous acetyl-CoA carboxylase, Acc1, and the Fas1 and Fas2-containing FA synthase (FAS) complex catalyse the conversion of acetyl-CoA to FAs^16^ (Fig. 1a). Because various metabolites were previously shown to impact mTORC1 via regulation of the activity of upstream signalling components (for example, by AMP and inositol allosterically influencing AMPK activity^17,18^; or α-ketoglutarate and glutaminolysis somehow affecting the lysosomal Rag GTPases^19^), we hypothesised that a metabolic intermediate of FA biosynthesis may be signalling directly or indirectly to mTORC1, thus forming a regulatory feedback loop.

## Results

### Functional pharmacogenetic interactions between TOR-pathway components and the core FA biosynthesis machinery reveal a role for Mal-CoA in TOR signalling

**Fig. 2:**
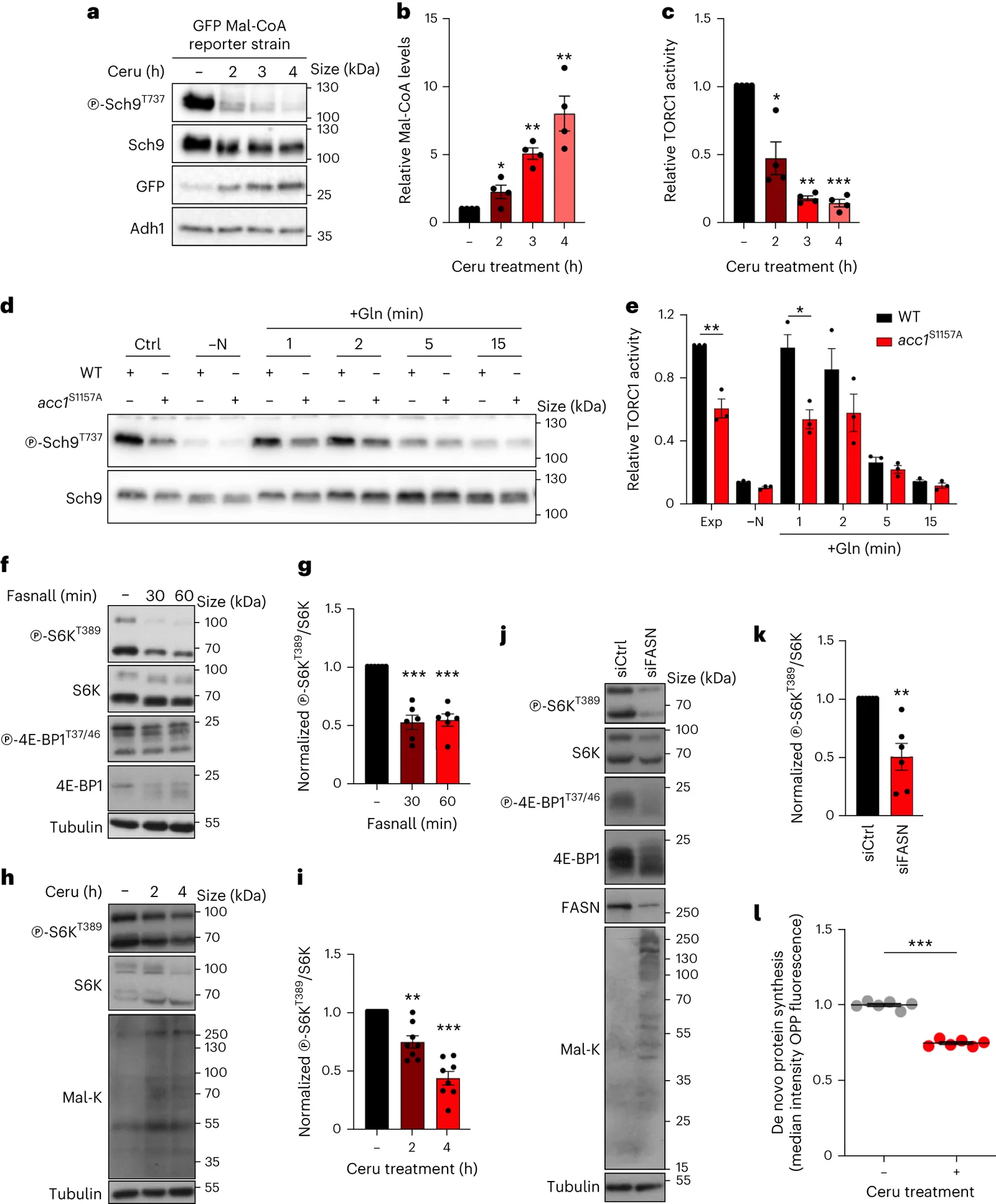
Mal-CoA level increase through genetic or pharmacological perturbation to Fas1 and Acc1 activity reduces mTORC1 activity in yeast and mammalian cells. **a**–**c**, Cerulenin boosts Mal-CoA levels and inhibits TORC1 activity (*n* = 4 independent experiments). **a**, Immunoblots of the lysates of yeast cells expressing the fapR/fapOp-yeGFP Mal-CoA reporter system treated with 20 μM cerulenin for the indicated times. GFP expression serves as an indicator of the Mal-CoA levels. Phosphorylation at T737 of Sch9 (p-Sch9^T737^) was used to assess TORC1 activity. **b**,**c**, Calculated relative Mal-CoA levels (GFP/Adh1 ratio; **b**) and relative TORC1 activity (p-Sch9^T737^/Sch9 ratio; **c**). **d**, Immunoblots of the lysates of wild-type (WT) and Acc1^S1157A^-expressing yeast cells that were cultured to the exponential phase (Ctrl), starved of nitrogen (–N), or starved and restimulated with Gln for the indicated times (*n* = 3 independent experiments). TORC1 activity was assessed by Sch9 phosphorylation. **e**, Levels of the relative TORC1 activity (p-Sch9^T737^/Sch9 ratio) in **d**. **f**,**g**, Pharmacological inhibition of FASN downregulates mTORC1. Phosphorylation of S6K at T389 and 4E-BP1 at T37 and T46 was used to assay mTORC1 activity (*n* = 6 independent experiments). **f**, Immunoblots of the lysates of HEK293FT cells treated with 25 μM Fasnall for the indicated times. **g**, Normalized p-S6K^T389^/S6K ratio. **h**,**i**, HEK293FT cells were treated with 50 μM cerulenin for the indicated times. The levels of p-S6K^T389^ were used to assay mTORC1 activity (*n* = 8 independent experiments). **h**, Mal-K immunoblots indicating total protein malonylation as a readout for Mal-CoA levels. **i**, Normalized p-S6K^T389^/S6K ratio. **j**,**k**, HEK293FT cells were treated with control siRNA (siCtrl) or siFASN (*FASN* knockdown). The levels of p-S6K^T389^ and p-4E-BP1^T37/46^ were used to assay mTORC1 activity (*n* = 6 independent experiments). **j**, Mal-K immunoblots showing total protein malonylation. **k**, Normalized p-S6K^T389^/S6K ratio. **l**, Effects of FASN inhibition on de novo protein synthesis. *O*-Propargyl-puromycin (OPP) incorporation assay for HEK293T cells treated with cerulenin (50 μM, 4 h) or dimethylsulfoxide (DMSO) as the control. Data represent the median fluorescence intensity of OPP Alexa Fluor 488; *n* = 6 biological replicates from two independent experiments. **b**,**c**,**e**,**g**,**i**,**k**,**l**, Data are the mean ± s.e.m. *P < 0.05, **P < 0.005, ***P < 0.0005. Ceru, cerulenin. Source numerical data and unprocessed blots are provided. Source data

**Extended Data Fig. 1:**
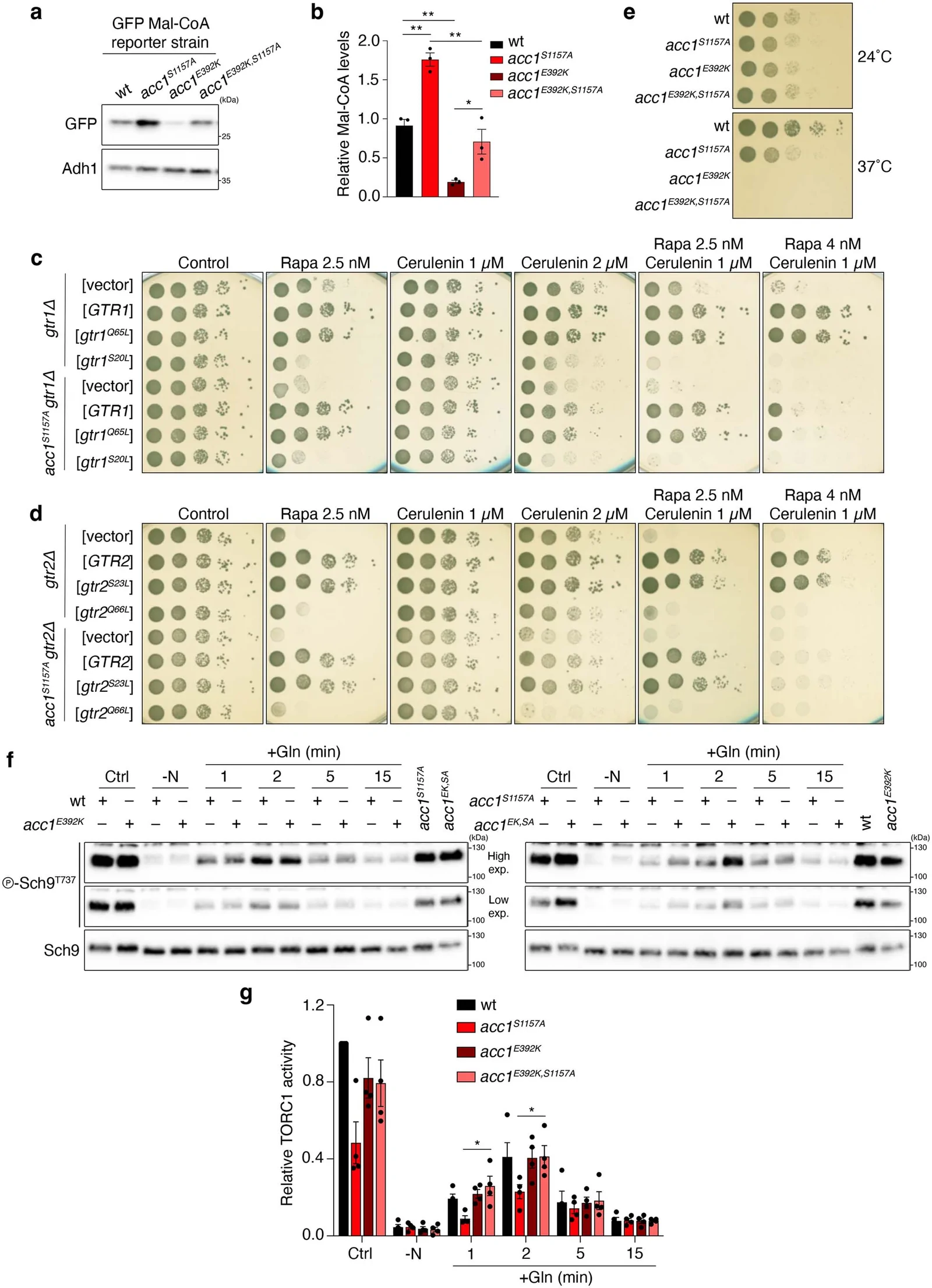
Perturbations to Acc1 influence Mal-CoA levels and TORC1 activity in yeast cells. **a**,**b**, Mal-CoA levels in WT and hyperactive *acc1*^S1157A^ cells are significantly reduced by introduction of the hypomorphic *acc1*^*E392K*^ mutation. Mal-CoA levels were assessed by GFP blots in cells grown at 24 °C (**a**) and quantified (**b**) as in Fig. 2a,b, respectively; *n* = 3 independent experiments. **c**,**d**, Pharmacogenetic interactions of rapamycin and cerulenin indicate a potential inhibitory role of Mal-CoA in TORC1 signalling. **c**, *gtr1Δ* and *acc1*^S1157A^*gtr1Δ* mutant strains containing an empty vector or expressing plasmid-encoded wild-type *GTR1*, *gtr1*^Q65L^ or *gtr1*^S20L^ were spotted and cultured on plates with the indicated concentrations of rapamycin and/or cerulenin. **d**, The respective *gtr2Δ* and *acc1*^S1157A^*gtr2Δ* mutant strains expressing plasmid-encoded wild-type *GTR2*, *gtr2*^S23L^ or *gtr2*^Q66L^ are shown; *n* = 3 independent experiments. **e**, The *acc1*^E392K^ mutation causes temperature-sensitive growth even when combined with the *acc1*^S1157A^ mutation. Exponentially growing cells with the indicated genotypes were spotted on plates (tenfold serial dilutions) and cultured for 3 d at 24 °C or 37 °C; *n* = 3 independent experiments. **f**,**g**, Mutation of E392K in *acc1*^S1157A^ suppresses the TORC1-inhibitory effect of *acc1*^S1157A^. Immunoblots (**f**) and quantifications of TORC1 activity (p-Sch9^Thr737^/Sch9; **g**) were carried out as in Fig. 2d,e, respectively. **f**, The samples derive from the same experiment and gels/blots were processed in parallel; *n* = 4 independent experiments. Data in graphs are shown as the mean ± s.e.m. *P < 0.05, **P < 0.005. Source numerical data and unprocessed blots are provided. Source data

Our previous interactome studies of the Rag GTPases in yeast indicated that the RagA and -B (RagA/B) orthologue Gtr1 may interact directly or indirectly with Acc1 and the Fas1 subunit of the FAS complex (Supplementary Table S1 in ref. ^20^). Because both these enzymes are part of essential protein complexes that work together to synthesise FAs, this suggested a possible functional interplay between TORC1 signalling and the core FA biosynthetic machinery. To study this, we first probed the pharmacogenetic interaction of TORC1-pathway mutants with the FAS inhibitor cerulenin^21,22^. Interestingly, manipulations that reduce TORC1 activity—such as loss of Gtr1 and -2 (Gtr1/2), expression of nucleotide-free Gtr1^S20L^ or loss of other TORC1-pathway components (that is, Ego1–3, Tor1 and Tco89)^23^—rendered cells highly sensitive to cerulenin and indicated an additive effect of low doses of rapamycin and cerulenin (Fig. 1b,c). Conversely, expression of the GTP-locked Gtr1^Q65L^ allele, which activates TORC1, caused cerulenin resistance (Fig. 1b). These observations match our targeted re-analyses of previously published saturated transposon analyses in yeast (SATAY) data^24,25^ showing that transposon events in TORC1-pathway genes were under-represented when cells were cultured on cerulenin or rapamycin (Fig. 1d,f). However, in these datasets, TORC1-pathway genes were over-represented when cells were cultured in the presence of soraphen A, which inhibits Acc1, the antecedent enzyme in FA biosynthesis^26^ (Fig. 1e,f). One interpretation of these findings is that Mal-CoA, the intermediate metabolite between Acc1 and FAS, may be functionally linked to TORC1 activity. In support of this hypothesis, cerulenin-mediated FAS inhibition, which increases intracellular Mal-CoA levels more than eightfold (as assayed with a specific green fluorescent protein (GFP)-based reporter system^27^; Fig. 2a,b), strongly reduced TORC1 activity (assayed by the phosphorylation of its direct target Sch9) in vivo (Fig. 2a,c). Next, we hypothesised that FAS inhibition could be downregulating TORC1 either by increasing the levels of its substrate (that is, Mal-CoA) or reducing the levels of its product (that is, palmitate). To distinguish between these two possibilities, we used the hyperactive Acc1^S1157A^ allele, which causes Mal-CoA levels to increase (Extended Data Fig. 1a,b)^27–29^. This allele aggravated the sensitivity of cells to sublethal combinations of cerulenin and rapamycin (Extended Data Fig. 1c,d), indicating that the elevated levels of Mal-CoA—and not the reduced palmitate levels—are responsible for this phenotype. Together, these data suggest that TORC1 may be inhibited by Mal-CoA in response to perturbations to Acc1 and FAS activity.

**Extended Data Fig. 2:**
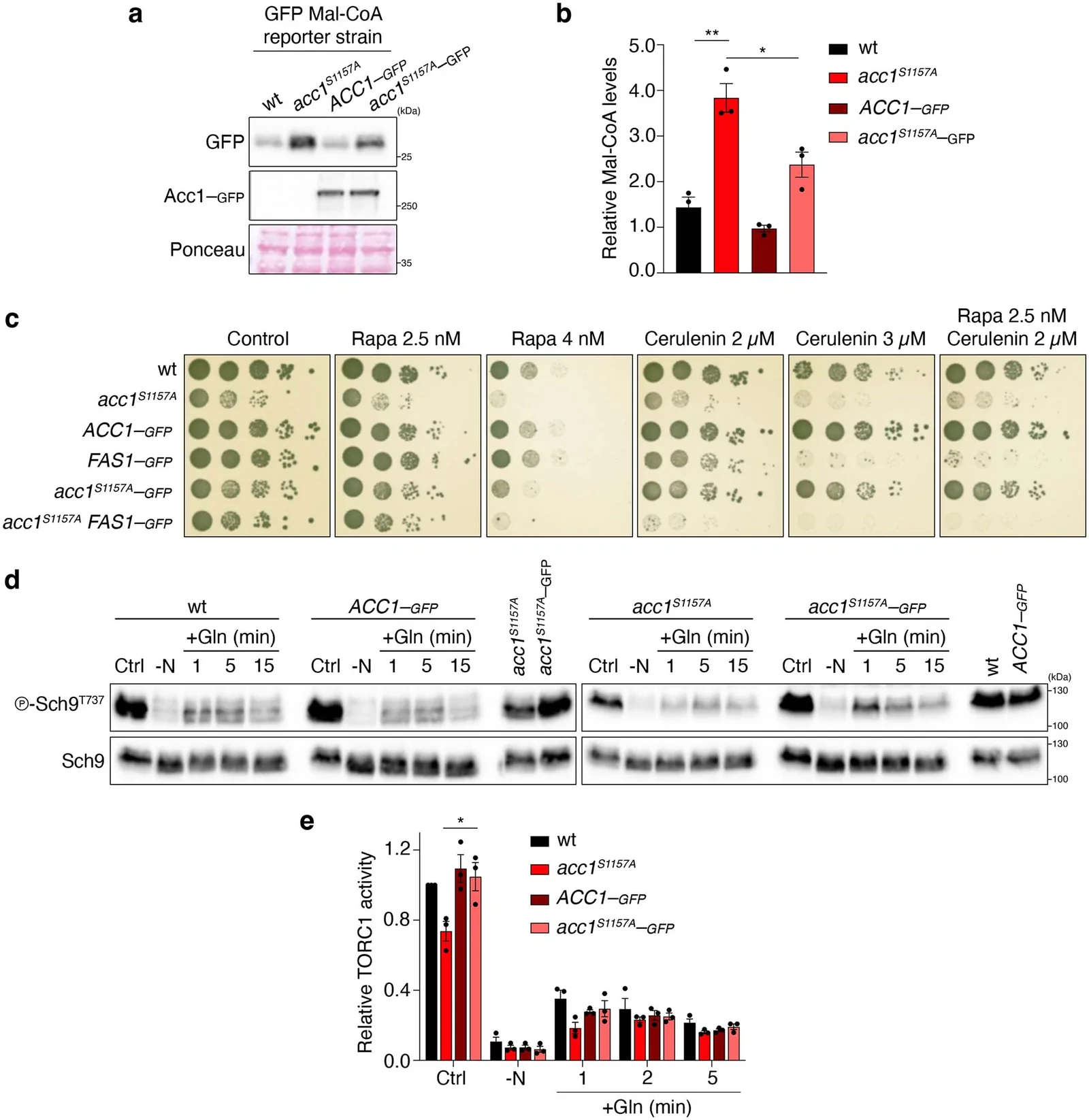
Carboxy-terminal tagging of Acc1^S1157A^ restrains its capacity to stimulate Mal-CoA levels and inhibit TORC1 in yeast. **a**,**b**, Carboxy-terminal GFP tagging of endogenous Acc1^S1157A^ significantly reduces Mal-CoA levels. See Fig. 2a,b for details. **a**, Ponceau staining as loading control. **b**, Quantification of relative Mal-CoA levels (GFP/Ponceau signal); *n* = 3 independent experiments. **c**, Carboxy-terminal GFP tagging of endogenous Acc1^S1157A^ partially reverts its ability to render cells rapamycin- and cerulenin-sensitive. In control experiments, C-terminal GFP tagging of endogenous Fas1 rendered WT cells cerulenin-sensitive and further enhanced the cerulenin sensitivity of *acc1*^S1157A^ cells, while marginally affecting the rapamycin-sensitivity of the respective cells. Spot assays with the indicated yeast genotypes and rapamycin and/or cerulenin concentrations performed as in Fig. 1b; *n* = 3 independent experiments. **d**,**e**, Carboxy-terminal GFP tagging of endogenous Acc1^S1157A^ suppresses its ability to inhibit TORC1 in exponentially growing and Gln-restimulated cells. **d**, TORC1 activities assessed as in Fig. 2d. **e**, Quantification of relative TORC1 activity (p-Sch9^Thr737^/Sch9). **d**, The samples derive from the same experiment and gels/blots were processed in parallel; *n* = 3 independent experiments. Data in graphs are shown as the mean ± s.e.m. *P < 0.05, **P < 0.005. Source numerical data and unprocessed blots are provided. Source data

Providing support for this model, *acc1*^S1157A^ cells cultured to the exponential phase exhibited significantly reduced basal TORC1 activity and partially impaired glutamine-stimulated reactivation of TORC1, following nitrogen starvation (Fig. 2d,e). These defects correlated well with significantly increased levels of Mal-CoA in cells expressing Acc1^S1157A^ (Extended Data Fig. 1a,b). Moreover, when the *acc1*^S1157A^ mutation was combined with the *acc1*^E392K^ mutation (also known as *acc1-7-1*; ref. ^30^)—a temperature-sensitive allele (Extended Data Fig. 1e) that is hypomorphic for Mal-CoA production at the permissive temperature (Extended Data Fig. 1a,b)—both the elevated Mal-CoA levels and the reduced TORC1 activity observed in *acc1*^S1157A^ cells were suppressed (Extended Data Fig. 1a,b,f,g). Similarly, carboxy (C)-terminal GFP tagging of Acc1^S1157A^ rendered this allele less active, which significantly reduced the cellular levels of Mal-CoA (Extended Data Fig. 2a,b) and suppressed the sensitivity to cerulenin and rapamycin (Extended Data Fig. 2c) as well as the TORC1 activity defect (Extended Data Fig. 2d,e) that are observed in untagged Acc1^S1157A^-expressing cells. In contrast, C-terminal GFP tagging of Fas1 led to cerulenin sensitivity in wild-type cells and further enhanced this effect in *acc1*^S1157A^ cells (Extended Data Fig. 2c).

### FASN inhibition controls mTORC1 activity independently of FA biosynthesis

**Fig. 3:**
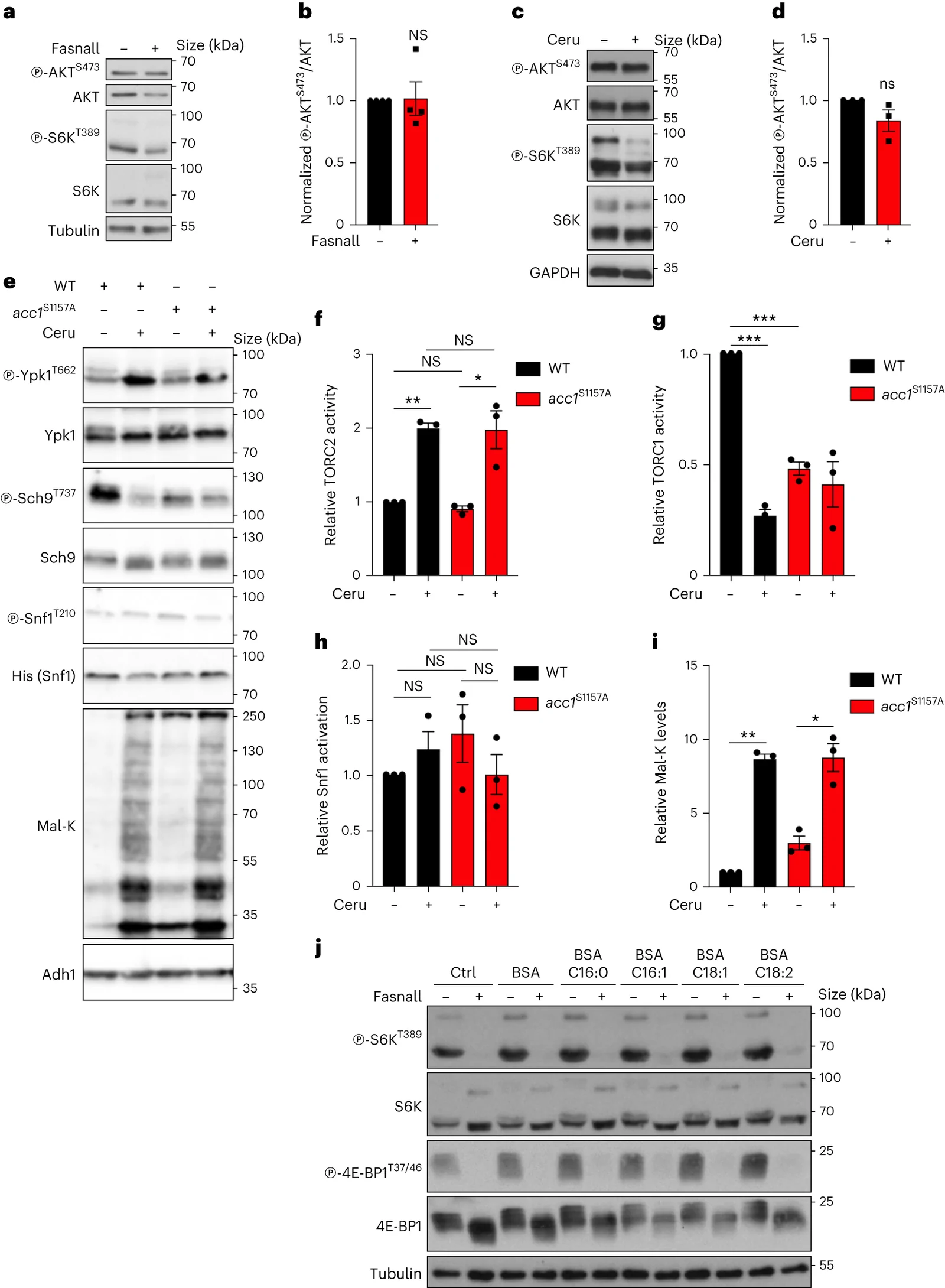
Perturbations to the activity of FASN/Fas1 or yeast Acc1 specifically downregulate mTORC1, but not mTORC2, independently of FA availability. **a**,**b**, Fasnall treatment does not influence mTORC2 activity in HEK293FT cells. Cells were treated with 25 μM Fasnall or DMSO (–) as the control for 30 min (*n* = 4 independent experiments). **a**, Immunoblotting for AKT phosphorylation at S473 was used to assess mTORC2 activity. **b**, Levels of mTORC2 activity (p-AKT^S473^/AKT). **c**,**d**, Effect of cerulenin treatment (50 μM, 4 h) on cells as in **a**,**b** (*n* = 3 independent experiments). **d**, Levels of mTORC2 activity (p-AKT^S473^/AKT). **e**–**i**, Neither expression of the *acc1*^S1157A^ allele nor treatment of yeast cells with cerulenin (20 μM, 2 h) downregulates TORC2 or Snf1 (yeast AMPK) activity. **e**, Lysates from control (−) or cerulenin-treated wild-type (WT) and *acc1*^S1157A^ mutant cells were immunoblotted with the indicated antibodies (*n* = 3 independent experiments). Phosphorylation of Ypk1 was used as the TORC2 readout. Phosphorylation of Snf1 was used as the AMPK activation readout. Total Snf1 was detected with an antibody to a histidine (His) stretch in Snf1. Mal-K blots showing total protein malonylation, which is indicative of intracellular Mal-CoA levels. **f**–**i**, Levels of TORC2 activity (p-Ypk1^T662^/Ypk1; **f**), TORC1 activity (p-Sch9^T737^/Sch9; **g**), Snf1 activation (p-Snf1^T210^/His; **h**) and lysine malonylation (Mal-K/Adh1; **i**). **j**, Inhibition of FASN downregulates mTORC1 activity independently of lipid availability. Immunoblots with lysates from control (−) or Fasnall-treated (25 μM, 30 min) HEK293FT cells supplemented with BSA-conjugated FAs, as indicated, or BSA as a control. Phosphorylation of S6K and 4E-BP1 was used to assay mTORC1 activity (*n* = 2 independent experiments). **b**,**d**,**f**,**g**,**h**,**i**, Data are the mean ± s.e.m. *P < 0.05; **P < 0.005; ***P < 0.0005; and NS, not significant. Ctrl, control; and Ceru, cerulenin. Source numerical data and unprocessed blots are provided. Source data

**Extended Data Fig. 3:**
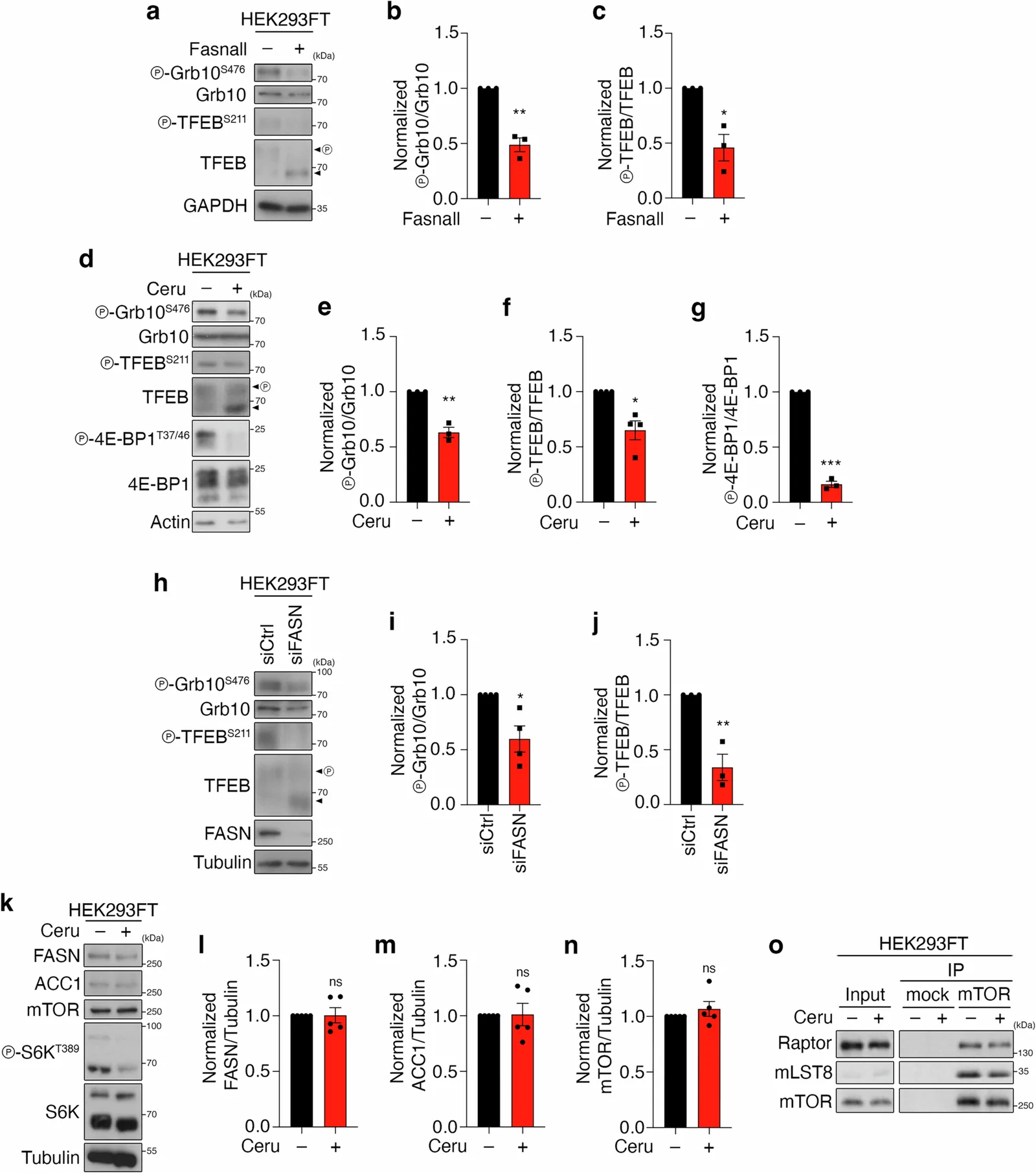
FASN inhibition/knockdown decreases mTORC1 activity towards multiple substrates, without affecting FASN/ACC1/mTOR levels or mTORC1 stability. **a**–**c**, Fasnall downregulates mTORC1 activity towards Grb10 and TFEB. Cells were treated for 30 min with 25 μM Fasnall or DMSO (−) as the control. **b**, Quantification of Grb10 phosphorylation (p-Grb10^T389^/Grb10). **c**, Quantification of TFEB phosphorylation (p-TFEB^S211^/TFEB); *n* = 3 independent experiments. **d**–**g**, Cerulenin downregulates mTORC1 activity towards Grb10, TFEB and 4E-BP1. **d**, Cells were treated for 4 h with 50 μM cerulenin or DMSO (−) as the control. **e**, Quantification of Grb10 phosphorylation (p-Grb10^T389^/Grb10); *n* = 3 independent experiments. **f**, Quantification of TFEB phosphorylation (p-TFEB^S211^/TFEB); *n* = 4 independent experiments. **g**, Quantification of 4E-BP1 phosphorylation (p-4E-BP1^T37/46^/4E-BP1); *n* = 3 independent experiments. **h**–**j**, As in **a**–**c**, but with transient *FASN* knockdown. *n*_Grb10_ = 4 and *n*_TFEB_ = 3 independent experiments. **k**–**n**, FASN, ACC1 and mTOR total protein levels are not affected by short-term FASN inhibition (4 h cerulenin). **k**, Immunoblots for the indicated proteins. **l**–**n**, Quantification of relative FASN (**l**), ACC1 (**m**) and mTOR levels (**n**); *n* = 5 independent experiments. **o**, Stability and composition of mTORC1 is not affected by FASN inhibition with cerulenin (4 h); *n* = 2 independent experiments. Data in graphs are shown as the mean ± s.e.m. *P < 0.05, **P < 0.005, ***P < 0.0005. Source numerical data and unprocessed blots are provided. Source data

**Extended Data Fig. 4:**
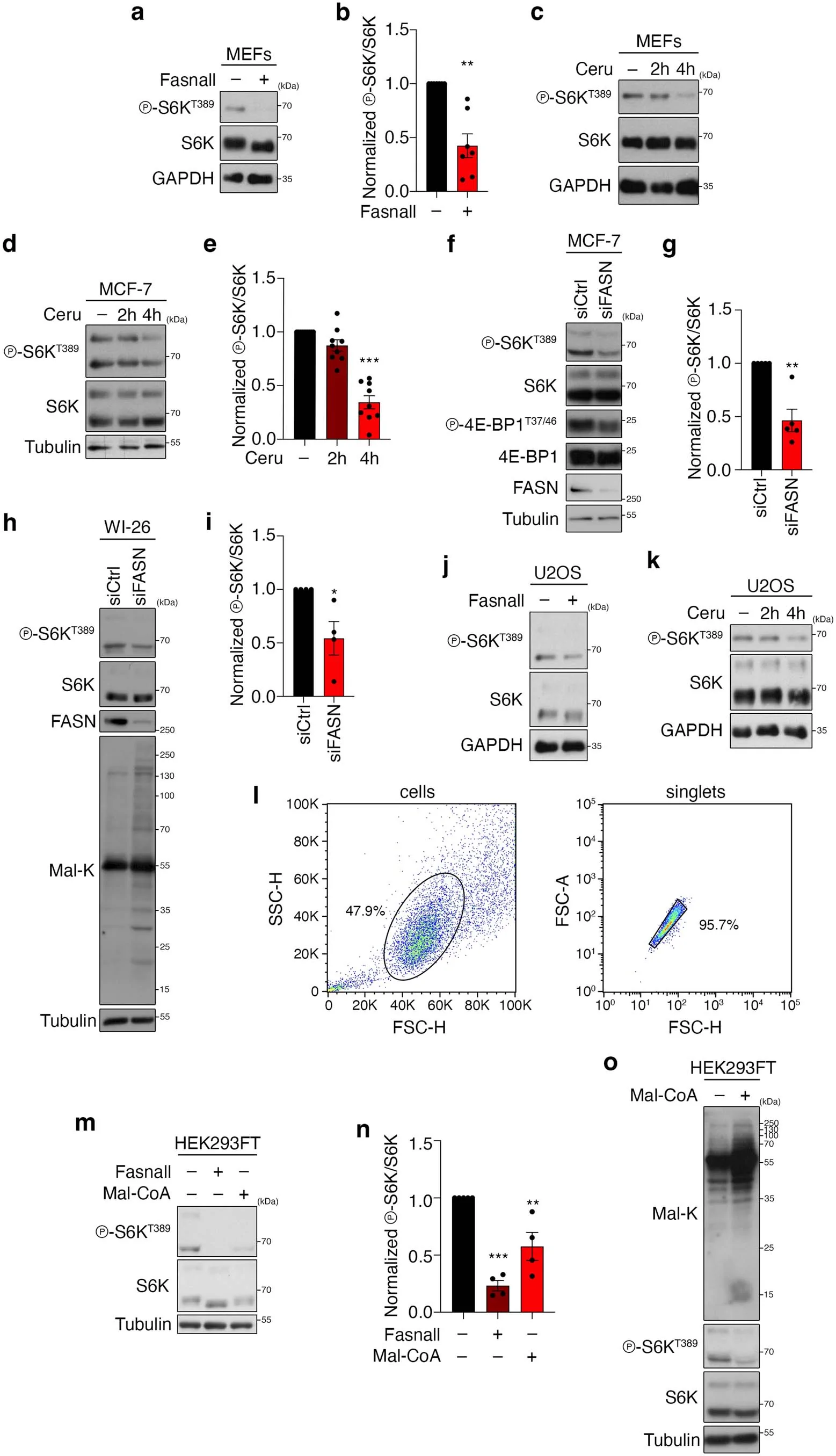
FASN blockage or exogenous Mal-CoA downregulates mTORC1 in different mammalian cell lines. **a**,**b**, Fasnall downregulates mTORC1 activity in mouse embryonic fibroblasts (MEFs.) **a**, Cells were treated for 30 min with 25 μM Fasnall or DMSO (−) as the control. **b**, Quantification of mTORC1 activity (p-S6K^T389^/S6K); *n* = 7 independent experiments. **c**, Cerulenin downregulates mTORC1 activity in MEFs. Cells treated for 2 or 4 h with 50 μM Cerulenin (Ceru) or DMSO (4 h) as the control (−); *n* = 3 independent experiments. **d**,**e**, Cerulenin downregulates mTORC1 activity in MCF-7. **d**, Cells were treated for 2 or 4 h with 50 μM cerulenin (Ceru) or DMSO (4 h) as the control (−). **e**, Quantification of mTORC1 activity (p-S6K^T389^/S6K); *n* = 9 independent experiments. **f**,**g**, *FASN* knockdown downregulates mTORC1 activity in MCF-7. **g**, Quantification of mTORC1 activity (p-S6K^T389^/S6K);. *n* = 5 independent experiments. **h**,**i**, *FASN* knockdown downregulates mTORC1 activity and increases Mal-CoA levels in WI-26 fibroblasts. **h**, Mal-K blots show total protein malonylation, indicative of intracellular Mal-CoA levels. **i**, Quantification of mTORC1 activity (p-S6K^T389^/S6K);. *n* = 4 independent experiments. **j**, Fasnall downregulates mTORC1 activity in U2OS. Cells treated for 30 min with 25 μM Fasnall or DMSO as control (−); *n* = 3 independent experiments. **k**, Cerulenin downregulates mTORC1 activity in U2OS. Cells were treated for 2 or 4 h with 50 μM cerulenin (Ceru) or DMSO (4 h) as the control (−); *n* = 2 independent experiments. **l**, Gating strategy for the flow-cytometry-based OPP incorporation assay shown in Fig. 2l. **m**–**o**, Exogenous Mal-CoA addition is capable of downregulating mTORC1. **m**, HEK293FT cells were treated with 25 μM Fasnall or 250 μM Mal-CoA for 30 min. **n**, Quantification of mTORC1 activity (p-S6K^T389^/S6K). **o**, Total protein malonylation (Mal-K blots) indicates uptake of exogenously supplemented Mal-CoA ; *n* = 4 independent experiments. Data in graphs are shown as the mean ± s.e.m. *P < 0.05, **P < 0.005, ***P < 0.0005. Source numerical data and unprocessed blots are provided. Source data

**Extended Data Fig. 5:**
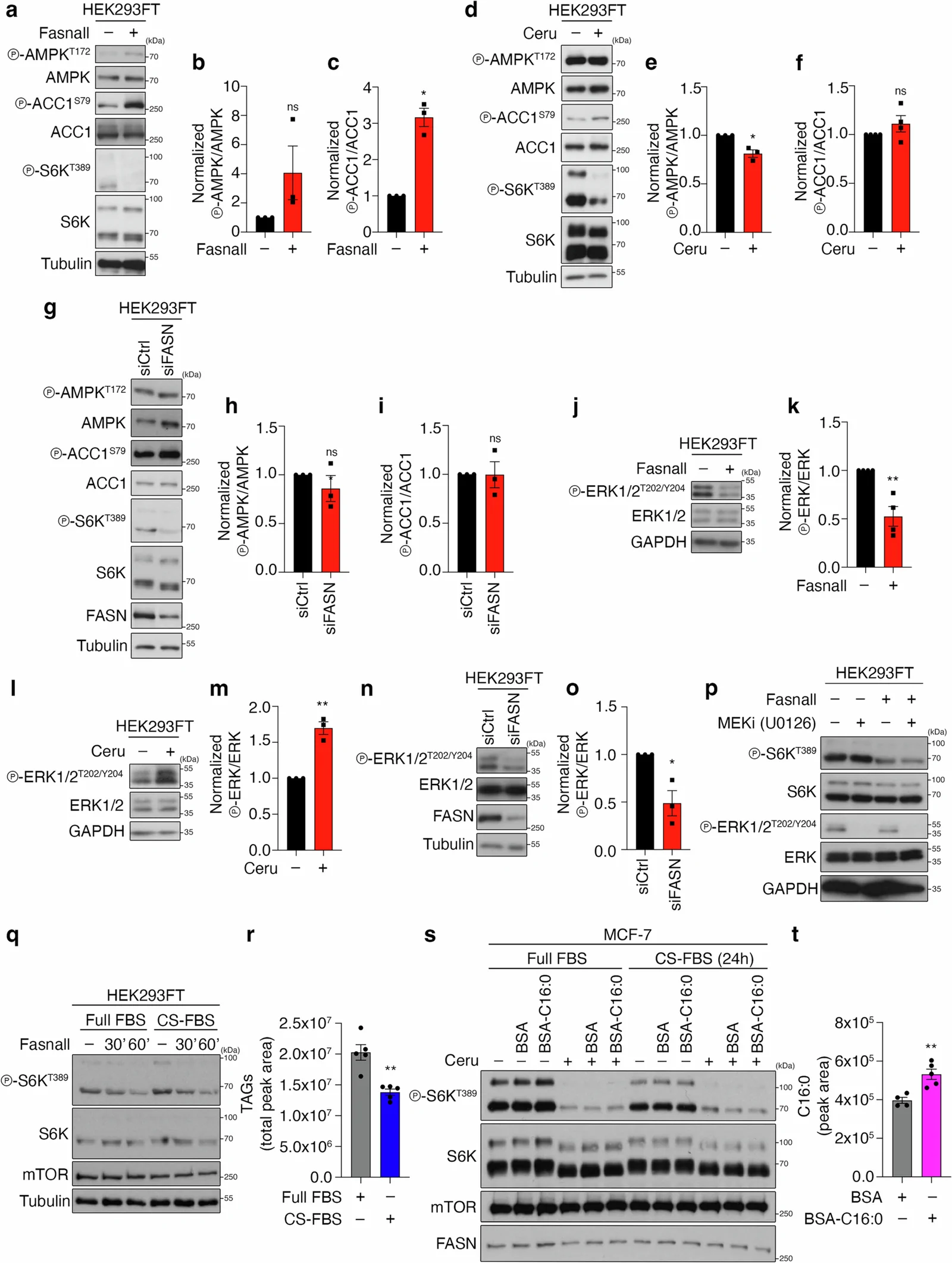
FASN inhibition/knockdown downregulates mTORC1 activity independently from changes in AMPK or ERK signalling and from lipid availability. **a**–**c**, AMPK and ACC1 phosphorylation in cells treated with Fasnall. **b**, Quantification of AMPK phosphorylation; *n* = 3 independent experiments. **c**, Quantification of ACC1 phosphorylation; *n* = 3 independent experiments. **d**–**f**, As in **a**–**c**, but for cerulenin treatment; *n*_p-AMPK_ = 3 independent experiments and *n*_p-ACC1_ = 4 independent experiments. **g**–**i**, As in **a**–**c**, but for *FASN* knockdown; *n* = 3 independent experiments. **j**,**k**, ERK1/2 phosphorylation in cells treated with Fasnall. **k**, Quantification of ERK phosphorylation; *n* = 4 independent experiments. **l**,**m**, As in **j**,**k**, but for cerulenin treatment; *n* = 3 independent experiments. **n**,**o**, As in **j**,**k**, but for *FASN* knockdown; *n* = 4 independent experiments. **p**, Pharmacological inhibition of MEK–ERK signalling does not influence the effect of FASN inhibition on mTORC1; *n* = 2 independent experiments. **q**,**r**, Depletion of exogenous lipid sources does not influence the mTORC1 response to FASN inhibition. HEK293FT cells were cultured in full FBS- or charcoal-stripped FBS (CS-FBS)-containing media for 24 h, and then treated with 25 μM Fasnall for 30 or 60 min, or DMSO as the control (–). **q**, mTORC1 activity was assessed by phosphorylation of S6K. **r**, Measurement of intracellular TAG levels; *n* = 5 biological replicates. **s**,**t**, Immunoblots with lysates from control (−) or cerulenin-treated (50 μM, 4 h) MCF-7 cells, supplemented with BSA-conjugated palmitate (C16:0) or BSA as control. Cells were cultured in full FBS- or charcoal-stripped FBS (CS-FBS)-containing media for 24 h before the treatments. **s**, mTORC1 activity was assayed by phosphorylation of S6K. **t**, Measurement of intracellular C16:0 levels, indicating uptake of BSA-conjugated palmitate; *n*_BSA_ = 4 and *n*_C16:0_ = 5 biological replicates. Data in graphs are shown as the mean ± s.e.m. *P < 0.05, **P < 0.005. Source numerical data and unprocessed blots are provided. Source data

A similar molecular machinery is responsible for FA biosynthesis in mammalian cells, which differ from yeast by expressing a single FA synthase (FASN) enzyme that catalyses the conversion of Mal-CoA to palmitate and other FAs (Fig. 1a). Therefore, we sought to investigate whether accumulation of Mal-CoA also influences mTORC1 activity in mammalian cells. To test this, we blocked FASN activity in human HEK293FT cells either by specific pharmacological inhibition using Fasnall (also known as benzenesulfonate^31^; Figs. 1a and 2f,g) or cerulenin (Figs. 1a and 2h,i), or by transient *FASN* knockdown by transfection with small-interfering-RNAs (siRNAs) targeting *FASN* (siFASN; Fig. 2j,k). In accordance with the yeast data, all perturbations suppressed mTORC1 activity, as indicated by decreased phosphorylation of its direct substrates S6K, 4E-BP1, Grb10 and TFEB (Fig. 2f–k and Extended Data Fig. 3a–j) without FASN inhibition affecting the total protein levels of FASN, ACC1 and mTOR (Extended Data Fig. 3k–n) or mTORC1 integrity (Extended Data Fig. 3o). As expected, both cerulenin treatment and *FASN* knockdown led to a detectable increase in total protein malonylation—assessed by immunoblotting with an antibody to malonyl-lysine (Mal-K; Fig. 2h,j)—which is indicative of elevated intracellular Mal-CoA levels^32,33^. Similar results were obtained by inhibiting or knocking down FASN in mouse embryonic fibroblasts (MEFs; Extended Data Fig. 4a–c), MCF-7 human breast cancer cells (Extended Data Fig. 4d–g), WI-26 human lung fibroblasts (Extended Data Fig. 4h,i) and U2OS human osteosarcoma cells (Extended Data Fig. 4j,k), showing that this effect is not cell-type- or species-specific. Consistent with the well-established role of mTORC1 as a regulator of mRNA translation, via the phosphorylation of its downstream substrates (like S6K and 4E-BP1), cerulenin treatment also led to decreased de novo protein synthesis (Fig. 2l and Extended Data Fig. 4l). Further supporting that Mal-CoA is able to inhibit mTORC1, exogenous addition of this metabolite^34,35^ caused a significant drop in mTORC1 activity by elevating the levels of intracellular Mal-CoA (Extended Data Fig. 4m–o). Importantly, FASN inhibition specifically affected mTORC1, but not mTORC2, as treatment with Fasnall or cerulenin did not significantly alter the phosphorylation of Akt, a typical mTORC2 substrate in human cells (Fig. 3a–d). Similarly, treatment with cerulenin or Acc1^S1157A^ expression did not inhibit the phosphorylation of Ypk1^T662^, a bona fide TORC2 target residue in yeast (Fig. 3e–i). The same was true for the activity of other key kinases that are known to act upstream of mTORC1, like AMPK and ERK, which was not consistently affected by perturbations to the activity or levels of FASN (Extended Data Fig. 5a–o). Accordingly, pharmacological inhibition of MEK–ERK signalling did not influence the effect that FASN inhibition had on mTORC1 (Extended Data Fig. 5p), further indicating that it acts independently of upstream signalling.

**Fig. 4:**
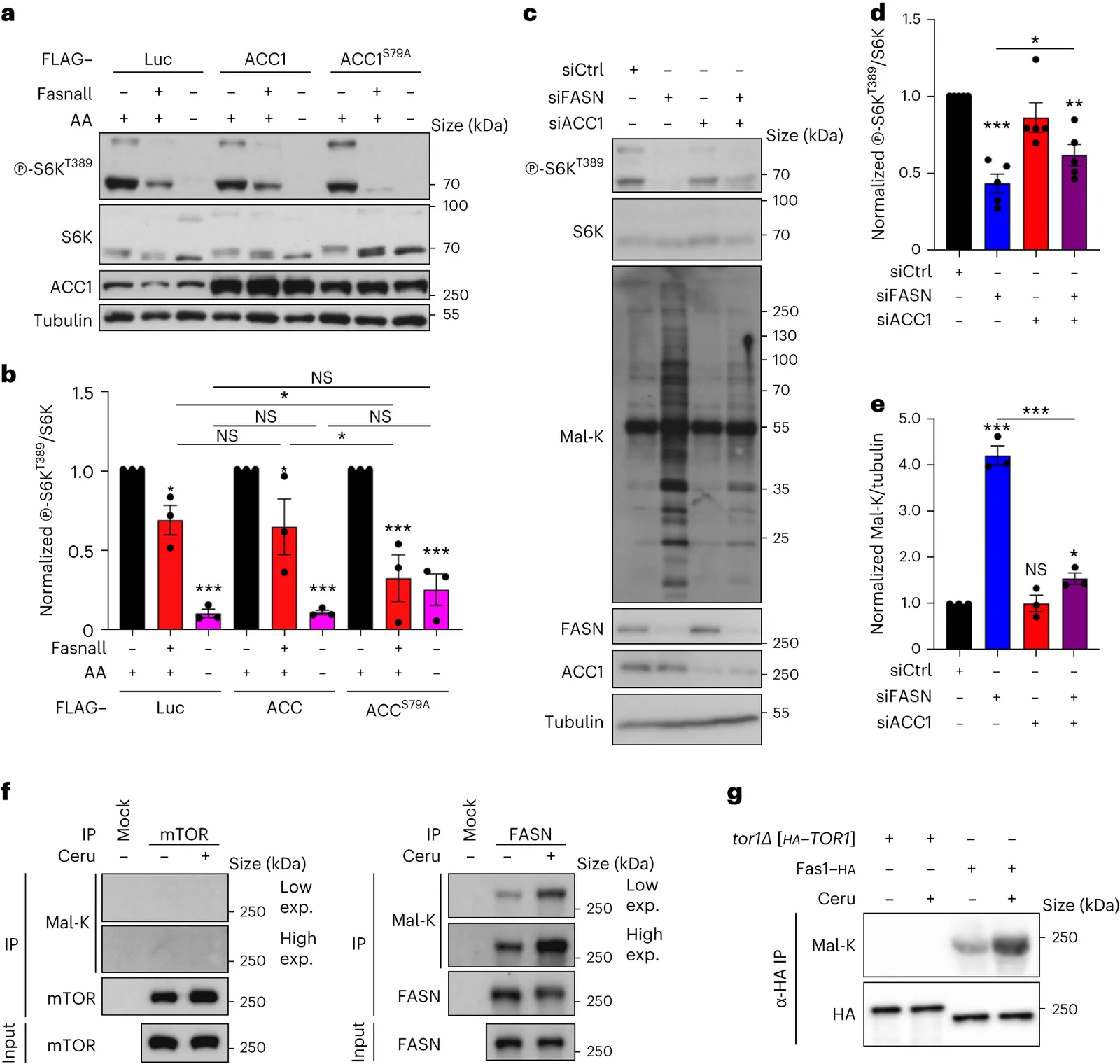
Mal-CoA accumulation following perturbations to ACC1 and FASN inhibits mTORC1 independently of mTOR malonylation. **a**,**b**, Exogenous expression of a hyperactive ACC1 mutant (ACC1^S79A^) cooperates with FASN inhibition to downregulate mTORC1 in HEK293FT cells without influencing the response to amino-acid (AA) starvation. The cells were transfected with vectors expressing FLAG-tagged WT ACC1, ACC1^S79A^ or Luciferase (Luc; as the control) and treated with Fasnall (25 μM, 30 min) or AA-starvation medium (1 h) as indicated (*n* = 3 independent experiments). **a**, Phosphorylation of S6K at T389 was used to assay mTORC1 activity. **b**, Levels of mTORC1 activity (p-S6K^T389^/S6K ratio). **c**–**e**, ACC1 knockdown partially restores the increase in Mal-CoA levels and rescues the downregulation of mTORC1 caused by silencing of *FASN.* siCtrl, control siRNA; and siACC1, siRNA to *ACC1*. **c**, Immunoblots of HEK293T cell lysates. **d**, Calculated levels of mTORC1 activity (p-S6K^T389^/S6K ratio; *n* = 5 independent experiments). **e**, Levels of Mal-K (Mal-K/tubulin ratio; *n* = 3 independent experiments). **f**, Lack of detectable mTOR malonylation in HEK293FT cells. Endogenous mTOR (left) and FASN (right) proteins were immunoprecipitated from control (−) or cerulenin-treated (50 μM, 4 h) cells. **g**, Tor1 is not malonylated in yeast cells. Amino (N)-terminally HA-tagged Tor1 or C-terminally HA-tagged Fas1 was immunoprecipitated from control (−) or cerulenin-treated (10 μM, 2 h) cells cultured to the exponential phase; α-HA, anti-HA. **f**,**g**, Protein malonylation was assessed using anti-Mal-K (*n* = 3 independent experiments); IP, immunoprecipitate; and exp., exposure. **b**,**d**,**e**, Data are the mean ± s.e.m. *P < 0.05; **P < 0.005; ***P < 0.0005; and NS, not significant. Ceru, cerulenin. Source numerical data and unprocessed blots are provided. Source data

**Extended Data Fig. 6:**
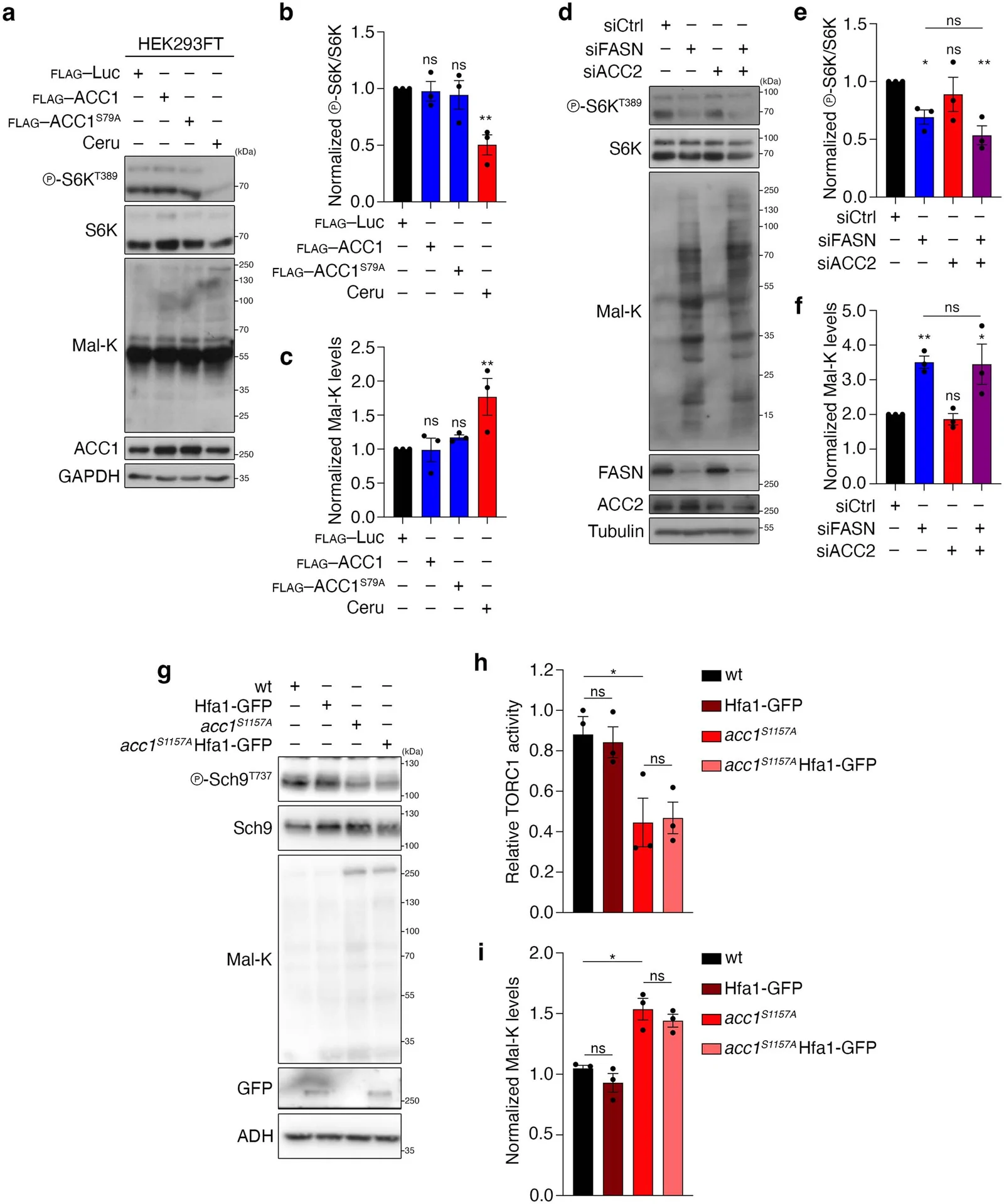
Unlike ACC1/Acc1, ACC2/Hfa1 is not involved in the regulation of mTORC1/TORC1 by Mal-CoA. **a**–**c**, Unlike FASN inhibition by treatment with cerulenin, exogenous overexpression of WT or a hyperactive ACC1 mutant (ACC1^S79A^) is not able to significantly alter Mal-CoA levels and mTORC1 activity in mammalian cells. **b**,**c**, Quantification of S6K phosphorylation (**b**) and Mal-K levels (**c**); *n* = 3 independent experiments. **d**–**f**, Unlike ACC1 (see Fig. 4c–e), ACC2 knockdown does not restore the increase in Mal-CoA levels or rescue the downregulation of mTORC1 caused by silencing of FASN. **e**, Quantification of mTORC1 activity (p-S6KT389/S6K). **f**, Quantification of Mal-K levels (Mal-K/loading controls); *n* = 3 independent experiments. **g**–**i**, C-terminal GFP tagging of the yeast ACC2 orthologue, Hfa1, does not affect TORC1 activity or cellular Mal-CoA levels. **h**,**i**, Quantification of Sch9 phosphorylation (**h**) and Mal-K levels (**i**); *n* = 3 independent experiments. Data in graphs are shown as the mean ± s.e.m. *P < 0.05, **P < 0.005. Source numerical data and unprocessed blots are provided. Source data

Palmitate, the main FASN product, was previously suggested to be important for mTORC1 activity in specialized cell types^36–38^. We therefore investigated whether FASN inhibition downregulates mTORC1 due to a decrease in palmitate or an increase in Mal-CoA by modulating the levels of these two metabolites. Notably, neither culturing cells in charcoal-stripped fetal bovine serum (FBS)—which is depleted of lipids and causes intracellular triacylglycerol (TAG) levels to decrease (Extended Data Fig. 5q,r)—nor supplementing the culture medium with bovine serum albumin (BSA)-conjugated FAs (Fig. 3j and Extended Data Fig. 5s,t) modulated the inhibition of mTORC1 by Fasnall or cerulenin treatment. Consistent with Mal-CoA being the key metabolite that inhibits mTORC1 when it accumulates, exogenous expression of the constitutively active ACC1^S79A^ mutant demonstrated a synergistic effect with Fasnall towards mTORC1 inhibition without significantly affecting the response to amino-acid starvation (Fig. 4a,b). Notably, unlike for FASN inhibition, overexpression of wild-type ACC1 or ACC1^S79A^ alone was not sufficient to significantly elevate the levels of endogenous Mal-CoA or downregulate mTORC1 activity (Fig. 4a,b and Extended Data Fig. 6a–c), suggesting that active FASN is still capable of processing the additional Mal-CoA produced by active ACC1 in mammalian cells. Furthermore, although *ACC1* knockdown alone did not influence mTORC1 activity or the levels of intracellular Mal-CoA, it did partially restore the siFASN-induced increase in Mal-K levels and prevented the decrease in S6K phosphorylation (Fig. 4c–e). In contrast, knockdown of *ACC2*, the paralogous enzyme that localizes to the outer mitochondrial membrane, did not influence the levels of Mal-K or mTORC1 activity in control or *FASN-*knockdown cells, indicating that it does not contribute to the observed phenotype (Extended Data Fig. 6d–f). The same was true in yeast, in which C-terminal GFP tagging of the yeast ACC2 orthologue Hfa1 (which compromised Acc1 function; Extended Data Fig. 2) did not affect TORC1 activity or Mal-CoA levels (Extended Data Fig. 6g–i).

A previous study proposed that malonylation of mTOR on K1218 following prolonged FASN inhibition or knockdown negatively impacts mTORC1 activity in endothelial cells^39^. We therefore investigated whether the accumulation of Mal-CoA following FASN/Fas1 inhibition is downregulating mTORC1/TORC1 via this mechanism in our system. However, using an antibody that detects malonylated lysine (Mal-K) residues on proteins, we were unable to detect malonylation of immunopurified human mTOR or yeast Tor1 from control or cerulenin-treated cells (Fig. 4f,g). In contrast to mTOR, and in agreement with previous proteomic analyses of the human malonylome^33,40^, lysine malonylation was readily and robustly detectable on immunopurified human FASN and yeast Fas1 from control cells, with the malonylation increasing further in cerulenin-treated cells (Fig. 4f,g). Together, our data from yeast and mammalian cells indicate that hyperactivation of Acc1 or inhibition of FASN/Fas1 downregulate mTORC1/TORC1 via a mechanism that involves the accumulation of Mal-CoA, independently of FA biosynthesis and mTOR malonylation. Moreover, they indicate that, in mammalian cells, the rate-limiting step in this process is FASN activity and/or levels, with the activation status of ACC1 playing a secondary role.

### Perturbations to yeast and mammalian ACC1/FAS activity control mTORC1 independently of upstream regulators

**Fig. 5:**
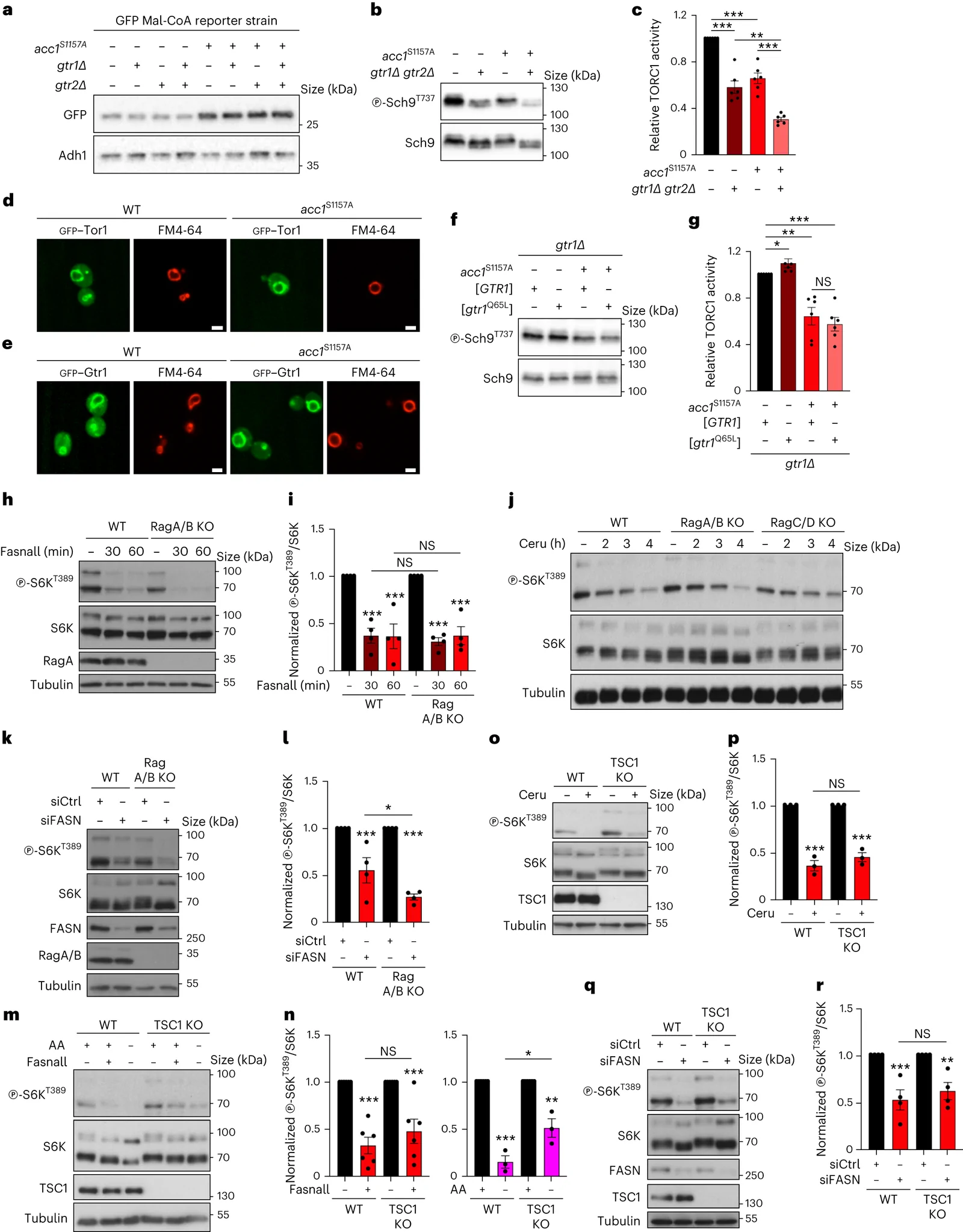
Perturbations to Acc1 and FASN/Fas1 downregulate mTORC1 independently of key upstream regulators. **a**, Immunoblots with lysates from the indicated yeast strains analysed as in Fig. 2a (*n* = 3 independent experiments). **b**, Immunoblots of the lysates of the indicated yeast strains assayed for TORC1 activity as in Fig. 2a. **c**, The level of phosphorylation at T737 of Sch9 (p-Sch9^T737^/Sch9) was used to assay the TORC1 activity levels in **b** (*n* = 6 independent experiments). **d**,**e**, Constitutive activation of Acc1 (*acc1*^S1157A^) does not alter vacuolar morphology or the subcellular localization of GFP–Tor1 (**d**) or GFP-Gtr1 (**e**). Vacuoles were stained with FM4-64. Scale bars, 5 μm; *n* = 3 independent experiments. **f**,**g**, Expression of the GTP-locked Gtr1^Q65L^ allele does not suppress the TORC1 inhibition mediated by the Acc1^S1157A^ allele. **g**, Levels of TORC1 activity (p-Sch9^T737^/Sch9; *n* = 6 independent experiments). **h**,**i**, FASN inhibition downregulates mTORC1 activity independently of the Rags. **h**, *RagA*/*B*-knockout and WT HEK293FT were treated with 25 μM Fasnall for the indicated times and their lysates were immunoblotted. **i**, Levels of mTORC1 activity (p-S6K^T389^/S6K) normalized to the respective DMSO-treated control (*n* = 4 independent experiments). **j**, *RagA*/*B*-knockout, *RagC*/*D*-knockout and WT HEK293FT cells were treated with 50 μM cerulenin for the indicated times (*n* = 4 independent experiments). **k**,**l**, *RagA*/*B*-knockout and WT HEK293FT were treated as in **h** but with *FASN* knockdown. Levels of mTORC1 activity (p-S6K^T389^/S6K) normalized to the respective DMSO-treated control (*n* = 4 independent experiments). **m**,**n**, FASN inhibition downregulates mTORC1 activity independently of the TSC complex. **m**, *TSC1*-knockout and WT HEK293FT cells were treated with 25 μM Fasnall (30 min) or amino-acid-starvation medium (AA; 1 h). **n**, Levels of mTORC1 activity (p-S6K^T389^/S6K) normalized to the respective DMSO-treated control (*n*_Fasnall_ = 6 (left) and *n*_AA_ = 3 independent experiments (right)). **o**,**p**, *TSC1*-knockout and WT HEK293FT cells were treated with cerulenin (50 μM, 4 h) as in **m**,**n**. **p**, Levels of mTORC1 activity (p-S6K^T389^/S6K) normalized to the respective DMSO-treated control (*n* = 3 independent experiments). **q**,**r**, *TSC1*-knockout and WT HEK293FT cells were treated as in **m**,**n** but with *FASN* knockdown. Levels of mTORC1 activity (p-S6K^T389^/S6K) normalized to the respective control knockdown (*n* = 4 independent experiments). **c**,**g**,**i**,**l**,**n**,**p**,**r**, Data are the mean ± s.e.m. *P < 0.05; **P < 0.005; ***P < 0.0005; and NS, not significant. Ceru, cerulenin; and KO, knockout. Source numerical data and unprocessed blots are provided. Source data

To study how Mal-CoA may be regulating TORC1 activity, we first employed genetic epistasis in yeast. Nutrients such as amino acids regulate TORC1 in part via the Gtr/Rag GTPases^23^. However, expression of constitutively active Acc1^S1157A^ rendered not only wild-type cells but also cells lacking Gtr1 and Gtr2 expression (*gtr1Δ* and *gtr2Δ*, respectively) or with *gtr1* and *gtr2* mutations (*gtr1*^S20L^ and *gtr2*^Q66L^, respectively) cerulenin-sensitive (Extended Data Fig. 1c,d), indicating that Acc1 or its product Mal-CoA may impinge on TORC1 independently of Gtr1/2. To further corroborate this assumption, we assayed the effects of hyperactive Acc1^S1157A^ expression on TORC1 following the combined loss of Gtr1/2, which in control experiments did not prevent the Acc1^S1157A^-mediated increase in Mal-CoA (Fig. 5a). As expected, both the expression of Acc1^S1157A^ and loss of Gtr1/2 reduced TORC1 activity (Fig. 5b,c). Expression of Acc1^S1157A^ in the Gtr1/2-double-mutant background decreased TORC1 activity further, suggesting that Acc1^S1157A^ acts on TORC1 independently of Gtr1/2 (Fig. 5b,c). Furthermore, the presence of the Acc1^S1157A^ allele did not affect the vacuolar localization of GFP-tagged Tor1 or Gtr1 (Fig. 5d,e), whereas expression of constitutively active Gtr1^Q65L^ was unable to revert the Acc1^S1157A^-mediated TORC1 inhibition (Fig. 5f,g). Together, these results establish that the effects of uncontrolled, Acc1-dependent Mal-CoA synthesis on TORC1 do not require the presence of the Rag GTPases in yeast cells.

**Extended Data Fig. 7:**
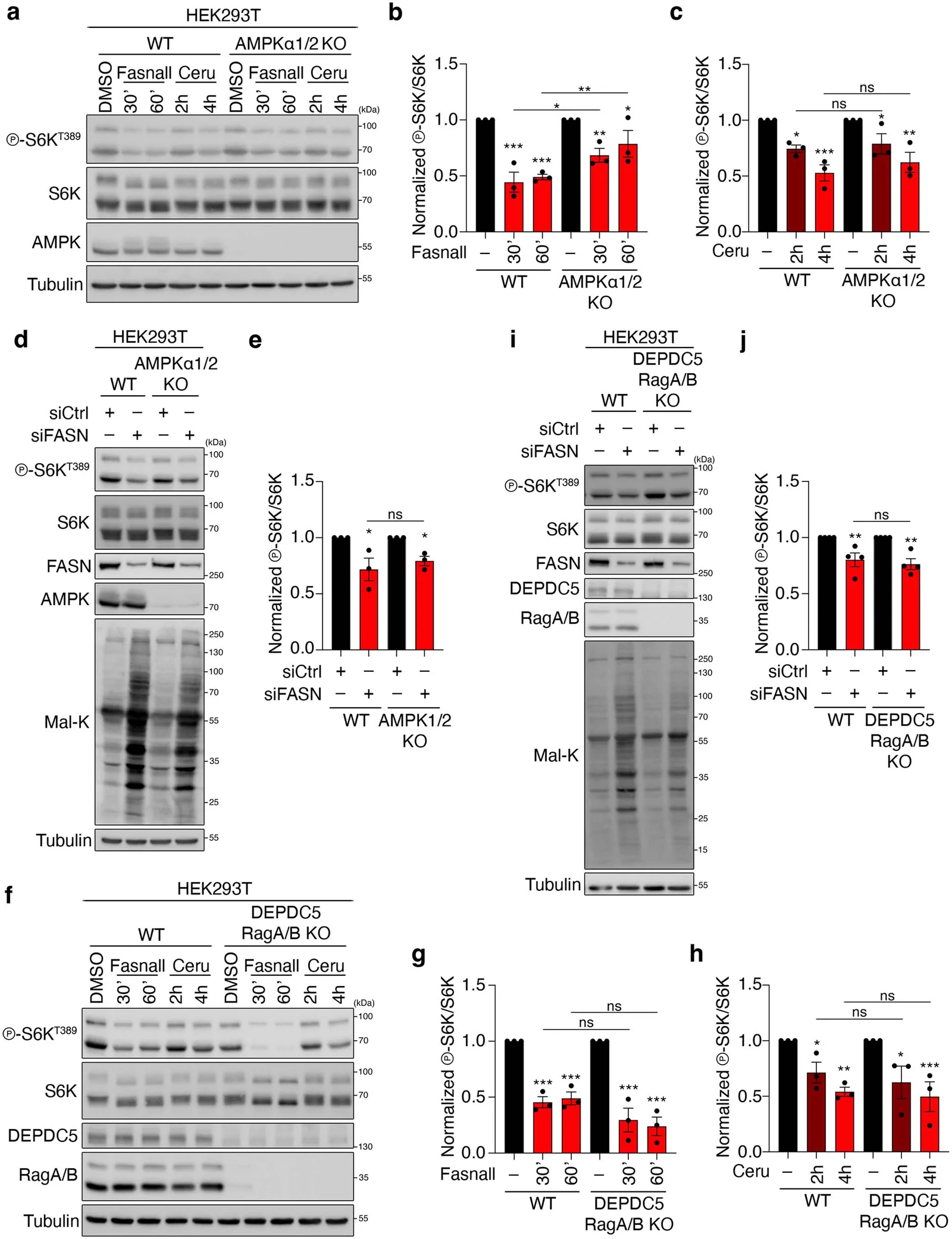
Blockage of FASN affects mTORC1 activity independently of AMPK and the GATOR1 complex. **a**–**c**, WT or AMPKα1/2 KO HEK293T cells were treated with 25 μM Fasnall (30, 60 min) or 50 μM Cerulenin (2, 4 h). **b**,**c**, Quantification of mTORC1 activity (p-S6K^T389^/S6K) for Fasnall (**b**), normalized to the respective DMSO-treated control, and cerulenin (**c**) treatment; *n* = 3 independent experiments. **d**,**e**, As in **a**, but for *FASN* knockdown. **e**, Quantification of mTORC1 activity (p-S6K^T389^/S6K), normalized to each siCtrl sample; *n* = 3 independent experiments. **f**–**h**, As in **a**–**c**, but in *DEPDC5*/*RagA*/*RagC*-triple-knockout cells. **g**,**h**, Quantification of mTORC1 activity (p-S6K^T389^/S6K) for Fasnall (**g**), normalized to the respective DMSO-treated control, and cerulenin (**h**) treatment; *n* = 3 independent experiments. **i**,**j**, As in **f**–**h**, but for *FASN* knockdown. **j**, Quantification of mTORC1 activity (p-S6K^T389^/S6K), normalized to the respective siCtrl sample; *n* = 4 independent experiments. Data in graphs are shown as the mean ± s.e.m. *P < 0.05, **P < 0.005, ***P < 0.0005. Source numerical data and unprocessed blots are provided. Source data

The heterodimeric Rag GTPases and the tuberous sclerosis complex (TSC) are two major signalling hubs upstream of mTORC1 in mammalian cells^8,9,41–44^. Upstream of the Rags lies the pentameric GATOR1 protein complex, which signals amino-acid sufficiency to the Rag dimer^45,46^. In parallel, the AMPKα1 and -2 (AMPKα1/2) kinases regulate mTORC1 in response to energetic stress through multiple mechanisms that involve the phosphorylation of TSC2 (ref. ^47^) or interactions within the lysosomal amino-acid-sensing machinery^48^. Therefore, we next investigated whether Mal-CoA regulates mTORC1 through one of these upstream complexes. In agreement with our yeast data, perturbation of FASN by Fasnall, cerulenin or siFASN decreased mTORC1 activity to a similar extent in wild-type as well as *RagA*/*B*- and *RagC/D*-double-knockout cells, indicating that it acts independently of the Rags (Fig. 5h–l). Furthermore, *TSC1*-knockout cells, which demonstrate a compromised response to amino-acid removal^42^, showed a similar mTORC1 downregulation compared with wild-type controls when treated with Fasnall (Fig. 5m,n), cerulenin (Fig. 5o,p) or siFASN (Fig. 5q,r). Similar data were obtained from *AMPKα1*/*2*-double-knockout as well as *DEPDC5* and *RagA/B*-triple-knockout cells (which have disrupted GATOR1 complex and Rag dimer activities; Extended Data Fig. 7a–j). Because inhibition of FASN can inactivate mTORC1 independently of all the major upstream signalling hubs tested, this suggests that Mal-CoA accumulation acts downstream of these regulatory complexes, possibly by acting directly on mTORC1.

**Extended Data Fig. 8:**
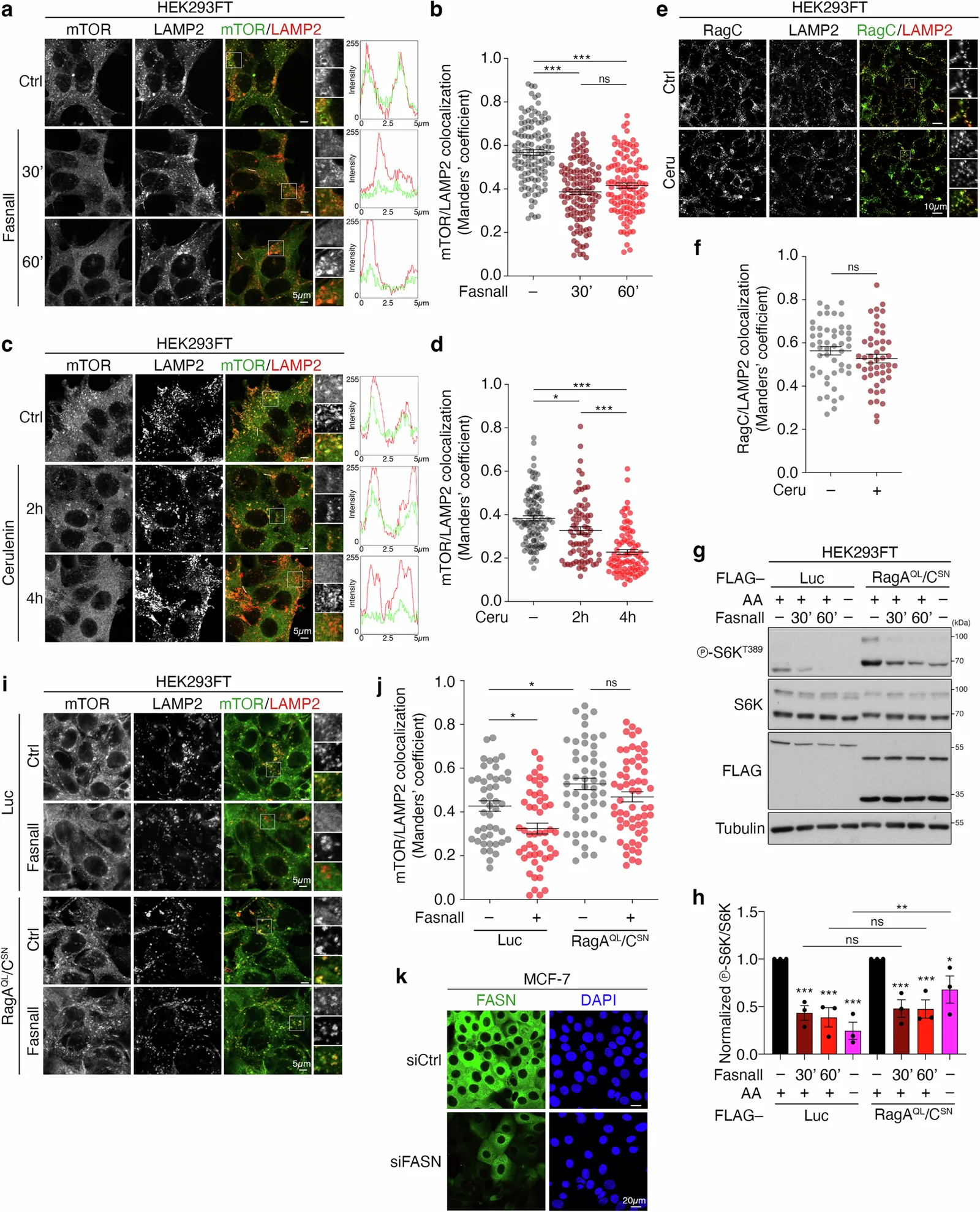
Downregulation of mTORC1 activity following FASN inhibition occurs independently of its lysosomal localization. **a**, Co-localization analysis, using confocal microscopy, of mTOR with LAMP2 (lysosomal marker) in HEK293FT cells treated with 25 μM Fasnall for the indicated times. Magnified insets and intensity plots for selected regions shown on the right. Scale bars, 5 μm. **b**, Quantification of co-localization from *n*_DMSO_ = 124, *n*_Fasnall_30’_ = 130 and *n*_Fasnall_60’_ = 117 individual cells. Pooled data from four independent experiments. **c**,**d**, As in **a**,**b**, but inhibiting FASN with 50 μM cerulenin for the indicated times. **c**, Scale bars, 5 μm. **d**, Quantification from *n*_DMSO_ = 103, *n*_Ceru_2h_ = 74 and *n*_Ceru_4h_ = 82 individual cells. Pooled data from three independent experiments. **e**,**f**, RagC stays lysosomal following FASN inhibition. As in **c**,**d**, but for RagC/LAMP2 co-localization. Scale bars, 10 μm. **f**, Quantification of co-localization from *n*_DMSO_ = 49 and *n*_Ceru_4h_ = 48 individual cells. Data from a representative experiment of two independent replicates are shown. **g**,**h**, Constitutively active Rags that blunt the amino-acid-starvation response do not prevent mTORC1 downregulation by FASN inhibition. **g**, HEK293FT cells were transiently transfected with vectors expressing FLAG-tagged RagA^QL^ and RagC^SN^ mutants, or Luciferase (Luc) as the control and treated with 25 μM Fasnall for the indicated times, or amino-acid starvation media for 1 h. **h**, Quantification of mTORC1 activity (p-S6K^T389^/S6K), normalized to each DMSO-treated control; *n* = 3 independent experiments. **i**,**j**, Co-localization analysis of mTOR with LAMP2 in HEK293FT cells transiently expressing FLAG-tagged RagA^QL^ and RagC^SN^ mutants, or Luciferase (Luc) as control, and treated with 25 μM Fasnall. **i**, Scale bars, 5 μm. **j**, Quantification of co-localization from *n*_Luc_DMSO_ = 47, *n*_Luc_Fasnall_ = 50, *n*_Rag_DMSO_ = 52 and *n*_Rag_Fasnall_ = 60 cells. Data from a representative experiment of two independent replicates are shown. **k**, Specific staining of endogenous FASN shown by knockdown (siFASN) and immunofluorescence. Nuclei were stained with DAPI. Scale bars, 20 μm; *n* = 3 independent experiments. Data in graphs are shown as the mean ± s.e.m. *P < 0.05, **P < 0.005, ***P < 0.0005. Source numerical data and unprocessed blots are provided. Source data

The lysosomal recruitment of mTORC1 by the Rag GTPases is an important aspect of its reactivation in response to amino-acid re-supplementation^8,41^. Here we observed that FASN inhibition by Fasnall or cerulenin led to a significant dissociation of mTOR from lysosomes, as assayed by its co-localization with the lysosomal marker LAMP2 (Extended Data Fig. 8a–d). In contrast, the lysosomal localization of RagC, which is also indirectly tethered to lysosomes through interactions with the pentameric LAMTOR complex, was unaffected by FASN inhibition, indicating that the relocalization of mTOR is not due to a general effect on lysosomal membrane proteins (Extended Data Fig. 8e,f). To investigate whether the lysosomal delocalization of mTORC1 is the underlying cause of its downregulation following FASN inhibition, we exogenously expressed an ‘active’-locked RagA/C mutant dimer (RagA^QL^/C^SN^) that partially prevents mTORC1 inactivation following amino-acid starvation^8,41^ (Extended Data Fig. 8g,h). Although expression of ‘active’-locked Rags rescued the lysosomal localization of mTOR in Fasnall-treated cells (Extended Data Fig. 8i,j), FASN inhibition was equally capable of downregulating mTORC1 in cells that exogenously express RagA^QL^/C^SN^ or an unrelated control protein (Extended Data Fig. 8g,h). These data show that the delocalization of mTOR away from lysosomes following FASN inhibition is not the cause of its inactivation.

### The mTOR–FASN–ACC1 proteins form reciprocal interactions in yeast and mammalian cells at multiple subcellular locations

**Fig. 6:**
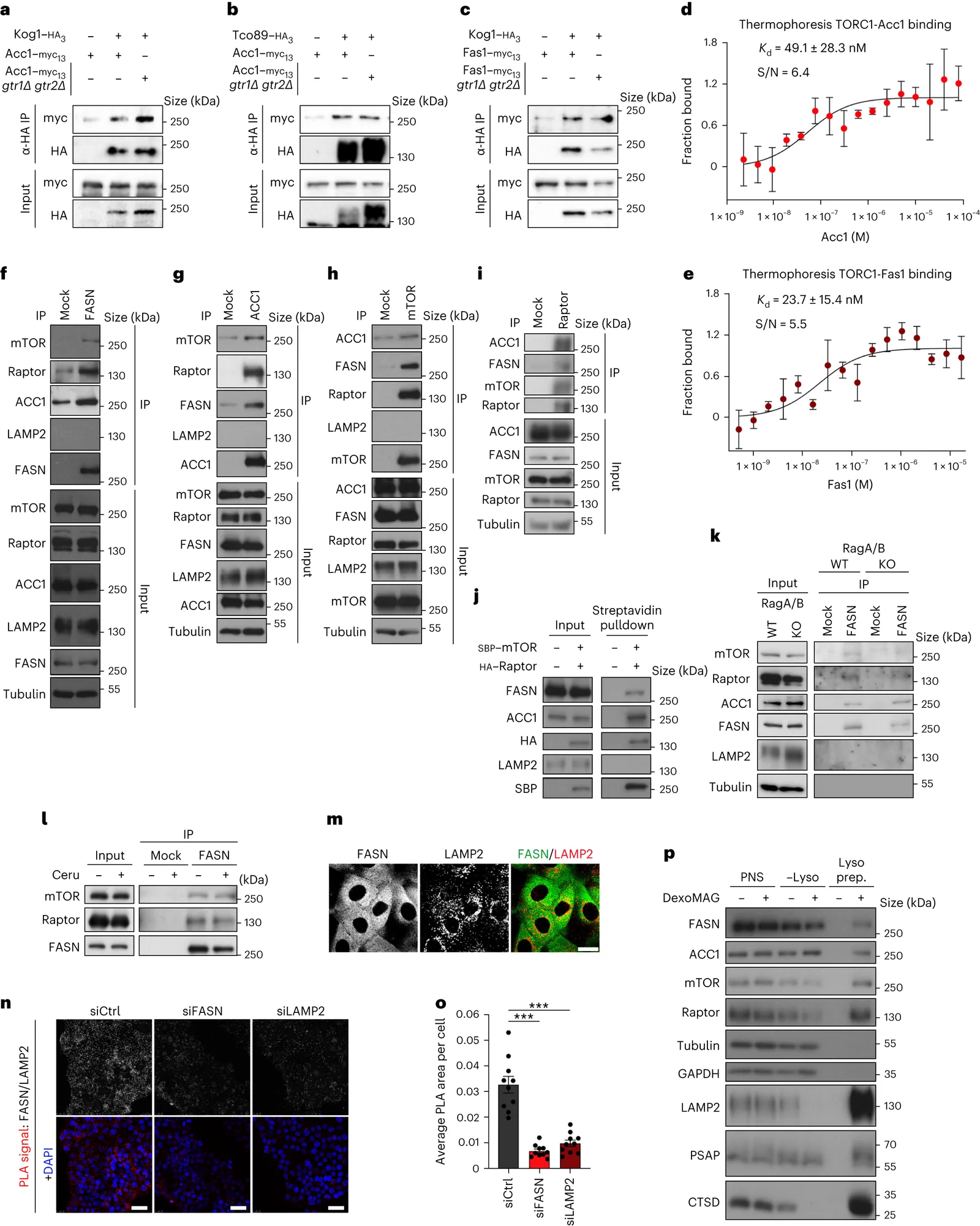
The mTORC1–FASN–ACC1 proteins form reciprocal interactions in yeast and mammalian cells. **a**,**b**, Acc1 physically interacts with TORC1 in a Rag-independent manner. Wild-type and *gtr1Δ gtr2Δ* cells expressing genomically tagged Acc1–myc_13_ and untagged (−) or genomically tagged (+) TORC1 subunits Kog1–HA_3_ (**a**) or Tco89–HA_3_ (**b**) were cultured to the exponential phase. The input and anti-HA IPs were analysed by immunoblotting (*n* = 3 independent experiments). **c**, As in **a**,**b** but with genomically tagged Fas1–myc_13_ and untagged or genomically tagged Kog1–HA_3_ (*n* = 3 independent experiments). **d**,**e**, Acc1 (**d**) and Fas1 (**e**) titration curves in MST binding affinity assays using fluorescent TORC1 as the target (*n* = 3 independent experiments). Data are the mean ± s.d.; S/N, signal-to-noise ratio. **f**, FASN interacts with mTOR, Raptor and ACC1 directly. Endogenous FASN was immunoprecipitated from HEK293FT cell lysates and the co-immunoprecipitated proteins were identified by immunoblotting as indicated. LAMP2 was used as the negative control (*n* = 8 independent experiments). **g**, As in **f** but with ACC1 immunoprecipitation (*n* = 4 independent experiments). **h**, As in **f** but with mTOR immunoprecipitation (*n* = 6 independent experiments). **i**, As in **f** but with Raptor immunoprecipitation (*n* = 3 independent experiments). **j**, Streptavidin pulldown experiments with HEK293FT cells expressing SBP–mTOR and HA–Raptor exogenously (*n* = 2 independent experiments). **k**, The interaction between FASN, mTOR/Raptor and ACC1 is independent of the Rags (*n* = 3 independent experiments using HEK293FT cells). **l**, The stability of the mTORC1–FASN interaction is not affected by FASN inhibition (*n* = 2 independent experiments using HEK293FT cells). **m**, Immunofluorescence of FASN and LAMP2 in MCF-7 cells. Scale bar, 10 μm (*n* = 3 independent experiments). **n**,**o**, FASN was detected in proximity to lysosomes. Antibodies to FASN and LAMP2 were used in PLA assays in MCF-7 cells. **n**, The specificity of the PLA signal (red dots) was verified by *FASN* and *LAMP2* knockdown using siRNA. Scale bars, 25 μm; siCtrl, control siRNA; and siLAMP2, siRNA to *LAMP2*; and DAPI, 4,6-diamidino-2-phenylindole. **o**, Quantification of PLA signal intensity (*n* = 10 randomly selected fields from one representative experiment of four independent replicates). Data are the mean ± s.e.m. ***P < 0.0005. **p**, Lysosome enrichment assay with DexoMAG for the presence of the indicated proteins in the post-nuclear supernatant (PNS), non-lysosomal fraction (–Lyso) and lysosomal fraction (Lyso prep; *n* = 5 independent experiments). IP, immunoprecipitate; α-HA, anti-HA. Source numerical data and unprocessed blots are provided. Source data

Because our data from yeast and mammalian cells indicated that the modulation of carbon flux through ACC1 and FAS/FASN affected TORC1/mTORC1 in a Rag GTPase-independent manner (Fig. 5a–l and Extended Data Figs. 7i–h, 8g,h), we entertained the idea that either or both these enzymes may directly interact with TORC1/mTORC1. In support of this assumption, we found myc-tagged Acc1 interacting with the yeast TORC1 subunits Kog1–haemagglutinin (HA) and Tco89–HA both in the presence and absence of Gtr1/2 (Fig. 6a,b). Similarly, myc-tagged Fas1 interacted with Kog1–HA even in the absence of Gtr1/2 (Fig. 6c). These co-immunoprecipitation data were also confirmed by microscale thermophoresis (MST) experiments using purified TORC1 (containing GFP–Tor1), Acc1 and Fas1 proteins from yeast cells, which demonstrated specific interactions between TORC1 and Acc1 or Fas1 at concentrations (dissociation constant (*K*_d_) = 49.1 ± 28.3 nM for TORC1-Acc1; *K*_d_ = 23.7 ± 15.4 nM for TORC1–Fas1) that are below the estimated intracellular concentrations of Acc1 (0.9–1.4 μM) and Fas1 (0.05–6.58 μM)^49–52^ (Fig. 6d,e). Notably, reciprocal interactions between endogenous FASN, ACC1, mTOR and Raptor proteins (Fig. 6f–i), or between exogenously expressed SBP-tagged mTOR (in a complex with HA-tagged Raptor) and endogenous FASN and ACC1 (Fig. 6j) were also detected in mammalian cells, with endogenous mTOR, Raptor and ACC1 co-immunoprecipitating with FASN even in *RagA*/*B*-double-knockout cells that lack an intact Rag GTPase dimer (Fig. 6k). The interaction between FASN and mTORC1 was also independent of FASN activity as it was also present in cerulenin-treated cells (Fig. 6l).

In conditions of amino-acid sufficiency, the active Rag GTPase complex recruits mTORC1 to the lysosomal surface, whereas mTORC1 demonstrates a diffuse cytoplasmic localization pattern in cells that lack a functional Rag GTPase dimer^8,41,42^. Hence, our observations that FASN inhibition downregulates mTORC1 independently of the Rags (Fig. 5a–l and Extended Data Figs. 7i–h and 8g,h) and mTOR interacts with FASN and ACC1 to the same extent in both Rag-proficient (in which mTOR is lysosomal) and Rag-deficient cells (in which mTOR is non-lysosomal) (Fig. 6k) prompted us to investigate where FASN localizes in cells. In line with the fact that FA biosynthesis takes place primarily in the cytoplasm and in agreement with publicly available protein localization databases (for example, the human protein atlas^53^), endogenous FASN immunostaining showed a diffuse cytoplasmic signal, a fraction of which co-localized with the lysosomal marker LAMP2 (Fig. 6m and Extended Data Fig. 8k). Indeed, proximity ligation assays (PLAs) using antibodies specific to endogenous FASN and LAMP2 proteins indicated that a subpopulation of FASN molecules specifically localize at, or in proximity to, the lysosomal surface (Fig. 6n,o). Furthermore, biochemical lysosome enrichment experiments using the previously established magnetic-isolation-based DexoMAG method^54–56^ further confirmed that in addition to mTOR and Raptor, FASN and ACC1—but not the equally abundant cytoplasmic/cytoskeletal proteins GAPDH and tubulin—can also be detected in lysosomal fractions (Fig. 6p).

Together, these data show that FASN and ACC1 physically interact with mTORC1 both at the lysosomal surface and away from it, suggesting that FASN inhibition and the subsequent accumulation of Mal-CoA may be directly affecting mTORC1 activity at multiple subcellular locations.

### Molecular dynamics simulations indicate that Mal-CoA binds to the mTOR catalytic pocket similarly to ATP

**Fig. 7:**
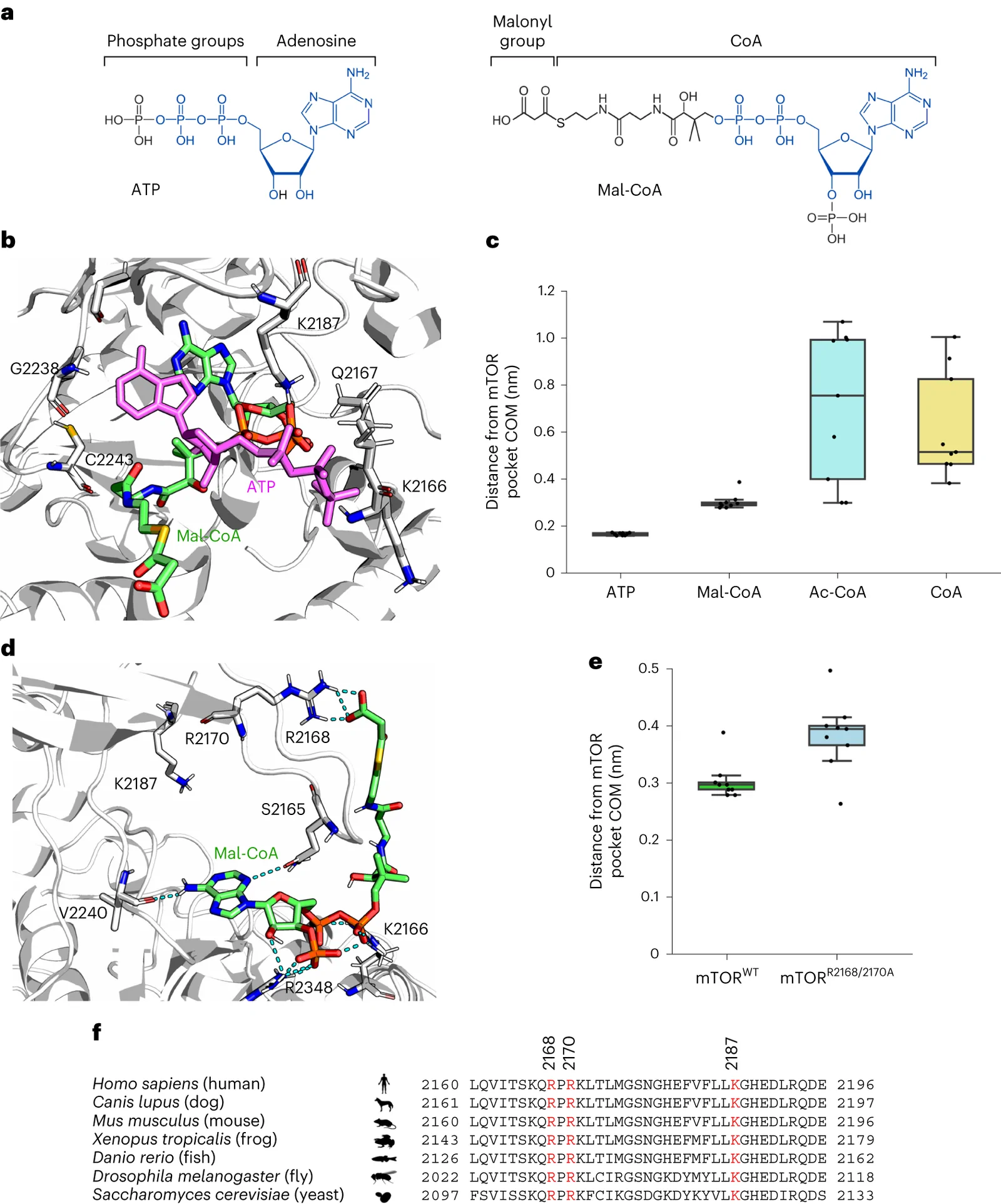
Molecular dynamics simulation of Mal-CoA binding to the mTOR catalytic pocket. **a**, Chemical structures of ATP (left) and Mal-CoA (right) highlighting structural similarities between the two molecules. Identical parts are marked in blue. **b**, Structural alignment of representative snapshots of Mal-CoA (green; initial conformation shown) and ATP (magenta) bound to the mTOR catalytic pocket (top view). **c**, Distances of the indicated ligands from the mTOR binding pocket during the molecular dynamics simulations. The distances were computed between the centre of mass (COM) of the adenine ring and the COM of the amino-acid residues defining the pocket (*n* = 9 measurements from three independent replicate runs, with three data points extracted per run for each compound). Individual data points represent the average over 100 ns of molecular dynamics simulation; Ac-CoA, acetyl-CoA. **d**, The malonyl group of Mal-CoA forms salt bridges with charged residues at the edge of the mTOR catalytic pocket (lateral view). Representative Mal-CoA (green) conformation sampled by molecular dynamics simulations. The hydrogen bonds established between Mal-CoA (final conformation in the simulation) and the amino-acid residues of the mTOR pocket are shown as cyan dotted lines. Note that only a snapshot is shown, with multiple residues participating in the formation of dynamic interactions with the malonyl group and R2168 being the most frequent. **e**, In silico mutagenesis of key mTOR residues weakens the interaction between Mal-CoA and mTOR. Distances of Mal-CoA from the mTOR binding pocket during the molecular dynamics simulations as in **c** comparing mTOR^WT^ and mTOR^R2168A/R2170A^ molecules (*n* = 9 measurements from three independent replicate runs, with three data points extracted per run). Individual data points represent the average over 100 ns of molecular dynamics simulation. **f**, Amino-acid sequence alignment of amino-acid residues 2160–2196 of human mTOR with the respective orthologous sequences from other organisms. Key conserved residues that participate in interactions with Mal-CoA are shown in red. **c**,**e**, Boxplots: central line, median; box, IQR (25th (Q1)–75th (Q3) percentile); and whiskers, Q3 + 1.5 × IQR and Q1 − 1.5 × IQR. Source numerical data are provided. Source data

**Extended Data Fig. 9:**
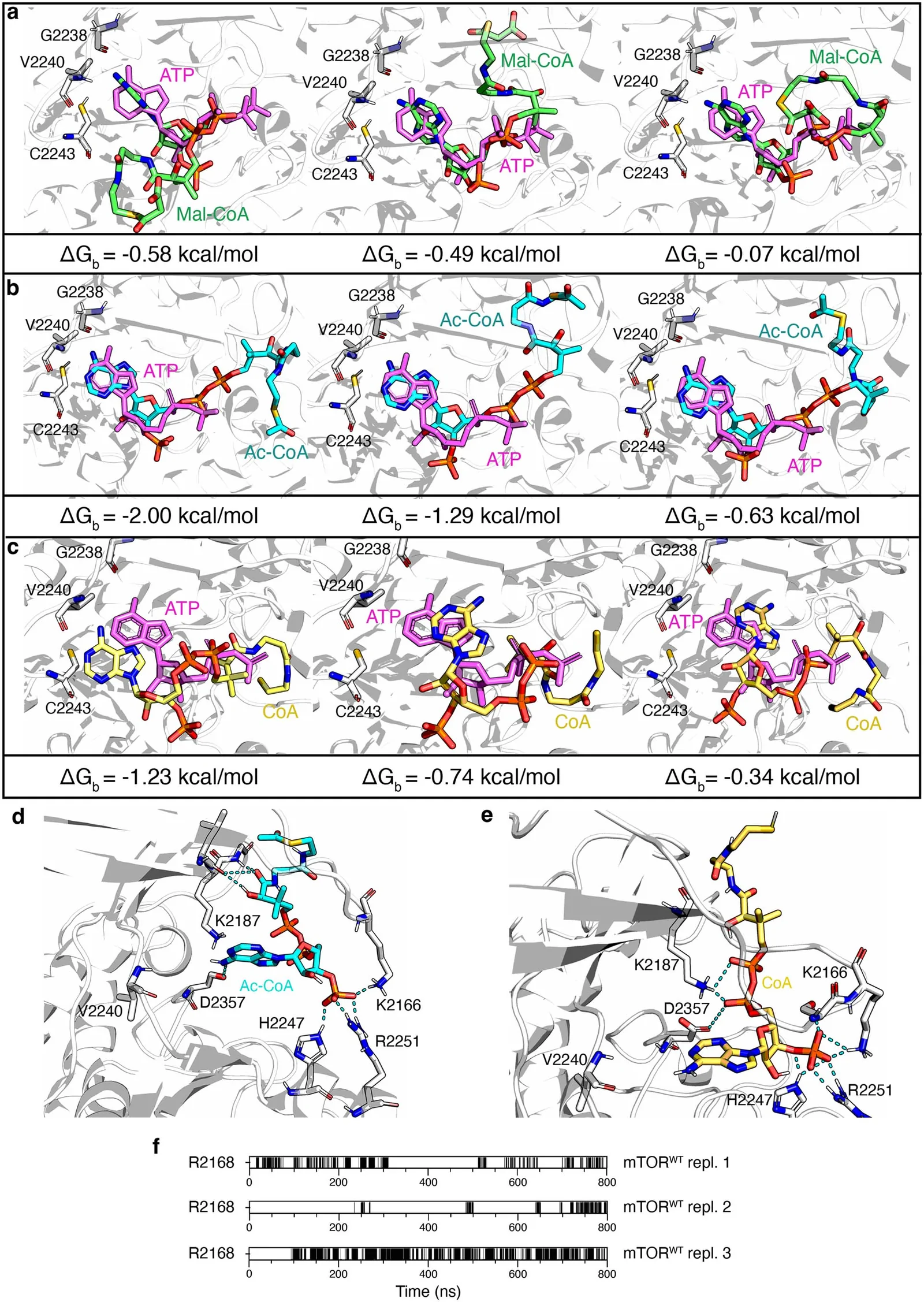
Site-specific docking simulations for Mal-CoA, Ac-CoA and CoA in the catalytic pocket of mTOR. **a**, The best three docking poses of Mal-CoA (shown in green) aligned to ATP (violet) in the mTOR catalytic pocket. The binding energy value (ΔG_b_) for each conformation is shown below the docking models. These conformations were selected as the starting point to perform all atom molecular dynamics simulations. **b**, As in **a**, but for acetyl-CoA (Ac-CoA), shown in cyan. **c**, As in **a**, but for Coenzyme A (CoA), shown in yellow. **d**, Representative acetyl-CoA (Ac-CoA) placement in the mTOR binding pocket (b). Hydrogen bonds between the compounds and the amino-acid residues of the mTOR catalytic pocket indicated by cyan dotted lines. **e**, As in **d**, but for Coenzyme A (CoA), shown in yellow. **f**, Time evolution of hydrogen bonds between the carbonyl group of Mal-CoA and the R2168 residue of wild-type mTOR are reported for the three replicates of the molecular dynamics simulations.

Our genetic, pharmacological and biochemical data hinted at a possible direct role for Mal-CoA in the inhibition of mTORC1. Taking into consideration that the adenosine moiety of Mal-CoA structurally resembles ATP (Fig. 7a), we hypothesised that Mal-CoA may be inhibiting mTOR by binding to its catalytic pocket. To investigate this possibility further, we analysed the binding of Mal-CoA, acetyl-CoA and coenzyme A (CoA) to mTOR at the atomic level by modelling the complexes starting from the crystallographic structure of mTOR bound to ATPγS (Protein Data Bank (PDB) ID 4JSP; ref. ^57^; see Methods). Given the lack of information on the interaction between these compounds and mTOR, we first hypothesised that the adenine ring present in all compounds would localize similarly to ATP in the binding pocket of the kinase domain of mTOR. Thus, the adenine ring of each compound was first aligned to that of ATP and then, to find a good arrangement of the lateral chain in the pocket, site-specific docking simulations were performed, allowing the torsion of the lateral chain only (see Methods). For each compound, three best poses were selected considering the most-favourable binding energy values (Extended Data Fig. 9a–c) and subsequently used as a starting point to perform all atom molecular dynamics simulations. As expected, ATP remained in the binding pocket during the entire simulation (Fig. 7b,c and Supplementary Video 1). Similarly to ATP, the adenine ring of Mal-CoA also remained stable in the binding pocket (Fig. 7b,c and Supplementary Video 2). In stark contrast, CoA and acetyl-CoA quickly (after 15–45 ns and 67–80 ns, respectively) detached from the protein and moved away from the binding pocket of mTOR (Fig. 7c and Supplementary Videos 3 and 4). Estimation of the relative binding free energies of these compounds using the MM/GBSA method (see Methods) confirmed that Mal-CoA binding is 6.6 ± 0.5 kcal mol^−1^ more stable than ATP, whereas acetyl-CoA binding is 2.0 ± 0.4 kcal mol^−1^ less stable than ATP. The origin of these differences in interactions between Mal-CoA and the other CoA-containing compounds with mTOR is probably due to the negatively charged chain (COOH^−^) of its malonyl group that can engage in interactions with positively charged mTOR residues just outside the binding pocket, most notably R2168 in the molecular dynamics simulations, but possibly also with the nearby residues R2170 and K2187 (Fig. 7d). In contrast, the respective acetyl-CoA (-CH_3_) and CoA (-SH) groups are not charged and, thus, do not engage in similar interactions (Extended Data Fig. 9d,e). Accordingly, molecular dynamics simulations and in silico mutagenesis of mTOR at the R2168 and R2170 residues (mTOR^R2168A/R2170A^), which seemingly participate in the stabilization of Mal-CoA, hinted at a possible role for these residues in Mal-CoA binding (Fig. 7d,e, Extended Data Fig. 9f and Supplementary Video 5). Intriguingly, these residues are strongly conserved in mTOR over a wide range of organisms spanning from yeast to humans (Fig. 7f).

### Mal-CoA is a direct ATP-competitive inhibitor of mTORC1 activity

**Fig. 8:**
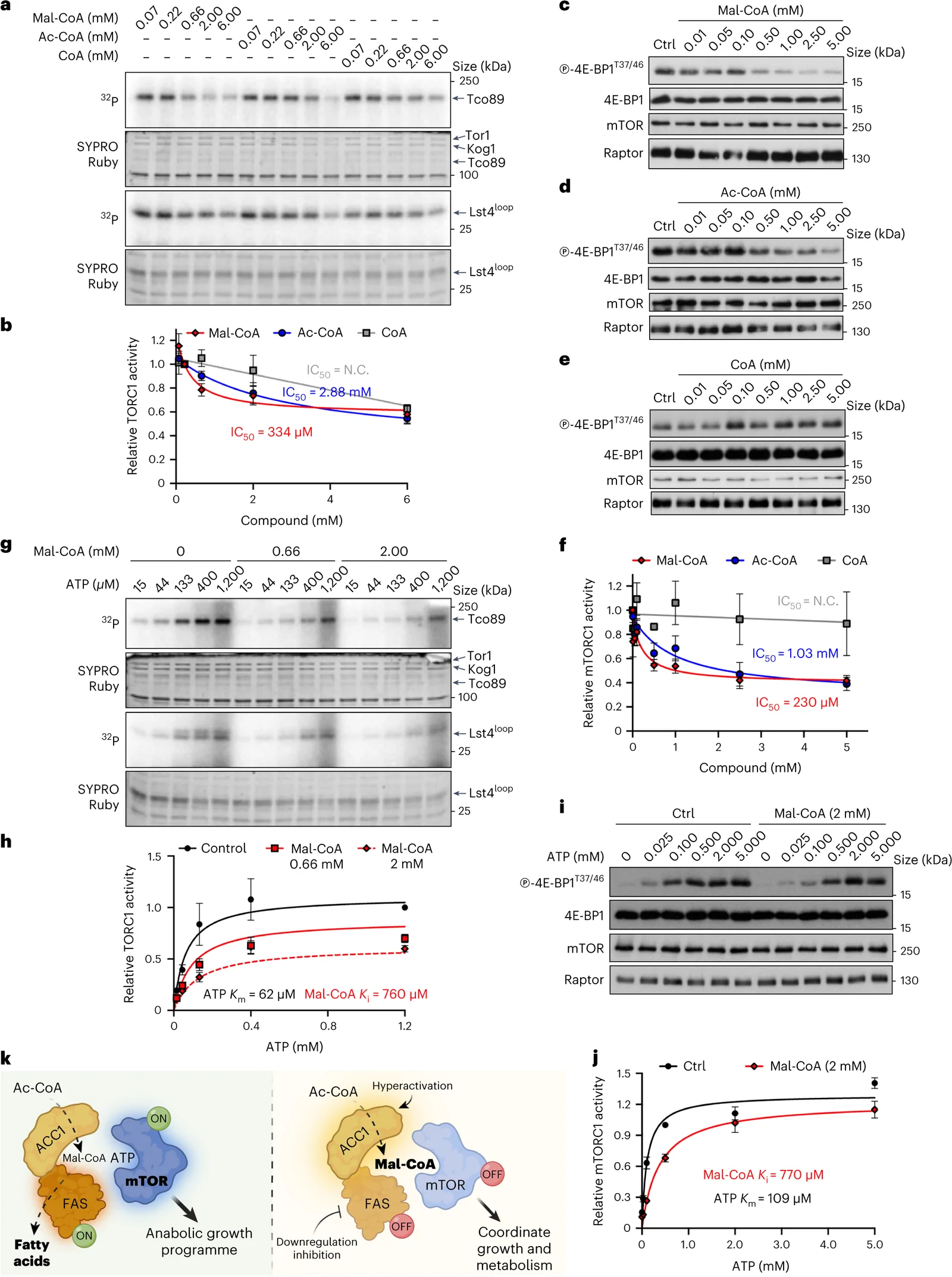
Mal-CoA is a direct ATP-competitive inhibitor of mTORC1. **a**,**b**, Mal-CoA, and to a lesser extent acetyl-CoA (Ac-CoA), inhibits TORC1 activity in vitro. TORC1 purified from yeast was used in IVK assays with recombinant Lst4^Loop^ and co-purified Tco89 proteins as substrates in the presence of increasing concentrations of Mal-CoA, Ac-CoA or CoA. **a**, Substrate phosphorylation was detected using autoradiography (^32^P). Total protein was detected by SYPRO Ruby staining. **b**, Calculated levels of TORC1 activity (Lst4^Loop^ phosphorylation; *n* = 3 independent experiments). **c**–**f**, mTORC1 purified from HEK293FT cells was used in IVK assays as in **a** with recombinant 4E-BP1 protein as the substrate in the presence of increasing concentrations of Mal-CoA (**c**), Ac-CoA (**d**) or CoA (**e**). **c**–**e**, Phosphorylation of 4E-BP1 was detected by immunoblotting. **f**, Levels of mTORC1 activity (*n*_Mal-CoA_ = 9, *n*_Ac-CoA_ = 6 and *n*_CoA_ = 4 independent experiments). **g**,**h**, Mal-CoA inhibits TORC1 in an ATP-competitive manner. Increasing ATP concentrations with or without Mal-CoA were used in IVK assays performed as in **a**. **h**, Levels of TORC1 activity (Lst4^Loop^ phosphorylation; *n* = 3 independent experiments). **i**,**j**, Increasing ATP concentrations in the presence or absence of 2 mM Mal-CoA were used in IVK assays performed as in **c**. **j**, Levels of mTORC1 activity (*n* = 4 independent experiments). **k**, Model of mTOR inhibition by Mal-CoA. When the FA biosynthesis machinery is active, ACC1 converts Ac-CoA to Mal-CoA, which is in turn rapidly converted to palmitate by FASN (left). In contrast, when ACC1 is hyperactive or FASN is downregulated, accumulating Mal-CoA competes with ATP for binding to proximal mTOR molecules, causing their inactivation. Hence, by complexing with ACC1 and FASN, mTORC1 functions as a direct sensor for Mal-CoA to adjust growth and coordinate cellular metabolic activity in response to decreased cellular FA biosynthesis capacity (right). **b**,**f**,**h**,**j**, Data are the mean ± s.e.m. Ctrl, control; N.C., not calculated. Source numerical data and unprocessed blots are provided. Source data

**Extended Data Fig. 10:**
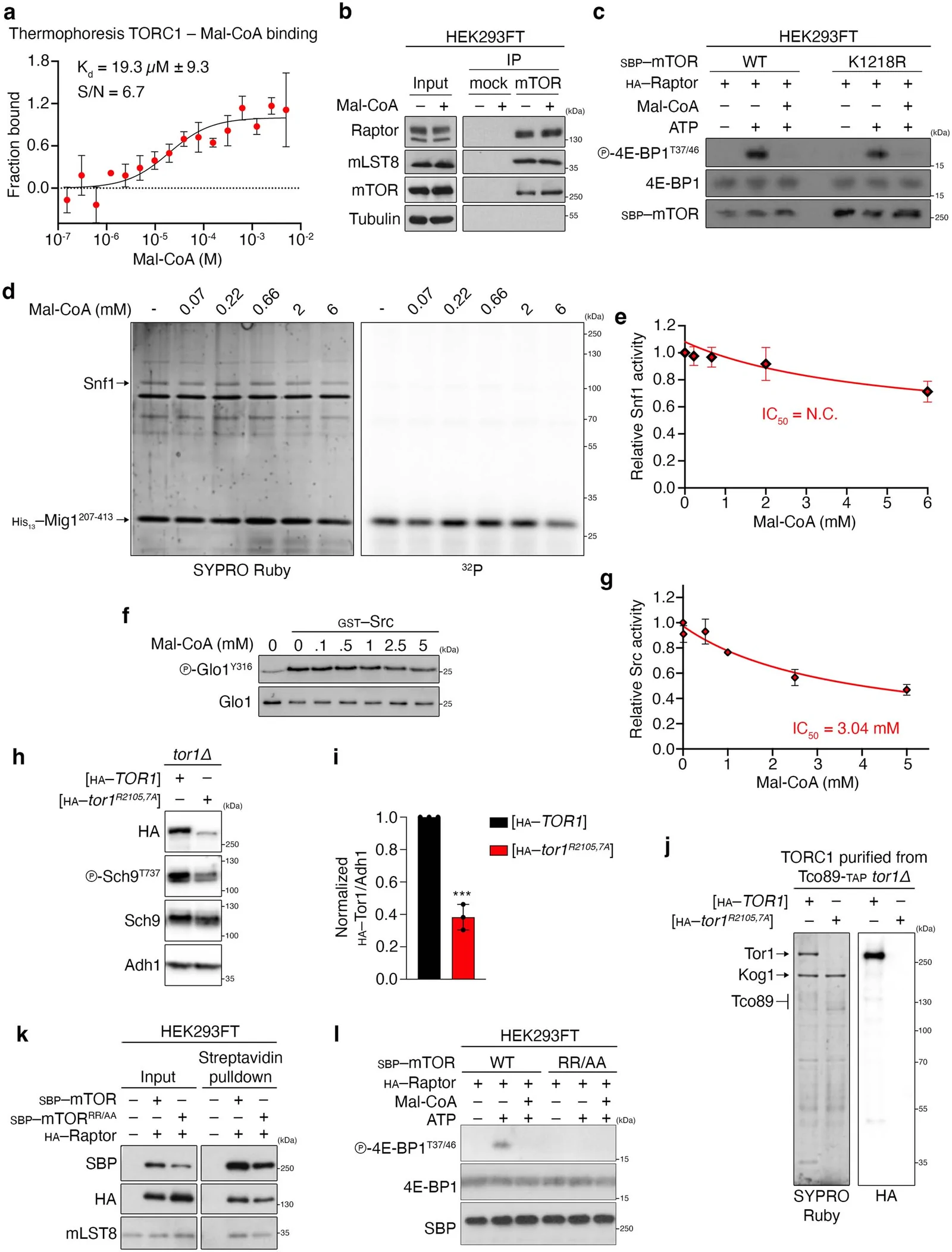
Mal-CoA binds and specifically inhibits mTORC1 without affecting complex composition and independently of mTOR malonylation, while mutant Tor1/mTOR in the Mal-CoA stabilization residues show decreased stability and/or activity. **a**, Microscale thermophoresis experiment using purified TORC1 (containing GFP–Tor1) from yeast cells, confirming binding of Mal-CoA to TORC1; *n* = 3 independent experiments. **b**, Mal-CoA does not affect mTORC1 complex stability. Endogenous mTOR was immunoprecipitated in the presence or absence of 1 mM Mal-CoA (added directly in the lysates 5 min previous to addition of the antibody). Co-immunoprecipitation of Raptor and mLST8 assayed by immunoblotting; *n* = 2 independent experiments. **c**, Mal-CoA inhibits mTORC1 independently from mTOR malonylation. IVKs as in Fig. 8c using SBP-tagged WT or K1218R mutant mTOR and HA–Raptor in the presence or absence of 5 mM Mal-CoA. Reactions omitting ATP were used as negative controls; *n* = 2 independent experiments. **d**,**e**, Mal-CoA does not inhibit Snf1 in vitro. **d**, IVK assays as in Fig. 8a, but using purified Snf1 and His-tagged Mig1 as substrate, with the indicated concentrations of Mal-CoA. **e**, Quantification of Snf1 activity; *n* = 3 independent experiments. **f**,**g**, Src IVK assay as in Fig. 8c using Glo1 as a substrate, with the indicated amounts of Mal-CoA. **g**, Quantification of Src activity; *n* = 3 independent experiments. **h**,**i**, Stability of HA-tagged Tor1^R2105/2107A^ mutant in vivo. **i**, Quantification of Tor1 protein levels normalized to Adh1; *n* = 3 independent experiments. **j**, In vitro stability of HA-tagged Tor1^R2105/2107A^ mutant purified from yeast cells via TAP-mediated pulldown of Tco89; *n* = 2 independent experiments. **k**, The human SBP-tagged mTOR R2168A/R2170A mutant (mTOR^RR/AA^) is relatively stable and binds other mTORC1 components similarly to WT mTOR. Streptavidin pulldown of WT or mTOR^RR/AA^ detecting binding to HA–Raptor and endogenous mLST8; *n* = 2 independent experiments. **l**, The mTOR^RR/AA^ mutant lacks catalytic kinase activity in vitro. IVKs as in Fig. 8c using SBP-tagged WT or mTOR^RR/AA^ and HA–Raptor in the presence or absence of 5 mM Mal-CoA. Reactions omitting ATP were used as negative controls; *n* = 2 independent experiments. Data are the mean ± s.d. (**a**) or mean ± s.e.m. (all other graphs). ***P < 0.0005. Source numerical data and unprocessed blots are provided. Source data

To experimentally test our in silico analyses, we next sought to investigate whether Mal-CoA is able to bind mTORC1 and inhibit its activity directly in a cell-free system. Microscale thermophoresis experiments using purified yeast TORC1 (containing GFP–Tor1) confirmed binding of Mal-CoA to TORC1 with *K*_d_ = 19.3 μM ± 9.3 (Extended Data Fig. 10a), providing support for our molecular dynamics simulation studies. Strikingly, in classical in vitro enzyme kinetics assays using TORC1 purified from yeast cells and recombinant Lst4 (that is, Lst4^Loop^ described in ref. ^58^) or co-purified Tco89 (ref. ^59^) proteins as substrates, we observed that the addition of Mal-CoA to the in vitro kinase (IVK) reaction inhibited TORC1 in a dose-dependent manner with a calculated half maximal inhibitory concentration (IC_50_) of 334 μM, whereas the IC_50_ values for acetyl-CoA and CoA were 2.88 mM and above our detection limit of 6 mM, respectively (Fig. 8a,b). Accordingly, mTORC1 IVK assays using immunoprecipitated mTORC1 from mammalian cells and recombinant 4E-BP1 as substrate, and increasing amounts of Mal-CoA, acetyl-CoA and CoA revealed a dose-dependent inhibition of mTORC1 by Mal-CoA (IC_50_ = 230 μM), with acetyl-CoA being substantially less potent (IC_50_ = 1.03 mM) and CoA unable to inhibit mTORC1 activity under our experimental conditions (IC_50_ > 5 mM; Fig. 8c–f). As a control, the addition of Mal-CoA to cell lysates before immunoprecipitation did not influence mTORC1 stability, as indicated by the interaction of mTOR with Raptor and mLST8 proteins (Extended Data Fig. 10b). Furthermore, in agreement with mTOR not being malonylated in our system (Fig. 4f,g), the kinase activity of an mTORC1 complex containing a non-malonylatable mTOR^K1218R^ mutant^39^ was readily inhibited by Mal-CoA, similarly to wild-type mTOR (Extended Data Fig. 10c). Pointing against a role of Mal-CoA being a generic non-specific kinase inhibitor, this metabolite did not demonstrate substantial inhibitory effects against two other kinases, the yeast AMPK orthologue Snf1 (IC_50_ = N.C., not calculated) and human Src (IC_50_ = 3.04 mM) in IVK assays (Extended Data Fig. 10d–g). Overall, our data confirm that Mal-CoA (and to a lesser extent acetyl-CoA) can act as a direct mTORC1 inhibitor, without affecting complex composition and independently of mTOR malonylation.

We were unfortunately unable to experimentally test the role of the R2105 and R2107 residues of yeast Tor1 (residues R2168 and R2170 in human mTOR) in Mal-CoA binding, as the respective Tor1^R2105A/R2107A^ double alanine-mutant protein was extremely unstable both in cells (Extended Data Fig. 10h,i) and when we attempted to purify TORC1 by immunoprecipitation of TAP-tagged Tco89 (Extended Data Fig. 10j). Furthermore, although the human mTOR^R2168A/R2170A^ double mutant was relatively stable and bound Raptor and mLST8 similarly to the wild-type protein (Extended Data Fig. 10k), it completely lacked kinase activity in IVK assays (Extended Data Fig. 10l). Therefore, in addition to participating in the stabilization of Mal-CoA in the mTOR catalytic pocket (as shown from our in silico mutagenesis and molecular dynamics simulation analyses), these residues seemingly also play important roles in mTOR stability and mTORC1 kinase activity.

The resemblance of Mal-CoA to ATP and our molecular dynamics simulation experiments suggested that Mal-CoA may inhibit TORC1/mTORC1 directly through competition with ATP. To test this hypothesis, we performed IVK assays with increasing ATP concentrations using yeast TORC1 (Fig. 8g,h) or human mTORC1 complexes (Fig. 8i,j). When subjected to regression analysis, using the GraphPad Prism curve fitting program, the IVK data indicated that the behaviour of Mal-CoA matched best with that of an ATP-competitive TORC1/mTORC1 inhibitor with a calculated inhibition constant (*K*_i_) of 760 μM and 770 μM for yeast and human complexes, respectively (Fig. 8h,j). In summary, our data reveal that Mal-CoA is a direct and ATP-competitive inhibitor of mTORC1 in both yeast and human cells, thus serving as a key metabolite that directly connects the cellular FA biosynthetic capacity to the activity of the main cellular metabolic regulator (Fig. 8k).

## Discussion

A key characteristic of mTORC1 is that it forms homeostatic feedback loops, acting both as a molecular sensor and a regulator of individual biosynthetic processes. For instance, mTORC1 is the master controller of protein synthesis via direct phosphorylation of S6K and 4E-BP1, while it also senses the sufficiency of amino acids and energy, thus ensuring that cells only make proteins when all building blocks are available^2^. mTORC1 was previously described to regulate lipid biosynthesis at several levels by controlling the activity and localization of Lipin-1 (refs. ^60–63^) and the activity of SREBP transcription factors^64,65^. Here we report that this interplay also happens in the opposite direction, with key components of the core FA biosynthesis machinery (namely ACC1 and FASN) interacting directly with mTOR and Raptor, and regulating mTORC1 activity via changes in Mal-CoA levels. This way the FA biosynthesis capacity of a cell is closely coupled to cell growth, metabolism and other downstream cellular functions of mTORC1 like protein synthesis (Figs. 2l and 8k). Accordingly, SREBPs are the best-described transcription factors controlling FASN expression^66,67^. By mTORC1 regulating SREBP activity, and thereby FASN levels^11^, our findings reveal a positive-feedback loop between mTORC1 and FASN that could function to sustain lipid biosynthesis when conditions are optimal and mTORC1 is active (thus maintaining high FASN). This machinery is likely to also be relevant in adipocyte differentiation: pre-adipocytes have low mTORC1 activity and low FASN expression. In response to adipogenic stimuli, FASN levels increase massively (10- to 15-fold in 3T3-L1 cells^68^) to support FA biosynthesis and differentiation to adipocytes, which correlates with increased mTORC1 activity. Notably, both blockage of FASN activity^69,70^ and mTORC1 inhibition^71,72^ prevent adipogenesis, thus highlighting the importance of a coordinated metabolic response for this process. Future work will be needed to identify the physiological settings in which the machinery that we describe in this study is involved.

Endogenous metabolites are known to control the activity of key signalling molecules by directly binding to them and modifying their structure and function. For instance, binding of four cAMP molecules to the two regulatory subunits of protein kinase A (PKA) causes their dissociation from the catalytic PKA subunits, which are then activated and directly regulate downstream effectors, such as the CREB transcription factor, to modulate cellular metabolism^73–75^. Similarly, under low-energy conditions, AMP allosterically activates AMPK by binding directly to its γ subunit^17^. Along the same lines, a recent study showed that inositol directly competes with AMP for binding to AMPKγ, thereby allosterically inhibiting AMPK enzymatic activity, with low inositol driving the AMPK-dependent mitochondrial fission following energetic stress^18^. Our work here describes another example of an endogenous metabolite (that is, Mal-CoA) that functions as a direct regulator of a central signalling complex (that is, mTORC1). However, unlike the allosteric modulation of kinase activities described above for PKA and AMPK, Mal-CoA acts as an ATP-competitive mTORC1 inhibitor, an attribute that stems from the structural similarity between the CoA moiety and ATP. Molecular dynamics simulations also support a model where the charged malonyl group helps stabilize Mal-CoA binding to mTOR via interactions with residues just outside its catalytic pocket, and may provide an explanation for the specific inhibitory effect of Mal-CoA towards mTORC1 but not towards other kinases. Notably, unlike for amino-acid availability that signals to mTORC1 via a complex upstream signalling network that impinges on the Rag GTPases^2^, mTORC1 senses the capacity of a cell to make FAs directly by Mal-CoA competing with ATP for binding to its catalytic pocket. Importantly, we find that this is an ancient mechanism that is present already in yeast cells and is conserved through evolution all the way to humans.

Although the absolute concentrations of Mal-CoA in yeast and mammalian cells have not been accurately determined to date, mainly due to technical limitations and the low stability of this metabolite, our IVK experiments show that Mal-CoA inhibits mTORC1 with IC_50_ = 230 μM and *K*_i_ = 770 μM (IC_50_ = 334 μM and *K*_i_ = 760 μM for yeast TORC1). These concentrations are in the same range of those previously determined for the activity of recombinant human MCD (Mal-CoA decarboxylase), which binds Mal-CoA in vivo to convert it to acetyl-CoA with an apparent Michaelis constant (*K*_m_) value of approximately 220–520 μM (refs. ^76–78^). Furthermore, the direct physical association between ACC1/FASN and mTOR/Raptor suggests that, following FASN blockage or ACC1 hyperactivation, a local increase in Mal-CoA levels could inhibit proximal mTORC1. Because FASN can interact with both lysosomal and non-lysosomal mTOR, perturbations to its activity are able to control all subpopulations of mTORC1 in cells. Although such metabolic proximity channelling principles have been described before^79^ and can facilitate efficient transfer of a metabolite from one enzyme to the next, thus bypassing the need for alterations in total intracellular metabolite levels, whether the formation of an FASN–ACC1–mTORC1 hypercomplex is in fact required for the observed effects of Mal-CoA to mTORC1 inhibition remains to be demonstrated. Such work may also help explain the apparently paradoxical specificity of Mal-CoA towards mTORC1 and the lack of an effect towards mTORC2 in cells, given that both complexes contain the same kinase. One possible scenario is that this specificity may stem from complex-specific subunits (for example, Raptor versus Rictor) contributing to the stabilization of Mal-CoA in the mTOR catalytic pocket or by differential protein–protein interactions of the two complexes to FASN and/or ACC1. Follow-up biochemical and in silico studies will be necessary to shed light on these important open questions.

Because of its central role in FA biosynthesis, FASN has emerged as a critical player in cancer cell metabolism, growth and survival^13,80–82^, with several FASN inhibitors currently being tested in clinical trials^83^. Interestingly, previous work suggested that the accumulation of Mal-CoA, rather than the inhibition of FASN itself, is the underlying cause in FASN-inhibitor-induced toxicity in breast cancer cells^34^. In this study we found that FASN inhibition also leads to mTORC1 downregulation due to Mal-CoA accumulation in addition to its well-known role in FA biosynthesis. As mTOR activity is commonly dysregulated in the majority of human cancers, our work raises the plausible hypothesis that part of the beneficial effect of FASN inhibition in cancer treatment may be due to the concomitant drop in mTORC1 signalling^84^. Notably, mTORC1 and FASN inhibitors demonstrated synergistic effects in yeast growth in our experiments, even when combined at sublethal doses for each individual compound. These data are in agreement with a previous report showing synthetic lethality of cerulenin and rapamycin in cancer cell lines^85^. In summary, our findings identify a direct connection between the core FA biosynthesis machinery and mTORC1 activity, reveal unique concepts of how metabolic signalling is coordinated in cells and provide the basis for the development of advanced therapeutic tools to treat human conditions that are linked to hyperactive mTORC1 signalling.

## Methods

### Yeast culture

Yeast cells (*Saccharomyces cerevisiae*) were cultured in liquid SC medium (1.7 g l^−1^ yeast nitrogen base (cat. no. 1545, CONDA), 5 g l^−1^ ammonium sulfate (cat. no. 4808211, MP Biomedicals), 20 g l^−1^ glucose (cat. no. 1422, AppliChem), 2 g l^−1^ amino acid dropout –His (D9520, US Biological) and 35 mg l^−1^ histidine (A1341, AppliChem) at 30 °C to the exponential growth phase, unless otherwise stated in the figure legends. All yeast strains used in this study are listed in Supplementary Table 1.

### Yeast culture treatments

For the starvation/re-addition experiments, yeast cells growing in exponential phase were filtered and shifted to prewarmed (30 °C) nitrogen-starvation medium (1.7 g l^−1^ yeast nitrogen base and 20 g l^−1^ glucose) for 20 min. Subsequently, glutamine (cat. no. 119951000, Acros) was added to a final concentration of 3.3 mM (using a 50× stock solution). Treatment with the pharmacological FAS inhibitor cerulenin (C2389, Sigma-Aldrich) was carried out by adding the drug directly to the cell cultures at the concentration and times indicated in the figure legends.

### Mammalian cell culture

All cell lines were cultured at 37 °C and 5% CO_2_. Human female embryonic kidney HEK293FT cells (R70007, Invitrogen; Research Resource Identifier (RRID): CVCL_6911), human female breast adenocarcinoma MCF-7 cells (HTB-22, ATCC; RRID, CVCL_0031), immortalized mouse embryonic fibroblasts, human female bone osteosarcoma U2OS cells (HTB-96, ATCC; RRID, CVCL_0042) and human female embryonic kidney HEK293T cells (RRID, CVCL_0063) were cultured in high-glucose Dulbecco’s modified eagle medium (DMEM; 41965-039, Gibco) supplemented with 10% FBS (F7524, Sigma; S1810, Biowest). MCF-7 cells were also supplemented with 1×non-essential amino acids (11140-035, Gibco). Human male diploid lung WI-26 SV40 fibroblasts (WI-26 cells; CCL-95.1, ATCC; RRID, CVCL_2758) were cultured in DMEM/F12 GlutaMAX medium (cat. no. 31331093, Thermo Fisher Scientific) containing 10% FBS. All media were supplemented with 1×penicillin–streptomycin (15140-122, Gibco).

HEK293FT cells were purchased from Invitrogen. Control immortalized mouse embryonic fibroblasts were a gift from K.-L. Guan (described in ref. ^86^). U2OS cells were a gift from N.-G. Larsson (Karolinska Institute). HEK293T cells were a gift from M. Boutros (DKFZ). The identity of the WI-26 cells was validated using the Short Tandem Repeat (STR) profiling service provided by Multiplexion GmbH. The identity of the HEK293FT and MCF-7 cells was validated with a Multiplex human cell line authentication test (Multiplexion GmbH), which uses a single nucleotide polymorphism-typing approach and was performed as described at www.multiplexion.de. No commonly misidentified cell lines were used in this study. All cell lines were regularly tested for *Mycoplasma* contamination, using a PCR-based approach and were confirmed to be *Mycoplasma*-free.

### Mammalian cell culture treatments

Amino-acid-starvation experiments were carried out as described before^42,43^. Treatments with the pharmacological FASN inhibitors Fasnall (SML1815, Sigma-Aldrich) and cerulenin (C2389, Sigma-Aldrich) as well as with the MEK1/2 inhibitor U0126 (cat. no. 9903, Cell Signaling Technology) and rapamycin (S1039, Selleckchem) were performed by adding the drugs directly to full, amino-acid- and FBS-containing culture media (described in ‘Mammalian cell culture’) at the concentrations and duration indicated in the figure legends; DMSO (4720.1, Roth) was used as the control. For the lipid depletion experiments, cells were cultured in medium containing 10% charcoal-stripped FBS (A3382101, Thermo Fisher Scientific), instead of full FBS, for 24 h before treatments with FASN inhibitors were performed. For FA supplementation, each FA was first conjugated to 10% FA-free BSA (fraction V; cat. no. 10735086001, Roche) for 1 h at 50 °C in a 50:50 volumetric ratio. The BSA-conjugated C16:0, C16:1, C18:1 and C18:2 FAs (100 μM) were then added to the media both 16 h previous to and at the start of the treatment with FASN inhibitors. Exogenous Mal-CoA treatments were performed by adding 250 μM Mal-CoA lithium salt (M4263, Sigma-Aldrich) to the culture medium for 30 min before cell lysis.

### Antibodies

All of the antibodies used in this study are listed in Supplementary Table 2.

### Plasmids and molecular cloning

The pcDNA3-FLAG–hRagA^QL^ (Q66L) and hRagC^SN^ (S75N) vectors expressing the constitutively active Rag GTPases were described previously^42^. The pcDNA3-FLAG–Luc control vector was previously described^87^. The pRK5-HA–RAPTOR (plasmid no. 8513; RRID, Addgene_8513; described in ref. ^88^) and pSpCas9(BB)-2A-Puro (PX459) V2.0 plasmids were obtained from Addgene (plasmid no. 62988; RRID, Addgene_62988; described in ref. ^89^).

To generate the pcDNA3-FLAG–ACC1 expression vector, the long ACC1 isoform 4 (UniProt ID Q13085-4; not used in this study) was PCR amplified from complementary DNA (prepared from MCF-7 cells) using the appropriate primers and cloned in the XhoI/XbaI restriction sites of pcDNA3-FLAG. Next, the canonical, shorter ACC1 isoform 1 (UniProt ID, Q13085-1) was generated using the isoform 4 expression vector as a template and the appropriate PCR primers, and cloned in the XhoI/BglII restriction sites of pcDNA3-FLAG–ACC1^iso4^, thus replacing the long N-terminal part with that of ACC1 isoform 1. The respective pcDNA3-FLAG–ACC1^S79A^ (isoform 1) plasmid was generated by site-directed mutagenesis using appropriate primers, and the insert was cloned in the XhoI/BglII restriction sites of pcDNA3-FLAG–ACC1^iso1^.

The pETM-11-4E-BP1 vector, used to express His_6_-tagged 4E-BP1 in bacteria, was generated by PCR-amplifying human 4E-BP1 from cDNA (prepared from HEK293FT cells) using the appropriate primers and cloned in the NcoI/NotI restriction sites of pETM-11.

For the pcDNA4/TO/SBP–mTOR expression vector, the streptavidin-binding peptide (SBP) tag sequence was first amplified from Str-KDEL_ManII-SBP-EGFP (Addgene plasmid no. 65252; RRID, Addgene_65252; described in ref. ^90^) and cloned into the KpnI/BamHI restriction sites of pcDNA4/TO/Myc-His A. Next, human mTOR cDNA was amplified from pcDNA3-FLAG–mTOR (Addgene plasmid no. 26603; RRID, Addgene_26603; described in ref. ^91^) using the appropriate primers and cloned in-frame into the NotI/AgeI restriction sites of the pcDNA4/TO/SBP vector. The respective SBP–mTOR^K1218R^ and SBP–mTOR^RR/AA^ (R2168A/R2170A) constructs were generated using GeneArt Strings (Thermo Fisher Scientific) containing the respective mutations to replace either the KasI/BstEII fragment (for SBP–mTOR^K1218^) or the BstEII/HpaI fragment (for SBP–mTOR^RR/AA^) of WT mTOR in the pcDNA4/TO/SBP–mTOR construct.

The integrity of all constructs was verified by sequencing. All of the DNA oligonucleotides used in this study are listed in Supplementary Table 3.

### mRNA isolation and cDNA synthesis

Total mRNA was isolated from cells using a standard TRIzol–chloroform-based method (cat. no. 15596018, Thermo Fisher Scientific) according to the manufacturer’s instructions. For cDNA synthesis, mRNA was transcribed to cDNA using a RevertAid H minus first strand cDNA synthesis kit (K1631, Thermo Fisher Scientific) or Maxima H minus reverse transcriptase (EP0753, Thermo Fisher Scientific) and an oligodT primer, according to the manufacturer’s instructions.

### Gene expression analysis

For quantitative real-time PCR experiments, the cDNA was diluted 1:20 in nuclease-free water and 4 µl were used per reaction, together with 5 µl primaQUANT CYBR 2× qPCR SYBRGreen master mix with LOW ROX (SL-9913, Streinbrenner) and 1 µl primer mix (2.5 µM of forward and reverse primers). Reactions were set up as technical triplicates in a StepOnePlus Real-Time PCR system (Applied Biosystems). Relative gene expression was calculated using the 2^−ΔΔ*C*t^ method, with β-actin as the loading control, and normalized to the expression of the gene in the respective control sample. All of the quantitative PCR primers used in this study are listed in Supplementary Table 3.

### Plasmid DNA transfections

Plasmid DNA transfections in HEK293FT cells were performed using Effectene transfection reagent (cat. no. 301425, Qiagen) or X-tremeGENE HP (cat. no. 6366236001, Sigma-Aldrich; for IVK experiments with SBP–mTOR) according to the manufacturer’s instructions.

### Generation of knockout cell lines

The HEK293FT *RagA*/*B*-, *RagC*/*D*- and *TSC1*-knockout cell lines were generated using the pX459-based CRISPR–Cas9 method, as described elsewhere^89^. The single guide RNA expression vectors were generated by cloning the appropriate DNA oligonucleotides (Supplementary Table 3) in the BbsI restriction sites of pX459 (cat. no. 62988, Addgene). An empty pX459 vector was used to generate matching negative control cell lines. Briefly, transfected cells were selected with 3 μg ml^−1^ puromycin (A11138-03, Thermo Fisher Scientific) 48 h post transfection. Single-cell clones were generated by single-cell dilution and knockout clones were validated by immunoblotting. The HEK293T *DEPDC5* and *RagA*/*B*-triple-knockout cell line has been described previously^92^. The HEK293T *AMPKα1*/*2*-knockout cell line was generated by co-transfecting two pX459-based single guide RNA expression vectors targeting both isoforms of AMPKα. After selection with 1 μg ml^−1^ puromycin for 3 d, the transfected cells were seeded in 96-well plates at a dilution of 1 cell per well and clones were screened by immunoblotting for AMPKα and phospho-ACC levels.

### Gene silencing experiments

Transient knockdown of *FASN* was performed using a pool of four siGENOME siRNAs (Horizon Discoveries). An siRNA duplex targeting the *Renilla reniformis* luciferase gene (RLuc; P-002070-01-50, Horizon Discoveries) was used as the control. Transfections were performed using 20 nM siRNA and Lipofectamine RNAiMAX transfection reagent (cat. no. 13778075, Thermo Fisher Scientific) according to the manufacturer’s instructions. The cells were harvested or fixed 48 h post transfection and knockdown efficiency was verified by immunoblotting.

### Targeted re-analysis of SATAY experiments

Targeted re-analyses were performed using recently published SATAY datasets^24,25^. The number of transposition events (reads) in the various libraries, corresponding to cells treated with rapamycin^24^, cerulenin or soraphen A^25^, and their respective untreated controls (reference libraries) was normalized to the total number of transposons mapped in the library, with the addition of a noise factor of 0.1. For each gene, the fold change was calculated as the normalized number of reads per gene in the experimental set divided by that in the reference set. These analyses showed that transposition events in TORC1-pathway genes were under-represented when cells were cultured in the presence of rapamycin (as expected) and cerulenin. On the contrary, transposition events were over-represented when cells were cultured in the presence of soraphen A. These results show that FAS inhibition with cerulenin correlates positively with rapamycin treatment and negatively with the Acc1 inhibition by soraphen A treatment.

### Yeast genetic manipulation

Site-directed mutagenesis in yeast was performed using CRISPR–Cas9 according to a previously described method^93^, with minor optimizations. Gene deletions and genomic tagging were performed with a standard high-efficiency transformation protocol using cassettes amplified from various plasmids of the pFA6a PCR toolbox^94^ or by mating and tetrad dissection. Plasmid mutagenesis was performed using a QuikChange multi site-directed mutagenesis kit (Agilent) according to the manufacturer’s protocol. See Supplementary Table 4 for the full list of the plasmids used in the yeast experiments.

### Yeast cell lysis and immunoblotting

For yeast protein extractions, 10 ml of the cell culture was mixed with trichloroacetic acid at a final concentration of 6%. After centrifugation, the pellet was washed with cold acetone and dried in a SpeedVac concentrator. The pellet was resuspended with an amount of lysis buffer (50 mM Tris–HCl pH 7.5, 5 mM EDTA, 6 M urea and 1% SDS) that was proportional to the optical density at 600 nm of the original cell culture. Proteins were extracted by disruption in a Precellys machine in the presence of glass beads. Subsequently, a Laemmli-based sample buffer (350 mM Tris–HCl pH 6.8, 30% glycerol, 600 mM dithiothreitol (DTT), 10% SDS and 0.2 mg ml^−1^ bromophenol blue) was mixed (1:1) with whole-cell extracts and boiled at 98 °C for 5 min. The analysis was carried out by SDS–PAGE using antibodies as indicated in the figure legends. Band intensities were quantified using the ImageJ software.

### Mammalian cell lysis and immunoblotting

For standard SDS–PAGE and immunoblotting experiments, cells from a well of a 12-well plate were lysed in 250 μl of ice-cold Triton lysis buffer (50 mM Tris pH 7.5, 1% Triton X-100, 150 mM NaCl, 50 mM NaF, 2 mM Na-vanadate and 0.011 g ml^−1^ β-glycerophosphate) supplemented with 1×PhosSTOP phosphatase inhibitors (cat. no. 4906837001, Roche) and 1×cOmplete protease inhibitors (cat. no. 11836153001, Roche) for 10 min on ice. The samples were clarified by centrifugation (20,000*g*, 15 min, 4 °C) and the supernatants were boiled in 1×SDS sample buffer (5×SDS sample buffer: 350 mM Tris–HCl pH 6.8, 30% glycerol, 600 mM DTT, 12.8% SDS and 0.12% bromophenol blue). The samples were analysed by SDS–PAGE using specific primary antibodies as indicated in the figures. The band intensities were quantified using the ImageJ software.

### Co-immunoprecipitation

For the yeast co-immunoprecipitations experiments, cells were collected by filtration and immediately frozen in liquid nitrogen. Subsequently, the pellets were mechanically disrupted in a FastPrep machine in 50-ml tubes containing 5 ml of ice-cold lysis buffer (50 mM Tris–HCl pH 7.5, 150 mM NaCl, 10% glycerol and 0.1% Nonidet P40) supplemented with 1×EDTA-free protease inhibitor cocktail (cat. no. 11697498001, Roche), 1×PhosSTOP phosphatase inhibitor and 4 ml glass beads. Total cell extracts were recovered from the beads and cleared by centrifugation. At this stage, samples were taken for input analysis and denatured with a Laemmli-based sample buffer (350 mM Tris–HCl pH 6.8, 30% glycerol, 600 mM DTT, 10% SDS and 0.2 mg ml^−1^ bromophenol blue). The cleared lysates (10–20 mg) were incubated for 2 h at 4 °C with magnetic beads pre-conjugated with anti-HA (cat. no. 88837, Pierce). The beads were then washed five times with high salt lysis buffer (50 mM Tris–HCl pH 7.5, 300 mM NaCl, 10% glycerol and 0.1% Nonidet P40) and eventually resuspended in 20 μl lysis buffer and 20 μl 2×Laemmli buffer. The samples were analysed by SDS–PAGE using specific primary antibodies as indicated in the figures.

For the mammalian endogenous protein co-immunoprecipitation experiments, cells of a near-confluent 10-cm dish were lysed in CHAPS IP buffer (50 mM Tris pH 7.5, 0.3% CHAPS detergent, 150 mM NaCl, 50 mM NaF, 2 mM Na-vanadate and 0.011 g ml^−1^ β-glycerophosphate) supplemented with 1×PhosSTOP phosphatase inhibitors and 1×EDTA-free cOmplete protease inhibitors (11873580001, Roche) for 10 min on ice. The samples were clarified by centrifugation (20,000*g*, 15 min, 4 °C) and the supernatants were subjected to immunoprecipitation by the addition of 3 μl of each antibody, incubation at 4 °C with rotation for 3 h, followed by incubation (4 °C with rotation) with 30 μl of a pre-washed Protein A agarose bead slurry (cat. no. 11134515001, Roche) for an additional hour. The beads were then washed four times with CHAPS IP wash buffer (50 mM Tris pH 7.5, 0.3% CHAPS detergent, 150 mM NaCl and 50 mM NaF) and boiled in 1×SDS loading buffer. A portion of the samples was kept aside as the input before the addition of antibodies. The samples were analysed by SDS–PAGE and the presence of co-immunoprecipitated proteins was detected by immunoblotting with the appropriate specific antibodies.

To test whether Mal-CoA influences mTORC1 complex stability/composition, 1 mM Mal-CoA lithium salt solution was added to the lysates before the immunoprecipitation of mTOR, as described earlier. For the mTORC1 IVK assays, endogenous mTOR was immunopurified from one 10-cm dish per condition, as described earlier. To test for malonylation of FASN and mTOR, endogenous proteins were immunopurified from one 10-cm dish per condition as described earlier, except that a high-stringency Triton IP lysis buffer (50 mM Tris pH 7.5, 1% Triton X-100, 500 mM NaCl, 50 mM NaF, 2 mM Na-vanadate, 0.011 g ml^−1^ β-glycerophosphate, 1×PhosSTOP phosphatase inhibitors and 1×EDTA-free cOmplete protease inhibitors) was used, and the samples were washed three times with Triton IP wash buffer (50 mM Tris pH 7.5, 1% Triton X-100, 500 mM NaCl and 50 mM NaF) and twice with Tris wash buffer (50 mM Tris pH 7.5) to remove the interacting proteins. Protein malonylation was assayed by immunoblotting using a Mal-K-specific antibody.

### Streptavidin pulldowns

For assays using SBP-tagged mTOR, HEK293FT cells were transfected with HA–Raptor and SBP–mTOR^WT^, SBP–mTOR^K1218R^ or SBP–mTOR^RR/AA^ (R2168A/R2170A) expression vectors, and complexes were purified by streptavidin pulldowns. Briefly, cells of a near-confluent well of a six-well plate were lysed in CHAPS IP buffer (50 mM Tris pH 7.5, 0.3% CHAPS detergent, 150 mM NaCl, 50 mM NaF, 2 mM Na-vanadate and 0.011 g ml^−1^ β-glycerophosphate) supplemented with 1×PhosSTOP phosphatase inhibitors and 1×EDTA-free cOmplete protease inhibitors for 10 min on ice, and the samples were clarified by centrifugation (20,000*g*, 15 min, 4 °C). Each supernatant was split in three equal fractions and each fraction was incubated (4 °C with rotation) with 20 μl of pre-washed Streptavidin Sepharose (cat. no. 90100484, Cytiva) for 1 h. The beads were then washed three times with CHAPS IP wash buffer (50 mM Tris pH 7.5, 0.3% CHAPS, 150 mM NaCl and 50 mM NaF) and either used for IVK assays (‘Mammalian mTORC1 kinase activity assays’ section) or boiled in 2×SDS loading buffer (5 min, 95 °C) and analysed by SDS–PAGE and immunoblotting to assess the stability of the mTOR complex.

### MST assays

The MST experiments were performed using a Monolith NT.115 (Nanotemper Technologies). Purification of TORC1 containing the N-terminally GFP-tagged Tor1 subunit was carried out as described previously^58^. For the protein–protein interaction experiments, GFP–Tor1-containing TORC1 (7.5 nM) was mixed with a series of twofold dilutions (a total of 16 samples) of Acc1 (highest concentration, 80 nM) or Fas1 (highest concentration, 20 nM) purified from yeast through TAP-tag immuno-enrichment with rabbit IgG-coated magnetic beads and subsequent TEV cleavage in elution buffer (50 mM HEPES NaOH pH 7.5 and 150 mM NaCl) for 1 h at 18 °C. The samples were loaded into Monolith NT.115 capillaries and MST measurements were performed using 80% laser power and medium MST power setting at 30 °C. For the small molecule–protein interaction experiments, GFP–Tor1-containing TORC1 (7.5 nM) was mixed with a series of twofold dilutions (a total of 16 samples) of Mal-CoA (M4263, Sigma-Aldrich; highest concentration, 5 mM) dissolved in 50 mM HEPES pH 7.5. The samples were loaded into Monolith NT.115 capillaries and MST measurements were performed using 80% laser power and medium MST power setting at 30 °C. The experiments were performed in triplicate and data were fitted using the *K*_d_ model of the MO.Affinity Analysis software (Nanotemper Technologies). The signal-to-noise ratio represents the response amplitude divided by the noise of the measurement. The *K*_d_ value was obtained by plotting the bound fraction against the log-transformed ligand concentration.

### TAG and palmitate (C16:0) measurements

The intracellular levels of TAGs and palmitate (C16:0) were determined using liquid chromatography-high resolution mass spectrometry-based analysis. Briefly, cells were seeded in 10-cm dishes and treated as described in the ‘Mammalian cell culture treatments’ section. The cells were scraped in medium and collected by centrifugation (400*g*, 3 min). The pellets were then washed three times with 1 ml ice-cold PBS, snap frozen in liquid nitrogen and kept at −80 °C until further processing.

For metabolite extraction, 1 ml of a −20 °C methyl-tert-butyl ether (MTBE):methanol:water (5:3:2, vol/vol/vol) mixture containing 0.2 µl deuterated EquiSplash lipidomix (Avanti); 0.1 µl U-^13^C^15^N amino-acid mix (Cambridge isotopes MSK_A2-1.2); 0.1 μl each of ^13^C_10_ ATP, ^15^N_5_ ADP and ^13^C_10_^15^N_5_ AMP (Sigma; all as 1 mg ml^−1^ stock solutions); and 0.2 μl deuterated citric acid (Sigma; 100 µg ml^−1^ stock solution) was added to each tube. The samples were vortexed for 10 s immediately after the addition of the extraction buffer and then incubated for 30 min on an orbital shaker at 4 °C. The proteins were pelleted by centrifugation (10 min, 21,000*g*, 4 °C) and the supernatants were transferred to a clean 2-ml tube. Separation of polar and lipid-containing phases was performed by the addition of 150 μl MTBE and 100 µl UPC/MS-grade water, brief vortexing, incubation for 15 min at 15 °C on an orbital shaker and centrifugation (10,000*g*, 5 min, 15 °C). The upper MTBE phase, which contains the hydrophobic metabolites (lipids), was then transferred to a new 1.5-ml microcentrifuge tube and the lipophilic extracts were immediately concentrated to complete dryness in a speed vacuum concentrator at room temperature. The dried samples were either stored at −80 °C or immediately processed for liquid chromatography–mass spectrometry analysis.

The lipid pellets were resuspended in 200 µl ultra-performance liquid chromatography (UPLC)-grade acetonitrile:isopropanol 70:30 (vol/vol). The samples were vortexed for 10 s, incubated for 10 min in a thermomixer at 4 °C and then centrifuged for 5 min at 10,000*g* and 4 °C. The cleared supernatants were transferred to 2-ml glass vials with 200 µl glass inserts (Chromatography Zubehör Trott). All samples were placed in an Acquity iClass UPLC (Waters) sample manager at 6 °C. The UPLC was connected to a Tribrid Orbitrap HRMS equipped with a heated electrospray ionization source (ID-X, Thermo Fisher Scientific). A 1-µl volume of each sample was injected into a 100 × 2.1 mm BEH C8 UPLC column packed with 1.7 µm particles (Waters). The flow rate of the UPLC was set to 400 ml min^−1^ and the buffer system consisted of buffer A (10 mM ammonium acetate and 0.1% acetic acid in UPLC-grade water) and buffer B (10 mM ammonium acetate and 0.1% acetic acid in UPLC-grade 70:30 (vol/vol) acetonitrile:isopropanol). The UPLC gradient was as follows: 0–1 min, 45% buffer A; 1–4 min, 45–25% buffer A; 4–12 min, 25–11% buffer A; 12–15 min, 11–1% buffer A; 15–20 min, 1% buffer A; 20–20.1 min, 1–45% buffer A; and 20.1–24 min, re-equilibration at 45% buffer A. This leads to a total runtime of 24 min per sample. The ID-X mass spectrometer was operated in positive-ionization mode for the first injection and negative-ionization mode for the second injection. In both cases, the analysed mass range was in the range of *m*/*z* 150–1,500. The resolution was set to 120,000, leading to approximately four scans per second. The RF lens was set to 60%, and the AGC target was set to 250%. The maximal ion time was set to 100 ms and the heated electrospray ionization source was operated with a spray voltage of 3.5 kV in positive-ionization mode and 3.2 kV in negative-ionization mode. The ion tube transfer capillary temperature was 300 °C, the sheath gas flow was 60 arbitrary units (a.u.), the auxiliary gas flow 20 a.u. and the sweep gas flow was set to 1 a.u. at 340 °C.

All samples were analysed in a randomized run order. Targeted data analysis was performed using the quan module of the TraceFinder 4.1 software (Thermo Fisher Scientific) in combination with a sample-specific in-house-generated compound database. For measurements of intracellular TAG abundance (Extended Data Fig. 5r), the sum of all values corresponding to the peak areas for the different individual TAGs was calculated for each sample.

### OPP incorporation assay

An OPP incorporation assay was used to test for de novo protein synthesis. Control HEK293T cells or cells treated with cerulenin (50 μM, 4 h) were incubated with 20 μM OPP reagent (NU-931-05, Jena Bioscience) for 30 min. The cells were subsequently washed with DPBS, trypsinized and fixed with ice-cold 70% ethanol for 30 min at −20 °C, followed by three washes with PBS containing 0.5% Tween-20. The incorporated OPP was then labelled with Alexa Fluor 488 picolyl azide using a Click-iT plus OPP protein synthesis assay kit (C10456, Thermo Fisher Scientific) as per the manufacturer’s instructions. The samples were run in a Guava easyCyte HT flow cytometer (Millipore) and analysed using FlowJo (v10). The cell population of interest was identified by plotting the forward scatter height versus the side scatter height, singlets were gated by plotting the forward scatter height versus the forward scatter area, and the median intensity of the Alexa Fluor 488 signal within the singlet population was used to quantify the extent of OPP incorporation.

### Production of recombinant His_6_-tagged 4E-BP1 protein in bacteria

Recombinant His_6_-tagged 4E-BP1 protein was produced by transforming *Escherichia coli* BL21 RP electrocompetent bacteria with the pETM-11-4E-BP1 vector described earlier according to standard procedures. Briefly, protein expression was induced with isopropyl-β-d-thiogalactopyranoside for 3 h at 30 °C, and His_6_–4E-BP1 was purified using Ni-NTA agarose (cat. no. 1018244, Qiagen) and eluted with 250 mM imidazole (A1073, Applichem).

### Yeast TORC1 kinase activity assays

TORC1 was purified from yeast cells and radioactive IVK assays were performed essentially as previously described^58^. Briefly, kinase reactions (total volume, 30 μl) were performed with 400 ng of purified His_6_–Lst4^Loop^ protein and 60 ng TORC1 in kinase buffer (50 mM HEPES NaOH pH 7.5 and 150 mM NaCl). The kinase reaction was pre-incubated with 2 μl of each compound from a 15× stock solution for 15 min to test the effect of Mal-CoA (M4263, Sigma-Aldrich), Ac-CoA (A2181, Sigma-Aldrich) and CoA (C3144, Sigma-Aldrich). Reactions were started by adding 2 μl of ATP mix (62.5 mM MgCl_2_, 4.5 mM ATP and 0.8 µM [γ-^32^P]ATP (SRP-501, Hartmann Analytic)). For kinase assays with different ATP concentrations, reactions were started by adding 2 µl of serial threefold dilutions of a more concentrated ATP mix (72 mM ATP and 0.8 µM [γ-^32^P]ATP), always containing 62.5 mM MgCl_2_. All reactions were carried out at 30 °C for 10 min and stopped with the addition of 3×sample buffer (50 mM Tris–HCl pH 6.8, 5% SDS, 0.05% bromophenol blue, 630 mM DTT and 30% glycerol) and heating at 65 °C for 10 min. Proteins were separated by SDS–PAGE and stained in-gel with SYPRO Ruby (S4942, Sigma-Aldrich) as the loading control. Substrate phosphorylation was analysed by autoradiography using a Typhoon FLA 9500 phosphorimager (GE Healthcare) and the raw density of the signals was quantified using the gel analysis tool of ImageJ.

### Yeast Snf1 kinase activity assay

The Snf1 complex was purified from a genomically C-terminally-tagged Snf1 yeast cultured in YPD and washed with water before filtration to induce Snf1 activation. After filtration, the cells were frozen in liquid nitrogen and cryogenically disrupted using a Precellys homogenizer in lysis buffer (50 mM Tris–HCl pH 7.5, 150 mM NaCl, 0.1% NP-40, 10% glycerol, 400 mM Pefabloc and Roche EDTA-free complete protease inhibitor). The cleared lysates were incubated with IgG-coupled Dynabeads (Dynabeads M-270 Epoxy; Invitrogen, Thermo Fisher Scientific) for 2 h at 4 °C. The beads were washed five times with lysis buffer and Snf1 was eluted through cleavage with TEV protease in elution buffer (50 mM Tris–HCl pH 7.5 and 0.5 mM EDTA) and stored at −80 °C after the addition of 10% glycerol. In vitro radioactive kinase reactions (total volume, 20 μl) were carried out in Snf1 kinase buffer (20 mM HEPES pH 7.5, 100 mM NaCl, 0.5 mM EDTA, 0.5 mM DTT and 5 mM magnesium acetate) with 60 ng Snf1 (quantified with respect to the Snf1 subunit) and 200 ng His_13_–Mig1^207–413^ (purified as described previously^95^). To test the effect of Mal-CoA (M4263, Sigma-Aldrich), the kinase reaction was pre-incubated with 2 μl of the compound from a 15× stock solution and threefold serial dilutions for 15 min. Reactions were started by adding 1.6 µl ATP mix (30% [γ-^32^P]-ATP (Hartmann Analytic, SRP-501), 60% 200 µM ATP and 1% kinase buffer (1×)); these were allowed to proceed for 10 min at 30 °C and then stopped by the addition of 3×sample buffer (50 mM Tris–HCl pH 6.8, 5% SDS, 0.05% bromophenol blue, 630 mM DTT and 30% glycerol) and heating to 65 °C for 10 min. Proteins were separated by SDS–PAGE and stained with SYPRO Ruby (S4942, Sigma-Aldrich) to assess loading. Substrate phosphorylation was analysed by autoradiography using a Typhoon FLA 9500 phosphorimager (GE Healthcare) and the raw density of the signals was quantified using the gel analysis tool of ImageJ.

### Mammalian mTORC1 kinase activity assays

In vitro mTORC1 kinase assays were developed based on previous reports^96,97^. Briefly, endogenous mTORC1 complexes were purified from HEK293FT cells essentially as described in the ‘Co-immunoprecipitation’ section, with minor modifications. Following the last wash step with CHAPS IP wash buffer, the beads were washed once with kinase wash buffer (25 mM HEPES pH 7.4 and 20 mM KCl) and excess liquid was removed using a Hamilton syringe—the final bead volume was approximately 10 μl. Kinase reactions were prepared by adding 10 μl of 3×kinase assay buffer (75 mM HEPES KOH pH 7.4, 60 mM KCl and 30 mM MgCl_2_) to the beads. To test the effect of Mal-CoA (M4263, Sigma-Aldrich), Ac-CoA (A2181, Sigma-Aldrich) and CoA (C3144, Sigma-Aldrich) on mTORC1 activity, 1 μl of each compound was pre-incubated with the kinase complex for 5 min before initiation of the reaction. The compound concentrations are indicated in the figures. Reactions were started by adding 10 μl kinase assay start buffer (25 mM HEPES KOH pH 7.4, 140 mM KCl and 10 mM MgCl_2_) supplemented with 500 μM ATP (final concentration in the reaction) and 35 ng recombinant His_6_–4E-BP1 substrate. The ATP concentrations used in the competition assays are described in the respective figures. The reactions were incubated at 30 °C for 30 min, and stopped by the addition of one volume of 2×SDS loading buffer and boiling (5 min, 95 °C). The samples were run in SDS–PAGE gels and the mTORC1-mediated phosphorylation on 4E-BP1^T37/46^ was detected by immunoblotting with a specific antibody (cat. no. 9459, Cell Signaling Technology). Signals were quantified using the gel analysis tool of ImageJ and are shown as the phospho-4E-BP1^T37/46^/4E-BP1 ratio.

For IVK experiments with SBP-tagged mTOR, HEK293FT cells were transfected with HA–Raptor and SBP–mTOR^WT^, SBP–mTOR^K1218R^ or SBP–mTOR^RR/AA^ (R2168A/R2170A) expression vectors, and complexes were purified by streptavidin pulldowns as described earlier, including one additional wash with kinase wash buffer (25 mM HEPES pH 7.4 and 20 mM KCl). In vitro activity assays were performed as described earlier for endogenous mTOR complexes.

### Mammalian Src kinase activity assay

In vitro assays for phosphorylation of Glo1 by Src were performed as described previously^98^. Briefly, bacterially purified His-tagged human Glo1 recombinant protein was mixed with 600 ng recombinant GST-tagged Src (0200-0000-1, ProQinase) in 1×kinase assay buffer (50 mM HEPES pH 7.4, 6 mM MgCl_2_, 6 mM β-glycerophosphate and 1 mM DTT) and 500 μM ATP in a total volume of 25 μl with the indicated concentrations of Mal-CoA, and incubated for 1 h at 30 °C. The phosphorylation reaction was stopped by the addition of 2×Laemmli buffer and boiling the samples for 5 min at 95 °C. The samples were run on SDS–PAGE gels and immunoblotted for Src-dependent phosphorylation of Glo1 at Y316 using a homemade antibody^98^.

### Immunofluorescence and confocal microscopy

Immunofluorescence and confocal microscopy experiments were performed as described previously^42,99^. Briefly, cells were seeded on glass coverslips (coated with fibronectin for the experiments with HEK293FT cells), treated or transfected as described in the figure legends, and fixed with 4% paraformaldehyde in 1×PBS (10 min, room temperature), followed by two permeabilization/washing steps with PBT (1×PBS and 0.1% Tween-20). The cells were blocked in BBT (1×PBS, 0.1% Tween-20 and 1% BSA) for 45 min. Staining with the primary antibodies anti-mTOR (cat. no. 2983, Cell Signaling Technology), anti-RagC (cat. no. 9480, Cell Signaling Technology) and anti-LAMP2 (H4B4, Developmental Studies Hybridoma Bank) diluted 1:200 in BBT solution was performed for 2 h, followed by three washes with PBT. Next, the cells were stained with highly cross-adsorbed fluorescent secondary antibodies (donkey anti-rabbit FITC and donkey anti-mouse TRITC; both from Jackson ImmunoResearch; diluted 1:200 in BBT) for 1 h. The nuclei were stained with DAPI (A1001, VWR; 1:1,000 in PBT) for 5 min and the coverslips were washed three times with PBT solution before mounting on glass slides with Fluoromount-G (00-4958-02, Invitrogen). All images were captured on an SP8 Leica confocal microscope (TCS SP8 X or TCS SP8 DLS, Leica Microsystems) using a ×40 oil objective lens. Image acquisition was performed using the LAS X software (Leica Microsystems).

### Quantification of co-localization

Co-localization analyses in the confocal microscopy experiments were performed as described previously^43,99^, using the Coloc2 plugin of the Fiji software^100^. A minimum of 12 representative images captured from two to four independent experiments were used per condition and the Manders’ co-localization coefficient with automatic Costes thresholding^101–103^ was calculated from individual cells. The exact number of cells used for the quantifications is provided in the respective figure legends. The area corresponding to the cell nucleus was excluded from the cell region of interest to prevent false-positive co-localization due to automatic signal adjustments. Manders’ co-localization coefficient is defined as a part of the signal of interest (mTOR or RagC) that overlaps with a second signal (LAMP2). Values are displayed as the mean ± s.e.m. and significance was calculated using a Student’s *t*-test (for pairwise comparisons) or one-way analysis of variance with post-hoc Holm–Sidak comparisons using GraphPad Prism.

### PLA assays

The proximity of endogenous FASN to lysosomes (LAMP2 as lysosomal marker) was assessed in MCF-7 cells using PLA assays, using the Duolink in situ red starter kit mouse/rabbit (DUO92101, Sigma-Aldrich) according to the manufacturer’s instructions, with minor modifications. To test the specificity of the FASN and LAMP2 PLA signal in the respective assays, transient knockdown of *FASN* or *LAMP2* was performed using a reverse transfection protocol with the appropriate siRNAs (siGENOME, Horizon Discoveries) and Lipofectamine RNAiMAX transfection reagent according to the manufacturer’s instructions. Cells were trypsinized 48 h post transfection, re-seeded in 16-well chamber slides (cat. no. 171080, Nunc Lab-Tek) and assayed approximately 24 h later. Briefly, the cells were fixed with 4% paraformaldehyde in 1×PBS, washed/permeabilized with PBT and blocked with Blocking solution from the Duolink kit. The samples were incubated overnight with the primary antibodies anti-FASN (PA5-22061, Thermo Fisher Scientific; dilution 1:400) and anti-LAMP2 (H4B4, Developmental Studies Hybridoma Bank; dilution 1:200) at 4 °C, processed according to the kit instructions and mounted on slides with a drop of DAPI-containing Duolink in situ mounting medium. Images were captured on a Leica TCS SP8 confocal microscope. A minimum of ten randomly chosen fields were acquired per condition as *z*-stacks, and the total PLA signal was calculated on the maximal projections of the single PLA channel using ImageJ. Data in graphs are presented as the average PLA area per cell, with *n*_siCtrl_ = 1,100, *n*_siFASN_ = 1,272 and *n*_siLAMP2_ = 1,311 individual cells. Statistical analysis was performed using GraphPad Prism.

### DexoMAG lysosome magnetic separation

The presence of various proteins in lysosomal fractions was tested using the DexoMAG method, performed essentially as described previously by others^54–56^. Briefly, cells were cultured to 70% confluence in a 10-cm dish and then incubated with 10% DexoMAG 40 (dextran-coated magnetite; Liquids Research Ltd), which was added directly to the culture medium for another 24 h. The cells were then washed twice with ice-cold PBS and scraped in 1 ml PBS_inh_ solution (1×PBS, 1×PhosSTOP phosphatase inhibitors and 1×cOmplete EDTA-free protease inhibitors and 500 μM sucrose (A2211, Applichem)). The cells were pelleted by centrifugation (100*g*, 3 min), resuspended in 1 ml PBS_inh_ solution, mechanically lysed with 20 strokes in pre-chilled 2 ml Dounce homogenizers, and the lysates were cleared by centrifugation (300*g*, 3 min). For the input samples, 50 μl of the cleared post-nuclear supernatant was diluted further by the addition of 150 μl PBS_inh_ and boiled in 2×SDS loading buffer. Another 700 μl of each post-nuclear supernatant sample was loaded on LS columns (130-042-401, Miltenyi Biotec) on a QuadroMACS 2 Tesla magnet (Miltenyi Biotec), which was pre-washed with 1 ml PBS containing 1% FA-free BSA (fraction V) and pre-equilibrated with 5 ml PBS_wash_ buffer (1×PBS and 100 μM sucrose). The flow-through was collected in clean tubes and 75 μl was transferred in a new tube, diluted further with 75 μl PBS_inh_ and boiled in 2×SDS loading buffer to be used as the lysosome-depleted fraction. The columns were washed three times with 5 ml PBS_wash_ and the lysosomal fractions were collected by detaching the column from the magnet and eluting the contents with 400 μl PBS_inh_ solution. The lysosome preparations (300 μl) were transferred to a new tube and boiled in 2×SDS loading buffer. The samples were analysed by SDS–PAGE and immunoblotted using the appropriate antibodies as indicated in the figure.

### Protein modelling

From the structure of human mTOR at 3.3 Å resolution (PDB ID 4JSP)^57^, missing residues (residues 1814–1867 and 2436–2492) were built by means of the Modeller version 10.0 (ref. ^104^), using the amino-acid sequences provided by the UniProt database (UniProtKB: P42345). Next, the kinase-domain region (residues 684–1058) was extracted and used for the docking calculation. The mTOR^R2168A/R2170A^ double mutant was modelled using UCSF Chimera (v1.5), using the Dynameomics rotamer library^105^, starting from the mTOR structure described above.

### Ligand modelling

Starting from the X-ray structure of human mTOR in a complex with ATPγS (PDB ID 4JSP), initial placement of the different ligands inside the binding pocket was performed. For ATP, the sulfur-to-oxygen substitution was performed manually. For Mal-CoA, its structure was extracted from PDB ID 5MY0 and subsequently aligned to the ATP structure in the mTOR catalytic pocket. Similar procedures were used for acetyl-CoA and CoA, and their coordinates were retrieved from PDB ID 1MZJ and 4L8A, respectively. All compounds were parameterized using the Ligand Reader and Modeller tool of the CHARMM-GUI software^106–108^.

### Docking simulations

For each compound, ten docking simulations were performed using the AutoDock software (version 4.2)^109^. Polar hydrogens and Kollman charges were added to the macromolecule^110^. Gasteiger charges were added to the ligands^111^ and, to confine the adenosine group in an ATP-like orientation, all rotatable bonds were blocked, except for the lateral chain. The grid dimension was adjusted to 54 × 40 × 40 points and the ligand–macromolecule interaction maps were computed using AutoGrid^112^. The automated docking software AutoDock Vina^113^ was used to calculate the binding affinity of ligands and the mTOR kinase domain. Docking energies were evaluated using empirical free energy functions and Lamarckian genetic algorithms^114^. A regular precision and a rigid ligand-docking were set for each docking run. To assess the stability of each docked pose, the energy values obtained by the docking were considered.

### Molecular dynamics simulations

For ATP, molecular dynamics simulations of the human mTOR kinase-domain region (residues 684–1058; PDB ID, 4JSP) were started after manual substitution of the sulfur atom in ATPγS in complex with two Mg^2+^ ions. For the other compounds (Mal-CoA, acetyl-CoA and CoA), the three most-favourable poses from the docking calculation were selected as the starting point for the molecular dynamics simulations. Each system was solvated with the TIP3P water model^115^ and neutralized with Na^+^ and Cl^−^ ions at physiological concentration (0.15 M). An energy minimization step was performed using the steepest descent algorithm. After the minimization, an NVT equilibration of 50 ps at 300 K was performed, using the V-rescale thermostat with a τT = 1 ps and an integration time step of 2 fs (ref. ^116^). Next, NPT simulation was run with a time step of 2 fs, using the Parrinello–Rahman barostat^117^, isotropic coupling and τp = 2 ps. The temperature was kept constant at 310 K. The electrostatic interactions were calculated using the particle mesh Ewald method^118^ with a cut-off of 1.2 nm. The same cut-off was applied for the van der Waals interactions. The simulations were performed with GROMACS (version 2020)^119^ and using the CHARMM-36 force field^120^. For each complex, we performed three replicate measurements lasting 400 ns, resulting in an aggregated time of 1.2 μs per system. Analysis of the interactions between the carboxylic group of Mal-CoA with R2168 (Fig. 7d) was carried out using the GROMACS tool gmx hbond. Videos were prepared using the Movie Maker tool of the VMD software (v1.9.3)^121^.

### Binding free energy calculation

The gmx_MMPBSA tool^122^ was used to estimate the binding free energy (Δ*G*_bind_) of the ligand–protein complexes. The molecular mechanics/generalized Born surface area method was used. Δ*G*_bind_ is defined as the difference between the free energy of the complex (*G*_COM_) and the free energy of the protein (*G*_P_) and ligand (*G*_L_), computed in solvent (equation 1):

1 $$\Delta {G}_{{\rm{bind}}}={G}_{{\rm{COM}}}-({G}_{{\rm{P}}}+{G}_{{\rm{L}}})$$

The free energy of each component is defined according to equation 2:

2 $$\langle {G}_{{\rm{x}}}\rangle =\langle {E}_{{\rm{MM}}}\rangle +\langle {G}_{{\rm{sol}}}\rangle -\langle {TS}\rangle$$

where *E*_MM_ is the molecular mechanics energy in the gas phase, *G*_sol_ is the free energy of solvation, *T* the absolute temperature and *S* the entropy.

For the molecular mechanics/generalized Born surface calculation, the trajectories were fitted along the simulation. The default method to compute non-polar solvation free energy was used, imposing a value of one^123^. According to the simulation settings, an ionic strength of 0.15 M was considered. A default value of four was applied for the ratio between the longest dimension of the rectangular finite-difference grid and that of the solute. The gmx_MMPSA_ana (v1.4.3) tool was used to analyse the results.

### Statistics and reproducibility

Statistical analyses and data presentation in graphs were performed using GraphPad Prism (versions 7.0.4, 8.0, 9.0 and 9.1.0). The data in the graphs in Fig. 6d,e and Extended Data Fig. 10a are the mean ± s.d. The data in all other graphs are the mean ± s.e.m. For the boxplots, the central line indicates the median, the box is the IQR (25th–75th percentile) and the whiskers indicate Q3 + 1.5 × IQR and Q1 − 1.5 × IQR. For graphs with only two conditions shown, significance for pairwise comparisons was calculated using a Student’s *t*-test (Figs. 2k,l and 3b,d and Extended Data Figs. 3, 4b,g,i, 5, 8f and 10i). For graphs with three or more conditions shown, significance for pairwise comparisons to the respective controls was calculated using a one-way analysis of variance with a post-hoc Holm–Sidak test (Figs. 2b,c,e,g,i, 3f–i, 4, 5 and 6o and Extended Data Figs. 1, 2, 4e,n, 6, 7 and 8b,d,h,j). Sample sizes (*n*) and significance values are indicated in the figures and figure legends (*P < 0.05; **P < 0.005; ***P < 0.0005; and NS, not significant).

All findings were reproducible over multiple independent experiments, within a reasonable degree of variability between replicates. The number of replicate experiments for each assay is provided in the respective figure legends. No statistical method was used to pre-determine sample size, which was determined in accordance with standard practices in the field. No data were excluded from the analyses. For metabolite measurements, samples were analysed in a randomized run order. The other experiments were not randomized, and the investigators were not blinded to allocation during experiments and outcome assessment.

### Reporting summary

Further information on research design is available in the Nature Portfolio Reporting Summary linked to this article.

## Online content

Any methods, additional references, Nature Portfolio reporting summaries, source data, extended data, supplementary information, acknowledgements, peer review information; details of author contributions and competing interests; and statements of data and code availability are available at https://doi.org/10.1038/s41556-023-01198-6.

## Supplementary information

Reporting Summary

Supplementary Video 1 Molecular dynamics simulation of ATP binding to mTOR.

Supplementary Video 2 Molecular dynamics simulation of Mal-CoA binding to mTOR.

Supplementary Video 3 Molecular dynamics simulation of CoA binding to mTOR.

Supplementary Video 4 Molecular dynamics simulation of acetyl-CoA binding to mTOR.

Supplementary Video 5 Molecular dynamics simulation of Mal-CoA binding to mTOR^R2168/2170A^.

Supplementary Tables 1–4 Supplementary Tables 1–4.

## Source data

Source Data Fig. 2 Uncropped blots for Fig. 2.

Source Data Fig. 3 Uncropped blots for Fig. 3.

Source Data Fig. 4 Uncropped blots for Fig. 4.

Source Data Fig. 5 Uncropped blots for Fig. 5.

Source Data Fig. 6 Uncropped blots for Fig. 6.

Source Data Fig. 8 Uncropped blots for Fig. 8.

Source Data Extended Data Fig. 1 Uncropped blots for Extended Data Fig. 1.

Source Data Extended Data Fig. 2 Uncropped blots for Extended Data Fig. 2.

Source Data Extended Data Fig. 3 Uncropped blots for Extended Data Fig. 3.

Source Data Extended Data Fig. 4 Uncropped blots for Extended Data Fig. 4.

Source Data Extended Data Fig. 5 Uncropped blots for Extended Data Fig. 5.

Source Data Extended Data Fig. 6 Uncropped blots for Extended Data Fig. 6.

Source Data Extended Data Fig. 7 Uncropped blots for Extended Data Fig. 7.

Source Data Extended Data Fig. 8 Uncropped blots for Extended Data Fig. 8.

Source Data Extended Data Fig. 10 Uncropped blots for Extended Data Fig. 10.

Source Data Fig. 1 Numerical source data for graphs in Fig. 1.

Source Data Fig. 2 Numerical source data for graphs in Fig. 2.

Source Data Fig. 3 Numerical source data for graphs in Fig. 3.

Source Data Fig. 4 Numerical source data for graphs in Fig. 4.

Source Data Fig. 5 Numerical source data for graphs in Fig. 5.

Source Data Fig. 6 Numerical source data for graphs in Fig. 6.

Source Data Fig. 7 Numerical source data for graphs in Fig. 7.

Source Data Fig. 8 Numerical source data for graphs in Fig. 8.

Source Data Extended Data Fig. 1 Numerical source data for graphs in Extended Data Fig. 1.

Source Data Extended Data Fig. 2 Numerical source data for graphs in Extended Data Fig. 2.

Source Data Extended Data Fig. 3 Numerical source data for graphs in Extended Data Fig. 3.

Source Data Extended Data Fig. 4 Numerical source data for graphs in Extended Data Fig. 4.

Source Data Extended Data Fig. 5 Numerical source data for graphs in Extended Data Fig. 5.

Source Data Extended Data Fig. 6 Numerical source data for graphs in Extended Data Fig. 6.

Source Data Extended Data Fig. 7 Numerical source data for graphs in Extended Data Fig. 7.

Source Data Extended Data Fig. 8 Numerical source data for graphs in Extended Data Fig. 8.

Source Data Extended Data Fig. 10 Numerical source data for graphs in Extended Data Fig. 10.

## Data Availability

Raw data from the lipid quantification analyses by mass spectrometry are available at the Zenodo repository (https://zenodo.org/record/8016427 and https://doi.org/10.5281/zenodo.8016427). The UniProt databases UniProtKB Q13085-1 and P42345, and PDB 4JSP, 5MY0, 1MZJ and 4L8A were used in this study. Source data are provided with this paper. All other data are available from the corresponding authors on reasonable request.
