# Supplementary material for: Malonyl-CoA is a conserved endogenous ATP-competitive mTORC1 inhibitor
Source: Nat Cell Biol. 2023 Aug 10;25(9):1303–18. doi: 10.1038/s41556-023-01198-6 (PMC10495264; doi:10.1038/s41556-023-01198-6)
Supplement: Supplementary file 1 — Reporting Summary [file 41556_2023_1198_MOESM1_ESM.pdf]

nature portfolio

Corresponding author(s): Drs Constantinos Demetriades, Claudio De Virgilio, Aurelio A. Teleman, Stefano Vanni

Last updated by author(s): Jun 15, 2023

Reporting Summary

Nature Portfolio wishes to improve the reproducibility of the work that we publish. This form provides structure for consistency and transparency in reporting. For further information on Nature Portfolio policies, see our Editorial Policies and the Editorial Policy Checklist.

Statistics

For all statistical analyses, confirm that the following items are present in the figure legend, table legend, main text, or Methods section.

n/a	Confirmed	
☐	☒	The exact sample size (*n*) for each experimental group/condition, given as a discrete number and unit of measurement
☐	☒	A statement on whether measurements were taken from distinct samples or whether the same sample was measured repeatedly
☐	☒	The statistical test(s) used AND whether they are one- or two-sided<br>*Only common tests should be described solely by name; describe more complex techniques in the Methods section.*
☒	☐	A description of all covariates tested
☐	☒	A description of any assumptions or corrections, such as tests of normality and adjustment for multiple comparisons
☐	☒	A full description of the statistical parameters including central tendency (e.g. means) or other basic estimates (e.g. regression coefficient) AND variation (e.g. standard deviation) or associated estimates of uncertainty (e.g. confidence intervals)
☐	☒	For null hypothesis testing, the test statistic (e.g. *F*, *t*, *r*) with confidence intervals, effect sizes, degrees of freedom and *P* value noted<br>*Give P values as exact values whenever suitable.*
☒	☐	For Bayesian analysis, information on the choice of priors and Markov chain Monte Carlo settings
☒	☐	For hierarchical and complex designs, identification of the appropriate level for tests and full reporting of outcomes
☒	☐	Estimates of effect sizes (e.g. Cohen's *d*, Pearson's *r*), indicating how they were calculated

Our web collection on statistics for biologists contains articles on many of the points above.

Software and code

Policy information about availability of computer code

Data collection

No code for data collection was generated in this study.

Commercial and non-commercial software packages from other developers used in this study are:
Confocal microscopy: Leica Application Suite X 3.5.7.23225; https://www.leica-microsystems.com/products/microscope-software/p/leica-las-x-ls/
Immunoblot intensity measurements: ImageJ (v1.52e, v1.53t); https://imagej.nih.gov/ij/download.html
Docking simulations: AUTODOCK (v. 4.2), https://autodock.scripps.edu
Molecular Dynamics simulations: GROMACS (v. 2020), https://www.gromacs.org
Molecular visualization and pictures : UCSF Chimera (v1.5); https://www.cgl.ucsf.edu/chimera/
Molecular visualization and videos: VMD (v1.9.3); http://www.ks.uiuc.edu/Research/vmd/

Data analysis

No code for data analysis was generated in this study.

Commercial and non-commercial software packages from other developers used in this study are:
Statistical analysis and graph preparation: GraphPad Prism (v. 7.0.4, 8.0, 9.0, and 9.1.0); https://www.graphpad.com/scientific-software/prism/
Flow cytometry analysis for OPP assay: FlowJo (v10); https://www.flowjo.com/solutions/flowjo/downloads
Quantification of colocalization: Fiji (Version 2.0.0-rc-68/v1.52e); https://imagej.net/software/fiji/
Lipidomics data analysis: TraceFinder (v4.1); https://www.thermofisher.com/de/en/home/industrial/mass-spectrometry/liquid-chromatography-mass-spectrometry-lc-ms/lc-ms-software/lc-ms-data-acquisition-software/tracefinder-software.html
Microscale thermophoresis data analyses: MO.Affinity Analysis software (v. 2.3); https://shop.nanotempertech.com/en/moaffinity-analysis-software-unlimited-licenses-34
Ligand parameterization and modeling for MD: CHARMM-GUI software, Ligand Reader & Modeller tool; https://www.charmm-gui.org/

Analysis of Molecular Dynamics trajectories: GROMACS (v. 2020), https://www.gromacs.org

For manuscripts utilizing custom algorithms or software that are central to the research but not yet described in published literature, software must be made available to editors and reviewers. We strongly encourage code deposition in a community repository (e.g. GitHub). See the Nature Portfolio guidelines for submitting code & software for further information.

Data

Policy information about availability of data

All manuscripts must include a data availability statement. This statement should provide the following information, where applicable:

- Accession codes, unique identifiers, or web links for publicly available datasets
- A description of any restrictions on data availability
- For clinical datasets or third party data, please ensure that the statement adheres to our policy

Raw data from lipid quantification analyses by mass spectrometry are available at the Zenodo repository (zenodo.org; DOI: 10.5281/zenodo.8016427). Source data (uncropped immunoblots, numerical data) are provided in Source Data. All other data are available from the corresponding authors upon reasonable request. UniProt databases UniProtKB: Q13085-1, P42345 and PDB data: 4JSP, 5MY0, 1MZJ, and 4L8A were used in this study.

Field-specific reporting

Please select the one below that is the best fit for your research. If you are not sure, read the appropriate sections before making your selection.

☒ Life sciences ☐ Behavioural & social sciences ☐ Ecological, evolutionary & environmental sciences

For a reference copy of the document with all sections, see nature.com/documents/nr-reporting-summary-flat.pdf

Life sciences study design

All studies must disclose on these points even when the disclosure is negative.

Sample size	No statistical methods were used for sample size determination, which was determined in accordance with standard practices in the field and based on our long-standing experience in this type of experimental approaches (e.g., PMIDs 26868506, 33497611, 33974911).<br>For colocalization analysis, individual cells from at least 12 independent representative fields were used per condition and pooled data from 3-4 independent experiments, or data from a representative experiment out of multiple replicates, are shown in dot plots. The number of individual cells quantified per condition in each panel is provided in the figure legends.
Data exclusions	No data were excluded from the analyses.
Replication	All findings were reproducible over multiple independent experiments, within a reasonable degree of variability between replicates. The exact numbers of replicate experiments are provided in the respective figure legends.
Randomization	For metabolite measurements, samples were analyzed in a randomized run-order. No sample randomization was performed for the other experiments described in this study, as the order of analysis does not influence the experimental outcomes.
Blinding	No blinding was included in the data collection or analysis, as the method of quantification over multiple replicates (for all experiments) and individual cells (for microscopy experiments) ensures unbiased processing.

Reporting for specific materials, systems and methods

We require information from authors about some types of materials, experimental systems and methods used in many studies. Here, indicate whether each material, system or method listed is relevant to your study. If you are not sure if a list item applies to your research, read the appropriate section before selecting a response.

Materials & experimental systems

n/a	Involved in the study	
☐	☒	Antibodies
☐	☒	Eukaryotic cell lines
☒	☐	Palaeontology and archaeology
☒	☐	Animals and other organisms
☒	☐	Human research participants
☒	☐	Clinical data
☒	☐	Dual use research of concern

Methods

n/a	Involved in the study	
☒	☐	ChIP-seq
☐	☒	Flow cytometry
☒	☐	MRI-based neuroimaging

March 2021

Antibodies

Antibodies used

A list of all primary antibodies used in this study is provided in Suppl. Table 2.
Information on Primary and Secondary antibodies used in this study is also provided below:

Primary antibodies

Yeast experiments
Mouse, monoclonal, anti-c-Myc, clone 9E10, sc-40, Santa Cruz Biotechnology, Lot #L1014
Mouse, monoclonal, anti-HA, clone 16B12, ENZ-ABS120, ENZO, Lot #10072011
Mouse, polyclonal, anti-GFP, 11814460001, Roche, Lot #11630500
Rabbit, polyclonal, anti-phospho-Sch9 (Thr737), home-made (De Virgilio lab)
Goat, polyclonal, anti-Sch9, home-made (De Virgilio lab)
Rabbit, polyclonal, anti-Adh1, 126745, Calbiochem, Lot #D29674
Mouse, monoclonal, anti-His6, H1029, Sigma-Aldrich, Lot #079M4822V
Rabbit, monoclonal, anti-phospho-AMPKα (Thr172), clone 40H9, #2535, Cell Signaling Technology, Lot #21
Goat, polyclonal, anti-Ypk1, Cell Signaling Technology (discontinued)
Rabbit, polyclonal, anti-phospho-Ypk1 (Thr662), home-made (Loewith lab)
Rabbit, monoclonal, anti-Malonyl-Lysine, #14942, Cell Signaling Technology, Lot #3

Mammalian cell experiments
Rabbit, monoclonal, anti-phospho-S6K (Thr389), clone 108D2, #9234, Cell Signaling Technology, Lot #1
Rabbit, polyclonal, anti-phospho-AKT (Ser473), #9271, Cell Signaling Technology, Lot #15
Rabbit, polyclonal, anti-AKT, #9272, Cell Signaling Technology, Lot #28
Rabbit, polyclonal, anti-S6K, #9202, Cell Signaling Technology, Lot #1
Rabbit, polyclonal, ant-phospho-4E-BP1 (Thr37/46), #9459, Cell Signaling Technology, Lot #10
Rabbit, polyclonal, anti-4E-BP1, #9452, Cell Signaling Technology, Lot #12
Rabbit, monoclonal, anti-mTOR, clone 7C10, #2983, Cell Signaling Technology, Lot #21
Rabbit, polyclonal, anti-FASN, 10624-2-AP, Proteintech, Lot # 100331
Rabbit, polyclonal, anti-ACC1, 21923-1-AP, Proteintech
Rabbit, monoclonal, anti-Malonyl-Lysine, #14942, Cell Signaling Technology, Lot #3
Rabbit, monoclonal, anti-RagA, clone D8B5, #4357, Cell Signaling Technology, Lot #3
Rabbit, monoclonal, anti-TSC1, clone D43E2, #6935, Cell Signaling Technology, Lot #4
Rabbit, polyclonal, anti-RAPTOR, A300-553A, Bethyl Laboratories, Lot #3
Rabbit, monoclonal, anti-GβL, clone 86B8, #3274, Cell Signaling Technology, Lot #4
Mouse, monoclonal, anti-FLAG, F1804, Sigma-Aldrich
Rabbit, monoclonal, anti-GAPDH, clone 14C10, #2118, Cell Signaling Technology, Lot #14
Mouse, monoclonal, anti-α-Tubulin, T9026, Sigma-Aldrich
Rabbit, monoclonal, anti-phospho-Acetyl-CoA-Carboxylase (Ser79), clone D7D11, #11818, Cell Signaling Technology, Lot #10
Rabbit, monoclonal, anti-phospho-AMPKα (Thr172), clone 40H9, #2535, Cell Signaling Technology, Lot #21
Rabbit, monoclonal, anti-p44/42 MAPK (Erk1/2), clone 137F5, #4695, Cell Signaling Technology, Lot #28
Rabbit, monoclonal, anti-phospho-p44/42 MAPK (Erk1/2) (Thr202/Tyr204), clone D13.14.4E, #4370, Cell Signaling Technology, Lot #28
Rabbit, monoclonal, anti-RagC, clone D8H5, #9480, Cell Signaling Technology, Lot #3
Rabbit, monoclonal, anti-Acetyl-CoA Carboxylase 2, clone D5B9, #8578, Cell Signaling Technology, Lot #3
Rabbit, monoclonal, anti-Rictor, clone D16H9, #9476, Cell Signaling Technology, Lot #4
Rabbit, polyclonal, anti-AMPKα, #2532, Cell Signaling Technology, Lot #21
Rabbit, monoclonal, anti-phospho-TFEB (Ser211), clone E9S8N, #37681, Cell Signaling Technology, Lot #2
Rabbit, polyclonal, anti-TFEB, #4240, Cell Signaling Technology, Lot #3
Rabbit, monoclonal, anti-phospho-GRB10 (Ser476), clone D4E6, #11817, Cell Signaling Technology, Lot #1
Rabbit, polyclonal, anti-GRB10, 23591-1-AP, Proteintech
Rabbit, polyclonal, anti-DEPDC5, ab185565, Abcam
Rabbit, polyclonal, anti-PSAP, 10801-1-AP, Proteintech
Rabbit, monoclonal, anti-ULK1, clone D8H5, #8054, Cell Signaling Technology, Lot #7
Rabbit, monoclonal, anti-phospho-ULK1 (Ser757), clone D7O6U, #14202, Cell Signaling Technology, Lot #5
Rabbit, polyclonal, anti-Cathepsin D, 21327-1-AP, Proteintech
Rat, monoclonal, anti-HA, clone 3F10, #11867423001, Roche, Lot #60789700
Mouse, monoclonal, anti-Actin, clone JLA20, Developmental Studies Hybridoma Bank
Mouse, monoclonal, anti-LAMP2, clone H4B4, Developmental Studies Hybridoma Bank
Mouse, monoclonal, anti-SBP-tag, clone 20, MAB10764, Sigma-Aldrich

Secondary antibodies

Yeast experiments
Peroxidase-conjugated goat anti-rabbit IgG (H+L), polyclonal, #1706515, BioRad
Peroxidase-conjugated goat anti-mouse IgG (H+L), polyclonal, #1706516, BioRad
Peroxidase-conjugated rabbit anti-goat IgG (H+L), polyclonal, #1721034, BioRad

Mammalian cell experiments
Peroxidase-conjugated AffiniPure donkey anti-rabbit IgG (H+L), polyclonal, #711-035-152, Jackson ImmunoResearch
Peroxidase-conjugated AffiniPure donkey anti-mouse IgG (H+L), polyclonal, #715-035-151, Jackson ImmunoResearch
Peroxidase-conjugated AffiniPure donkey anti-rat IgG (H+L), polyclonal, #712-035-153, Jackson ImmunoResearch

Rhodamine (TRITC)-conjugated AffiniPure donkey anti-mouse IgG (H+L), polyclonal, #715-025-150, Jackson ImmunoResearch
Fluorescein (FITC)-conjugated AffiniPure donkey anti-rabbit IgG (H+L), polyclonal, #711-095-152, Jackson ImmunoResearch

Validation

Specificity of phospho-antibodies extensively verified in this study and in the context of other projects in the Demetriades, Teleman and De Virgilio/Loewith labs, using chemical inhibitors, starvation media, or mutants altering the activity of the respective kinases. Specificity of total protein antibodies extensively verified in this study and in the context of other projects in the Demetriades Teleman and De Virgilio/Loewith labs, using knock-out cell lines, knock-down and overexpression experiments, or using yeast strains deleted for the respective gene.

Additional information for all commercially-available antibodies used in this study (Suppl. Table S2) can be found in the manufacturer's website for each product:

Yeast experiments

anti-c-Myc, sc-40, Santa Cruz Biotechnology, validated for Western Blotting, Immunoprecipitation, Immunohistochemistry, Immunofluorescence, Flow Cytometry and ELISA, relevant citations can be found on the manufacturer's website (https://www.scbt.com/p/c-myc-antibody-9e10)

anti-HA, ENZ-ABS120, ENZO, validated for Western Blotting, Immunoprecipitation, Immunohistochemistry and Immunofluorescence, relevant citations can be found on the manufacturer's website (https://www.enzolifesciences.com/ENZ-ABS120/ha.11-monoclonal-antibody-16b12-purified)

anti-GFP, 11814460001, Roche, validated for Western Blotting, Immunoprecipitation, Immunostaining and Immunofluorescence, relevant citations can be found on the manufacturer's website (https://www.sigmaaldrich.com/CH/en/product/roche/11814460001)

anti-Adh1, 126745, Calbiochem, validated for Western Blotting and ELISA, relevant information can be found in the Merck catalog (https://www.merckmillipore.com/Web-TW-Site/zh_TW/-/TWD/ShowDocument-File?ProductSKU=EMD_BIO-OP20&DocumentId=200904.3114.ProNet&DocumentUID=13258&DocumentType=BRO&Language=EN&Country=NF&Origin=PDP)

anti-His6, H1029, Sigma-Aldrich, validated for Western Blotting, Immunoprecipitation and ELISA, relevant citations can be found on the manufacturer's website (https://www.sigmaaldrich.com/CH/en/product/sigma/h1029)

Mammalian cell experiments

anti-phospho-S6K (Thr389), #9234, Cell Signaling Technology, validated for Western Blotting, relevant citations can be found on the manufacturer's website (https://www.cellsignal.com/products/primary-antibodies/phospho-p70-s6-kinase-thr389-108d2-rabbit-mab/9234)

anti-S6K, #9202, Cell Signaling Technology, validated for Western Blotting and Immunoprecipitation, relevant citations can be found on the manufacturer's website (https://www.cellsignal.com/products/primary-antibodies/p70-s6-kinase-antibody/9202)

anti-phospho-AKT (ser473), #9271, Cell Signaling Technology, validated for Western Blotting, Immunoprecipitation, Immunofluorescence, and Flow Cytometry, relevant citations can be found on the manufacturer's website (https://www.cellsignal.com/products/primary-antibodies/phospho-akt-ser473-antibody/9271)

anti-AKT, #9272, Cell Signaling Technology, validated for Western Blotting, Immunoprecipitation, Immunofluorescence, and Flow Cytometry, relevant citations can be found on the manufacturer's website (https://www.cellsignal.com/products/primary-antibodies/akt-antibody/9272)

anti-phospho-4E-BP1 (Thr37/46), #9459, Cell Signaling Technology, validated for Western Blotting, relevant citations can be found on the manufacturer's website (https://www.cellsignal.com/products/primary-antibodies/phospho-4e-bp1-thr37-46-antibody/9459)

anti-4E-BP1, #9452, Cell Signaling Technology, validated for Western Blotting, relevant citations can be found on the manufacture's website (https://www.cellsignal.com/products/primary-antibodies/4e-bp1-antibody/9452)

anti-mTOR, #2983, Cell Signaling Technology, validated for Western Blotting, Immunohistochemistry, Immunofluorescence and Flow Cytometry, relevant citations can be found on the manufacturer's website (https://www.cellsignal.com/products/primary-antibodies/mtor-7c10-rabbit-mab/2983)

anti-FASN, 10624-2-AP, Proteintech, validated for Western Blotting, Immunoprecipitation, Immunohistochemistry, Immunofluorescence, and Flow Cytometry, relevant citations can be found on the manufacturer's website (https://www.ptglab.com/products/FASN-Antibody-10624-2-AP.htm)

anti-ACC1, 21923-1-AP, Proteintech, validated for Western Blotting, Immunoprecipitation, Immunohistochemistry, and Immunofluorescence, relevant citations can be found on the manufacturer's website (https://www.ptglab.com/products/ACACA-Antibody-21923-1-AP.htm)

anti-Malonyl-Lysine, #14942, Cell Signaling Technology, validated for Western Blotting, relevant citations can be found on the manufacturer's website (https://www.cellsignal.com/products/proteomic-analysis-products/malonyl-lysine-mal-k-multimab-rabbit-mab-mix/14942)

anti-TSC1, #6935, Cell Signaling Technology, validated for Western Blotting, and Immunoprecipitation, relevant citations can be found on the manufacturer's website (https://www.cellsignal.com/products/primary-antibodies/hamartin-tsc1-d43e2-rabbit-mab/6935)

anti-RAPTOR, #A300-553A, Bethyl Laboratories, validated for Western Blotting, and Immunoprecipitation, relevant citations can be found on the manufacturer's website (https://www.fortislife.com/products/primary-antibodies/rabbit-anti-raptor-antibody/BETHYL-A300-553)

anti-GβL, #3274, Cell Signaling Technology, validated for Western Blotting, and Immunoprecipitation, relevant citations can be found on the manufacturer's website (https://www.cellsignal.com/products/primary-antibodies/gbl-86b8-rabbit-mab/3274)

anti-FLAG, F1804, Sigma-Aldrich, validated for Western Blotting, Immunoprecipitation, Immunohistochemistry, Immunofluorescence, and Immunocytochemistry, relevant citations can be found on the manufacturer's website (https://www.sigmaaldrich.com/DE/en/product/sigma/f1804)

anti-GAPDH, #2118, Cell Signaling Technology, validated for Western Blotting, Immunohistochemistry, Immunofluorescence, and Flow Cytometry, relevant citations can be found on the manufacturer's website (https://www.cellsignal.com/products/primary-antibodies/gapdh-14c10-rabbit-mab/2118)

anti-α-Tubulin, T9026, Sigma-Aldrich, validated for Western Blotting, Immunofluorescence, and Immunohistochemistry, relevant citations can be found on the manufacturer's website (https://www.sigmaaldrich.com/DE/en/product/sigma/t9026)

anti-phospho-Acetyl-CoA Carboxylase (Ser79), #11818, Cell Signaling Technology, validated for Western Blotting, Immunoprecipitation, Immunohistochemistry, and Immunofluorescence, relevant citations can be found on the manufacturer's website (https://www.cellsignal.com/products/primary-antibodies/phospho-acetyl-coa-carboxylase-ser79-d7d11-rabbit-mab/11818)

anti-phospho-AMPKα (Thr172), #2535, Cell Signaling Technology, validated for Western Blotting, Immunoprecipitation, and Immunohistochemistry, relevant citations can be found on the manufacturer's website (https://www.cellsignal.com/products/primary-antibodies/phospho-ampka-thr172-40h9-rabbit-mab/2535)

anti-p44/42 MAPK (Erk1/2), #4695, Cell Signaling Technology, validated for Western Blotting, Immunoprecipitation, Immunohistochemistry, Immunofluorescence, and Flow Cytometry, relevant information can be found on the manufacturer's website https://www.cellsignal.com/products/primary-antibodies/p44-42-mapk-erk1-2-137f5-rabbit-mab/4695

anti-phospho-p44/42 MAPK (Erk1/2) (Thr202/Tyr204), Cell Signaling technology, validated for Western Blotting, Immunoprecipitation, Immunohistochemistry, Immunofluorescence, and Flow Cytometry, relevant information can be found on the manufacturer's website (https://www.cellsignal.com/products/primary-antibodies/phospho-p44-42-mapk-erk1-2-thr202-tyr204-d13-14-4e-xp-rabbit-mab/4370)

anti-RagC, #9480, Cell Signaling Technology, validated for Western Blotting, Immunoprecipitation, Immunofluorescence and Flow Cytometry, relevant citations can be found on the manufacturer's website (https://www.cellsignal.com/products/primary-antibodies/ragc-d8h5-rabbit-mab/9480)

anti-Acetyl-CoA Carboxylase 2, #8578, Cell Signaling Technology, validated for Western Blotting and Immunoprecipitation, relevant citations can be found on the manufacturer's website (https://www.cellsignal.com/products/primary-antibodies/acetyl-coa-carboxylase-2-d5b9-rabbit-mab/8578)

anti-Rictor, #9476, Cell Signaling Technology, validated for Western Blotting, relevant citations can be found on the manufacturer's website (https://www.cellsignal.com/products/primary-antibodies/rictor-d16h9-rabbit-mab/9476)

anti-AMPKα, #2532, Cell Signaling Technology, validated for Western Blotting and Immunoprecipitation, relevant citations can be found on the manufacturer's website (https://www.cellsignal.com/products/primary-antibodies/ampka-antibody/2532)

anti-phospho-TFEB (Ser211), #37681, Cell Signaling Technology, validated for Western Blotting, relevant citations can be found on the manufacturer's website (https://www.cellsignal.com/products/primary-antibodies/phospho-tfeb-ser211-e9s8n-rabbit-mab/37681)

anti-TFEB, #4240, Cell Signaling Technology, validated for Western Blotting and Immunoprecipitation, relevant citations can be found on the manufacturer's website (https://www.cellsignal.com/products/primary-antibodies/tfeb-antibody/4240)

anti-phospho-GRB10 (Ser476), #11817, Cell Signaling Technology, validated for Western Blotting and Immunoprecipitation, relevant citations can be found on the manufacturer's website (https://www.cellsignal.com/products/primary-antibodies/phospho-grb10-ser476-d4e6-rabbit-mab/11817)

anti-GRB10, 23591-1-AP, Proteintech, validated for Western Blotting, Immunohistochemistry, and Immunofluorescence, relevant citations can be found on the manufacturer's website (https://www.ptglab.com/products/GRB10-Antibody-23591-1-AP.htm)

anti-DEPDC5, ab185565, Abcam, validated for Immunocytochemistry and Immunofluorescence, relevant citations can be found on the manufacturer's website (https://www.abcam.com/products/primary-antibodies/depdc5-antibody-ab185565.html)

anti-PSAP, 10801-1-AP, Proteintech, validated for Western Blotting, Immunohistochemistry, and Immunocytochemistry, relevant citations can be found on the manufacturer's website (https://www.ptglab.com/products/PSAP-Antibody-10801-1-AP.htm)

anti-ULK1, #8054, Cell Signaling Technology, validated for Western Blotting, relevant citations can be found on the manufacture's website
(https://www.cellsignal.com/products/primary-antibodies/ulk1-d8h5-rabbit-mab/8054)

anti-phospho-ULK1 (Ser757), #14202, Cell Signaling Technology, validated for Western Blotting, relevant citations can be found on the manufacturer's website
(https://www.cellsignal.com/products/primary-antibodies/phospho-ulk1-ser757-d7o6u-rabbit-mab/14202)

anti-Cathepsin D, 21327-1-AP, Proteintech, validated for Western Blotting and Immunohistochemistry, relevant citations can be found on the manufacturer's website (https://www.ptglab.com/products/CTSD-Antibody-21327-1-AP.htm)

anti-HA, #11867423001, Roche, validated for Western Blotting, Dot Blots, ELISA, Immunocytochemistry, and Immunoprecipitation, relevant citations can be found on the manufacturer's website (https://www.sigmaaldrich.com/DE/en/product/roche/roahaha)

anti-RagA, #4357, Cell Signaling Technology, validated for Western Blotting and Immunoprecipitation, relevant citations can be found on the manufacturer's website (https://www.cellsignal.com/products/primary-antibodies/raga-d8b5-rabbit-mab/4357)

anti-Actin, Developmental Studies Hybridoma Bank, validated for Western Blotting and Immunofluorescence, relevant citations can be found on the manufacturer's website (https://dshb.biology.uiowa.edu/JLA20)

anti-LAMP2, Developmental Studies Hybridoma Bank, validated for Western Blotting, FACS, Function Blocking, Immunofluorescence, Immunohistochemistry and Immunoprecipitation, relevant citations can be found on the manufacturer's website (https://dshb.biology.uiowa.edu/H4B4)

anti-SBP-tag, MAB10764, Sigma-Aldrich, validated for Western Blotting and Immunocytochemistry, relevant citations can be found on the manufacturer's website https://www.merckmillipore.com/DE/en/product/Anti-SBP-tag-Antibody-clone-20-(MAB10764,MM_NF-MAB10764?ReferrerURL=https%3A%2F%2Fwww.google.com%2F)

Eukaryotic cell lines

Policy information about cell lines

Cell line source(s)	HEK293FT cells were purchased from Invitrogen. Wild-type immortalized MEFs were a kind gift of Kun-Liang Guan (described in PMID: 25567907). U2OS cells were a kind gift of Nils-Göran Larsson (originally obtained from ATCC, #HTB-96). The other cells lines, including WI-26, MCF-7, and HEK293T (ATCC, #CRL-3216), are part of the Demetriades and Teleman lab collections, also originating from the lab of Michael Boutros (DKFZ, Heidelberg).
Authentication	The identity of the WI-26 cells was validated using the Short Tandem Repeat (STR) profiling service, provided by Multiplexion GmbH. The identity of the HEK293FT and MCF-7 cells was validated by the Multiplex human Cell Line Authentication test (Multiplexion GmbH), which uses a single nucleotide polymorphism (SNP) typing approach, and was performed as described at www.multiplexion.de. The other cell lines used in this study (U2OS, HEK293T) have not been authenticated.
Mycoplasma contamination	All cell lines were regularly tested for Mycoplasma contamination, using a PCR-based approach and were confirmed to be Mycoplasma-free.
Commonly misidentified lines (See ICLAC register)	No commonly misidentified cell lines were used in this study.

Flow Cytometry

Plots

Confirm that:

☒ The axis labels state the marker and fluorochrome used (e.g. CD4-FITC).

☒ The axis scales are clearly visible. Include numbers along axes only for bottom left plot of group (a 'group' is an analysis of identical markers).

☒ All plots are contour plots with outliers or pseudocolor plots.

☒ A numerical value for number of cells or percentage (with statistics) is provided.

Methodology

Sample preparation	20 µM OPP reagent (Jena Bioscience #NU-931-05) was added for 30 minutes to the cells. The cells were subsequently washed with DPBS, trypsinized, and fixed with ice-cold 70% ethanol for 30 minutes at -20C degrees, followed by three washes in PBS supplemented with 0.5% Tween-20. The incorporated OPP was then labeled with the Alexa488 Fluor Picolyl azide using the Click-iT Plus OPP Protein synthesis assay kit (Life Technologies #C10456), as per manufacturer's instructions.
Instrument	Guava easyCyte HT flow cytometer (Millipore)
Software	FlowJo (v10)
Cell population abundance	47.9% (95.7% singlets)
Gating strategy	The cell population of interest was identified plotting FSC-H vs SSC-H, singlets gated by plotting FSC-H vs FSC-A, and the mean intensity of the Alexa488 signal within the singlets population was used to quantify the extent of OPP incorporation.

☒ Tick this box to confirm that a figure exemplifying the gating strategy is provided in the Supplementary Information.
