# Supplementary material for: Malonyl-CoA is a conserved endogenous ATP-competitive mTORC1 inhibitor
Source: Nat Cell Biol. 2023 Aug 10;25(9):1303–18. doi: 10.1038/s41556-023-01198-6 (PMC10495264; doi:10.1038/s41556-023-01198-6)

Uncropped blots for Fig. 4a

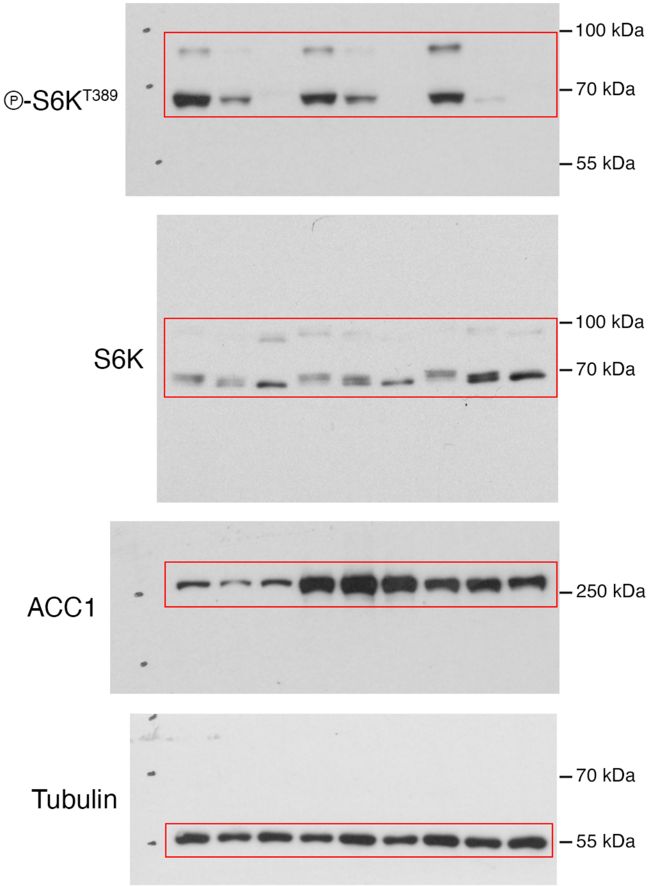

Uncropped blots for Fig. 4c

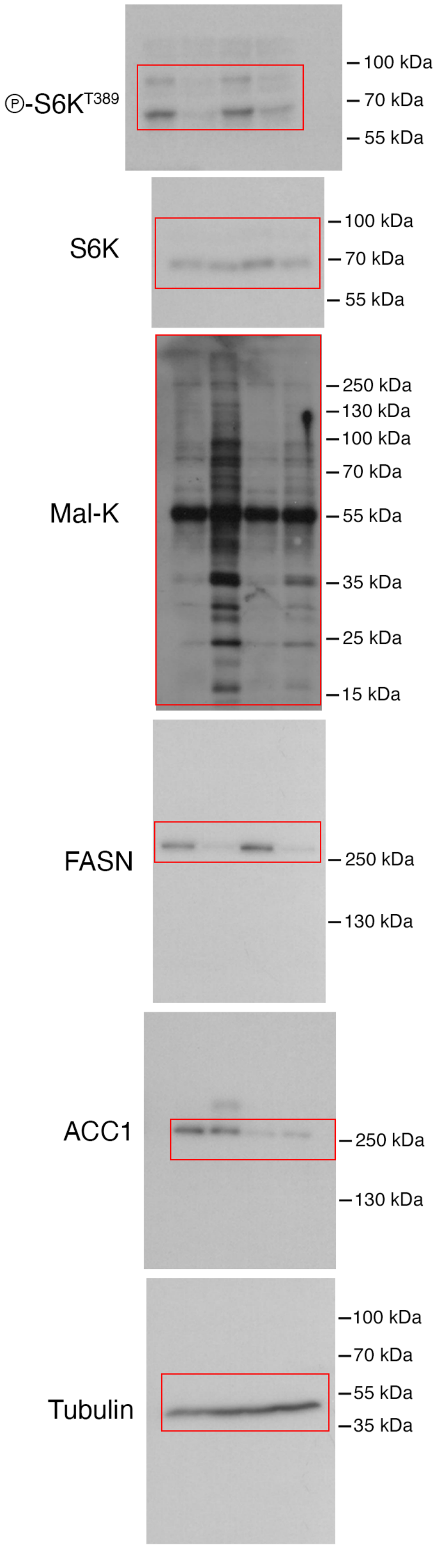

Uncropped blots for Fig. 4f

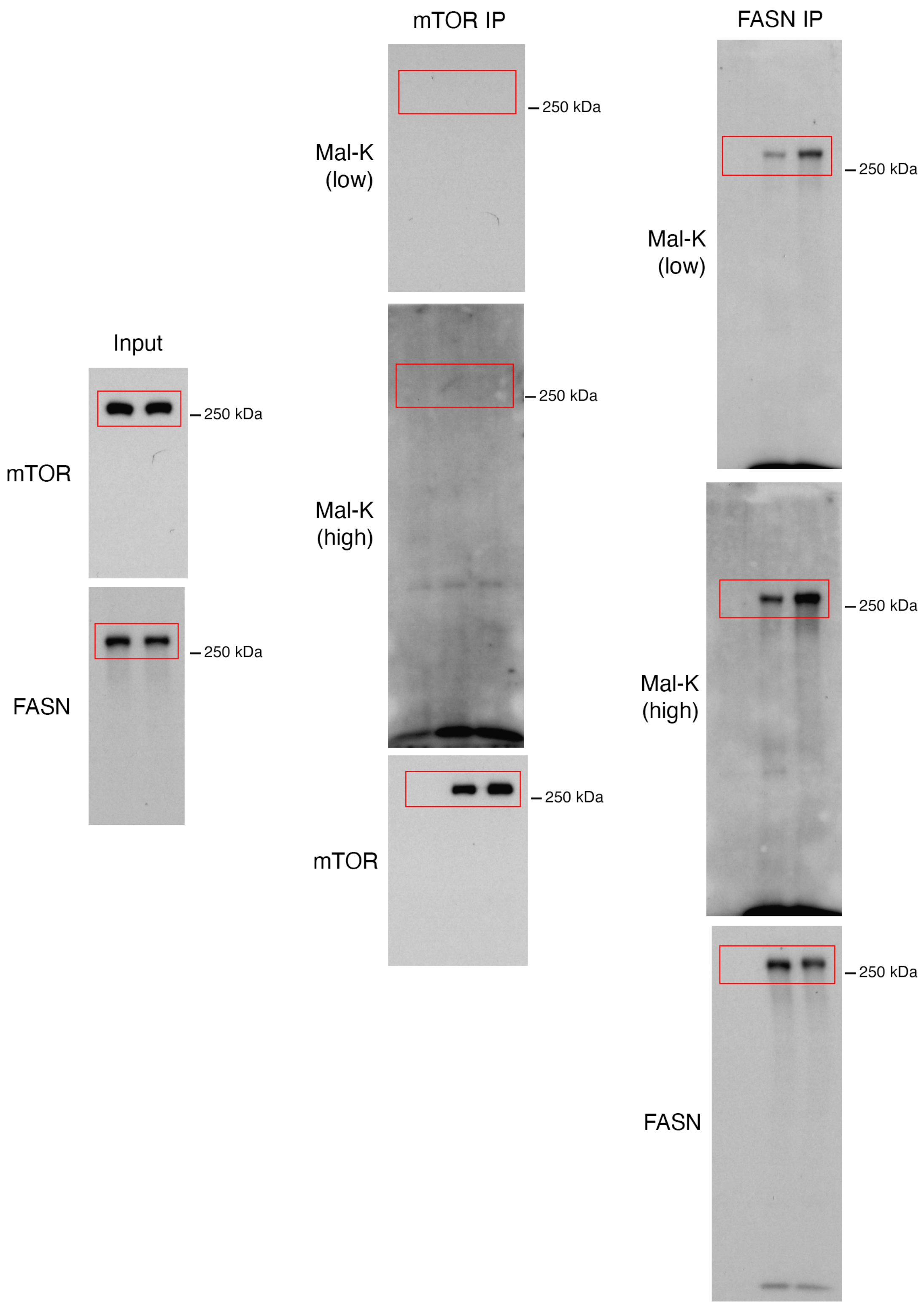

Uncropped blots for Fig. 4g

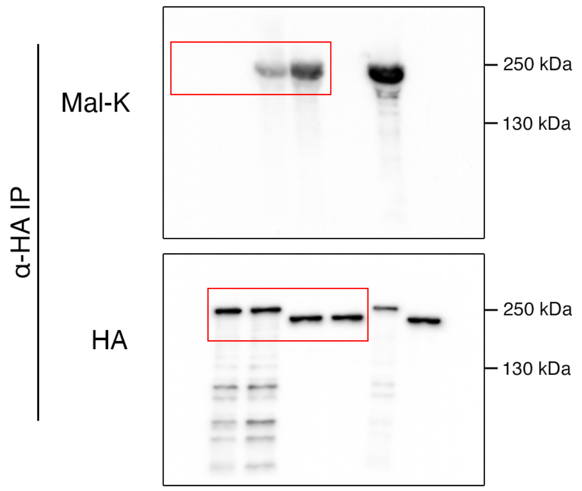

Supplement: Source Data Fig. 4 — Uncropped blots for Fig. 4. [file 41556_2023_1198_MOESM10_ESM.pdf]
