# Supplementary material for: Malonyl-CoA is a conserved endogenous ATP-competitive mTORC1 inhibitor
Source: Nat Cell Biol. 2023 Aug 10;25(9):1303–18. doi: 10.1038/s41556-023-01198-6 (PMC10495264; doi:10.1038/s41556-023-01198-6)

Uncropped blots for Fig. 8a

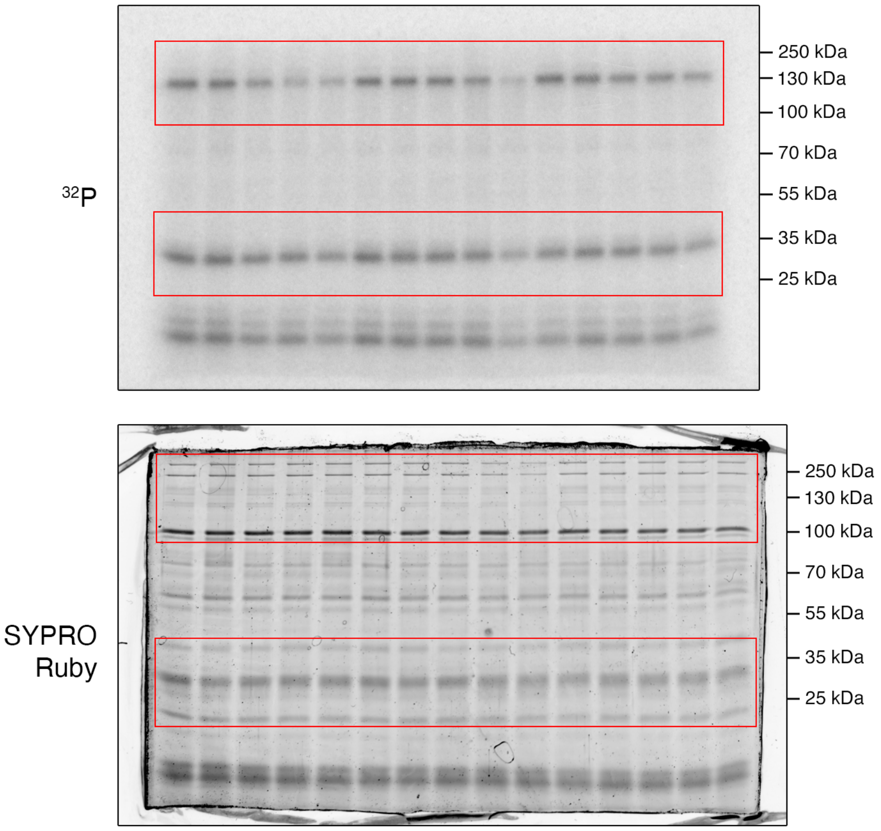

Uncropped blots for Fig. 8c

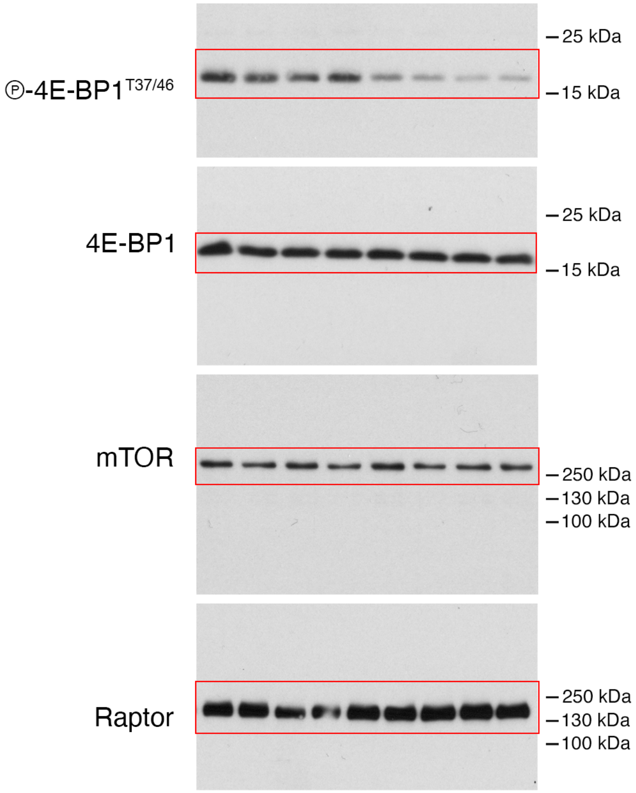

Uncropped blots for Fig. 8d

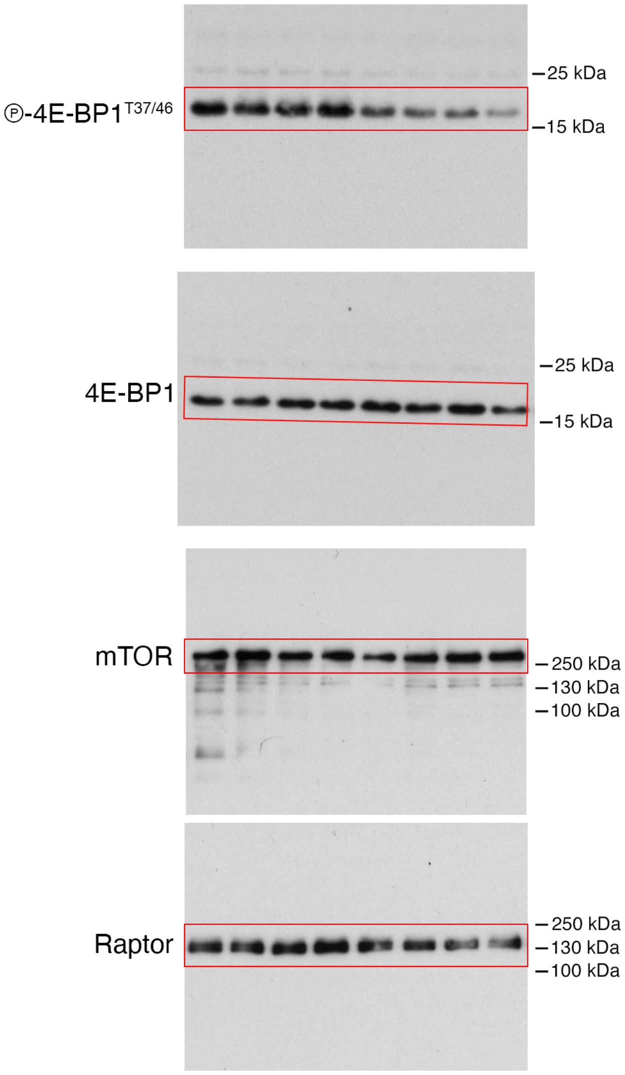

Uncropped blots for Fig. 8e

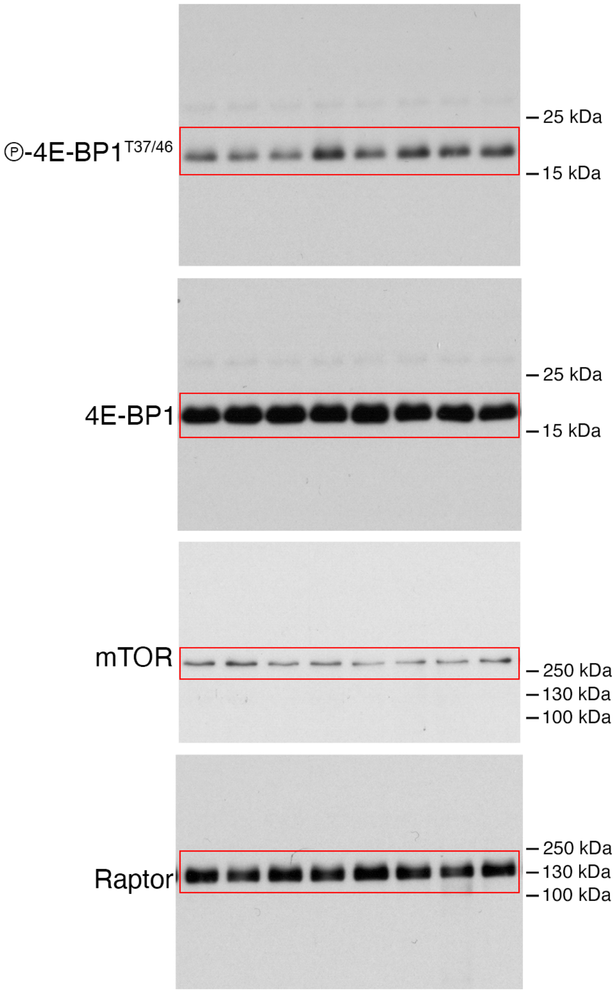

Uncropped blots for Fig. 8g

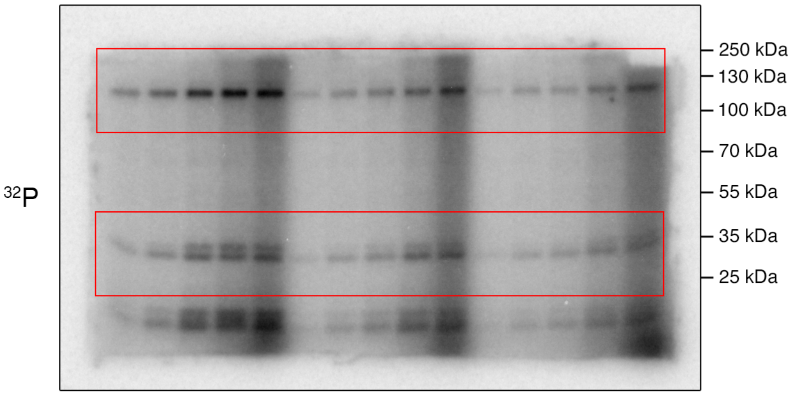

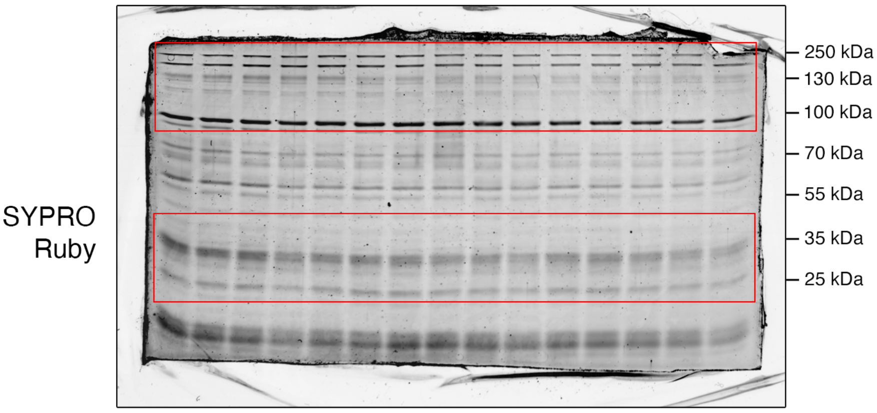

Uncropped blots for Fig. 8i

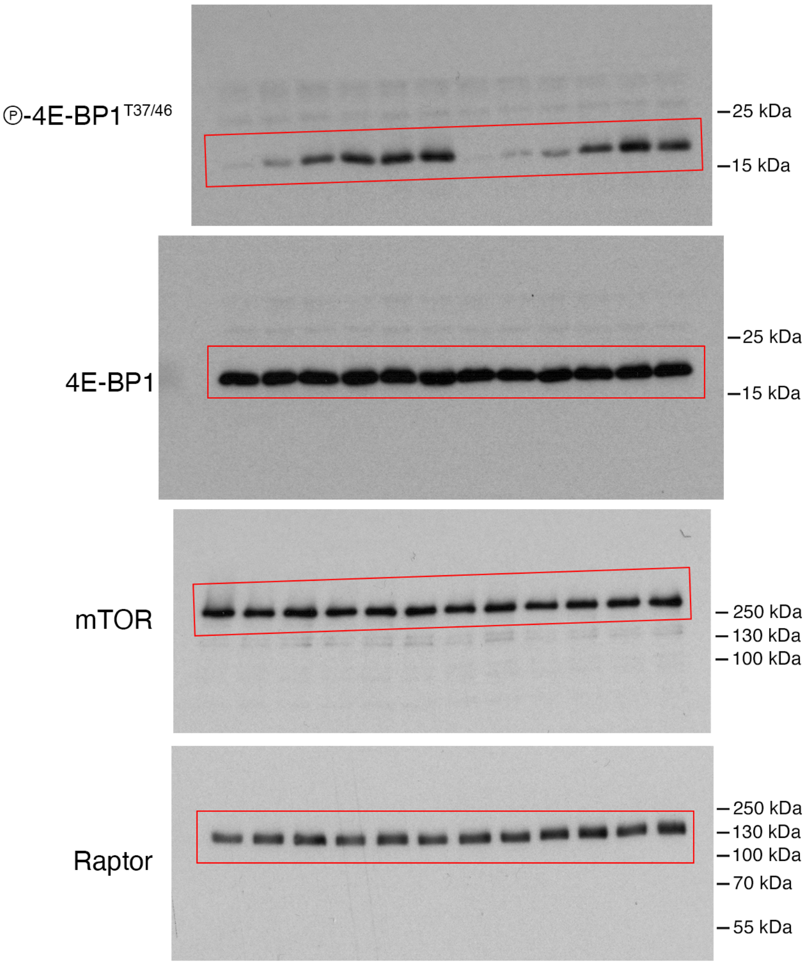

Supplement: Source Data Fig. 8 — Uncropped blots for Fig. 8. [file 41556_2023_1198_MOESM13_ESM.pdf]
