# Supplementary figures and images for: Malonyl-CoA is a conserved endogenous ATP-competitive mTORC1 inhibitor
Source: Nat Cell Biol. 2023 Aug 10;25(9):1303–18. doi: 10.1038/s41556-023-01198-6 (PMC10495264; doi:10.1038/s41556-023-01198-6)

**Uncropped blots for Extended Data Fig. 1a**

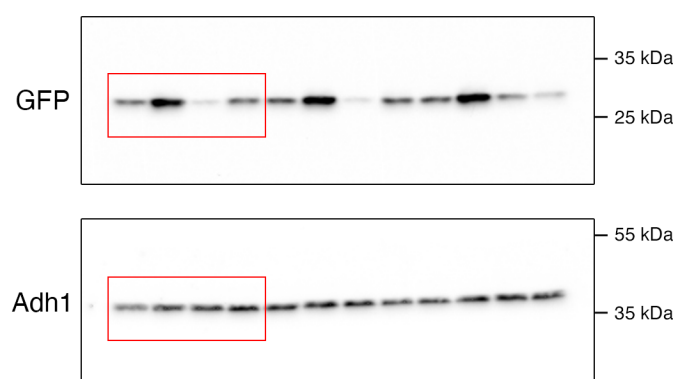

Uncropped blots for Extended Data Fig. 1f

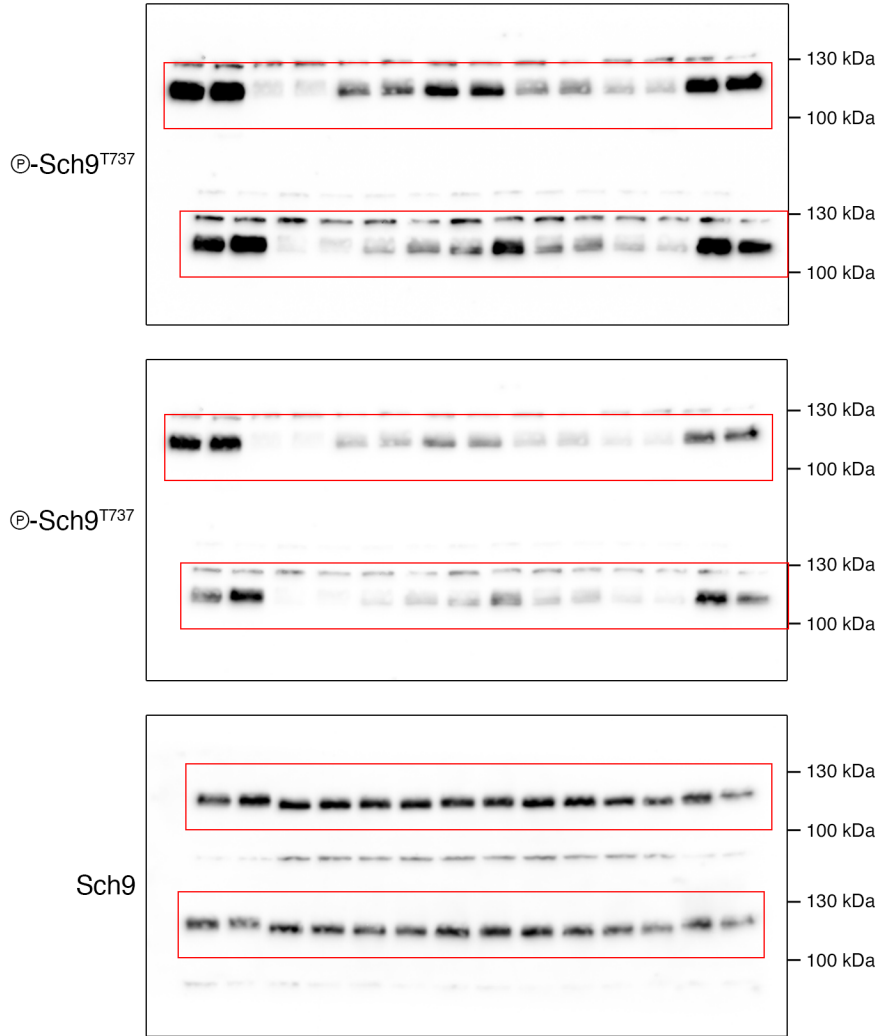

Supplement: Source Data Extended Data Fig. 1 — Uncropped blots for Extended Data Fig. 1. [file 41556_2023_1198_MOESM14_ESM.pdf]

## Uncropped blots for Extended Data Fig. 2a

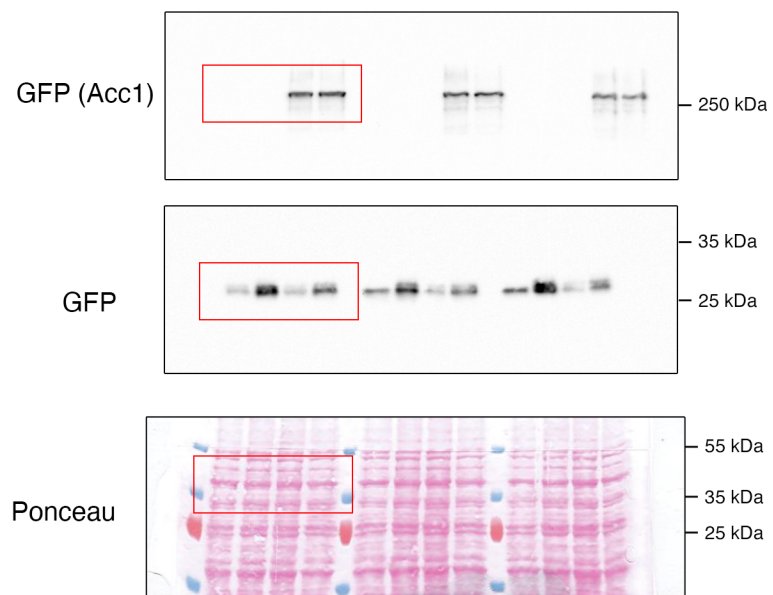

Uncropped blots for Extended Data Fig. 2d

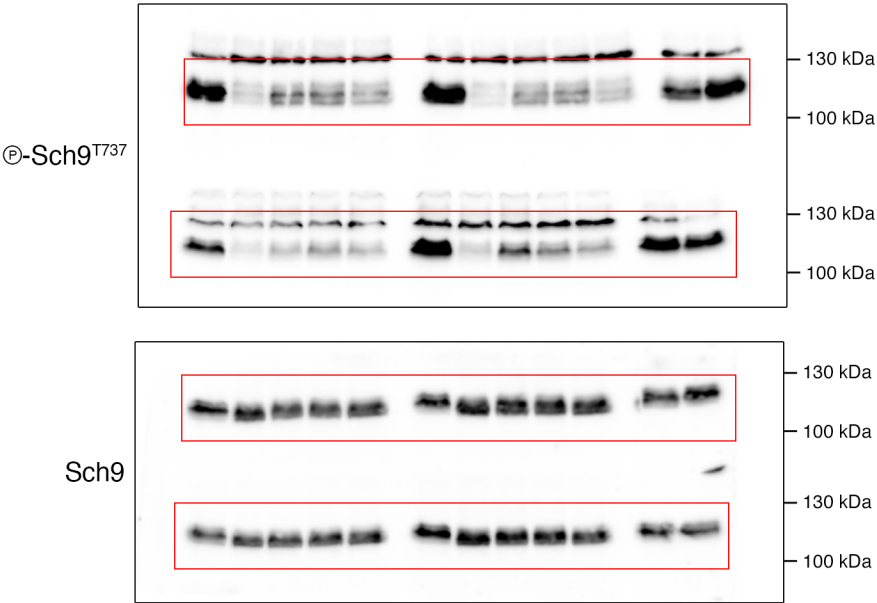

Supplement: Source Data Extended Data Fig. 2 — Uncropped blots for Extended Data Fig. 2. [file 41556_2023_1198_MOESM15_ESM.pdf]

**Uncropped blots for Extended Data Fig. 8g**

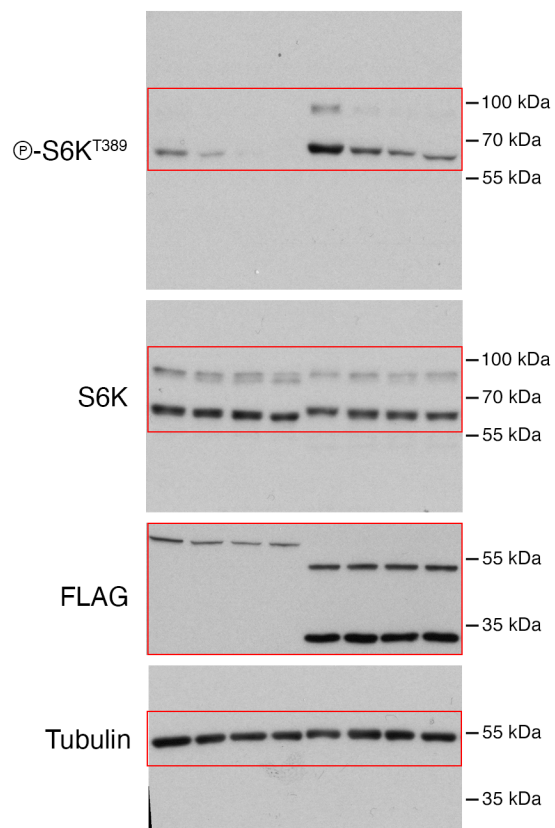

Supplement: Source Data Extended Data Fig. 8 — Uncropped blots for Extended Data Fig. 8. [file 41556_2023_1198_MOESM21_ESM.pdf]
