# Supplementary material for: Malonyl-CoA is a conserved endogenous ATP-competitive mTORC1 inhibitor
Source: Nat Cell Biol. 2023 Aug 10;25(9):1303–18. doi: 10.1038/s41556-023-01198-6 (PMC10495264; doi:10.1038/s41556-023-01198-6)

Uncropped blots for Extended Data Fig. 5a

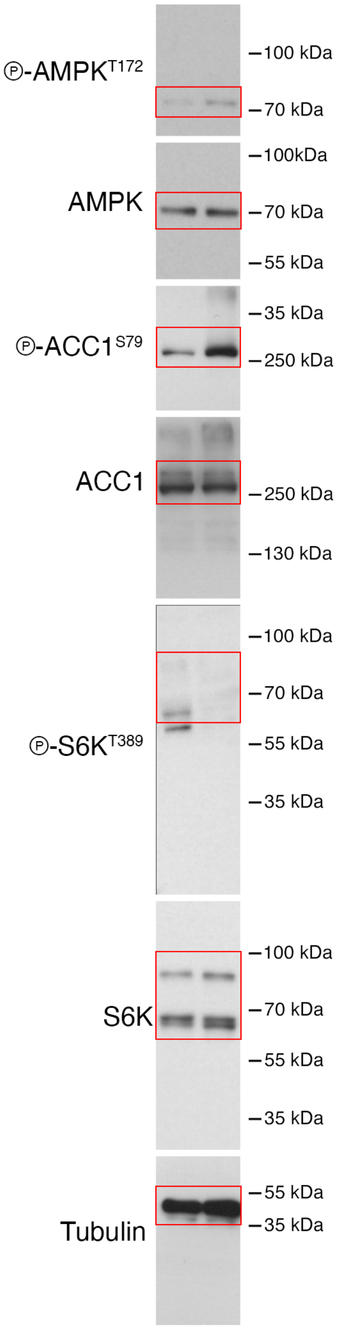

Uncropped blots for Extended Data Fig. 5d

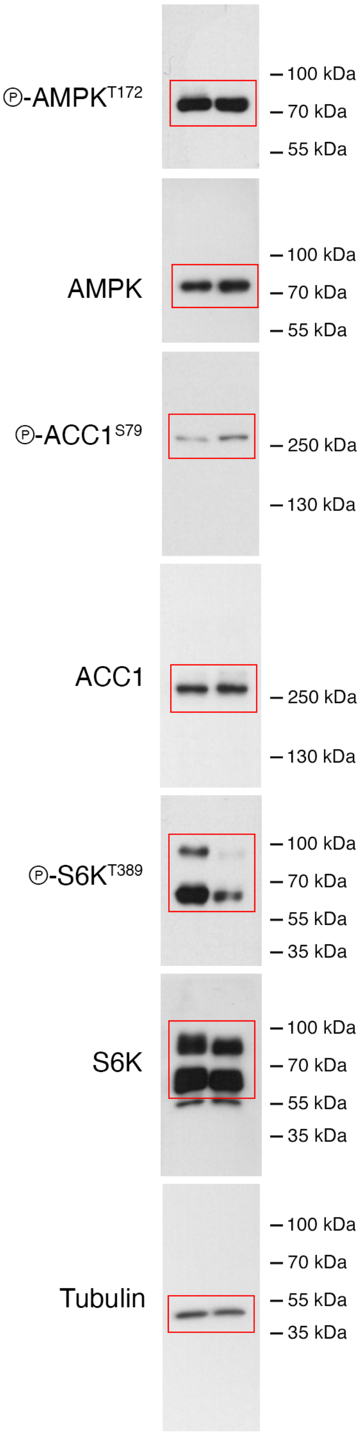

Uncropped blots for Extended Data Fig. 5g

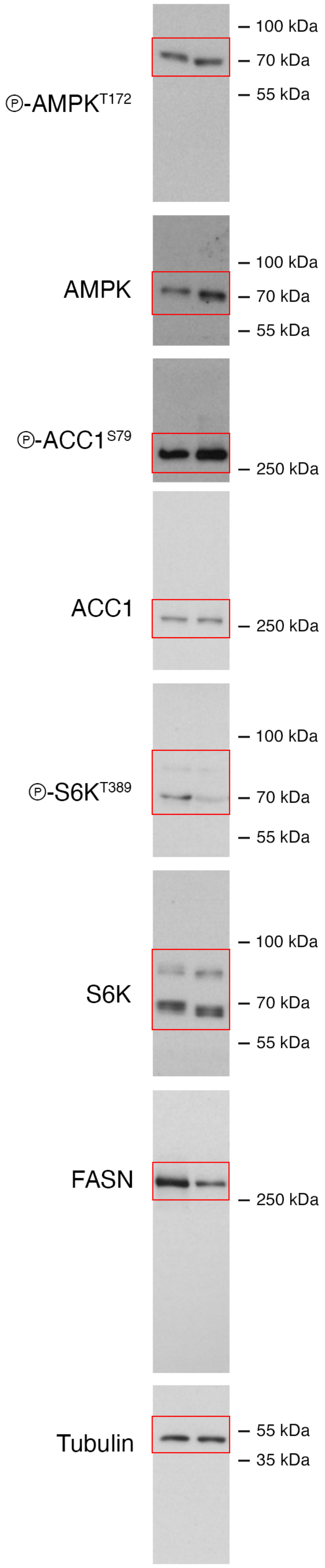

Uncropped blots for Extended Data Fig. 5j

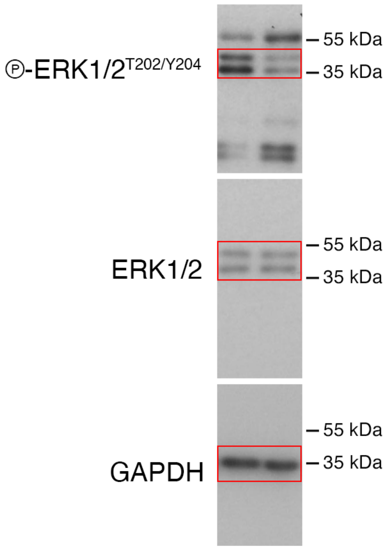

Uncropped blots for Extended Data Fig. 5l

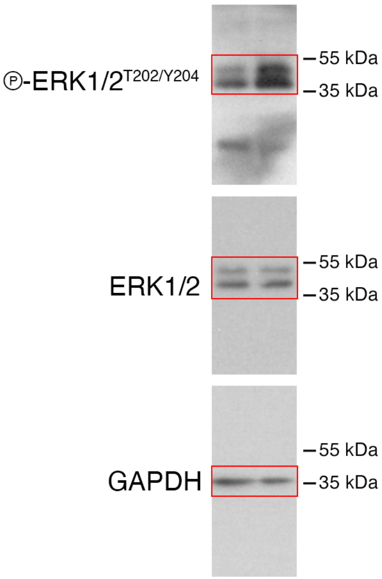

Uncropped blots for Extended Data Fig. 5n

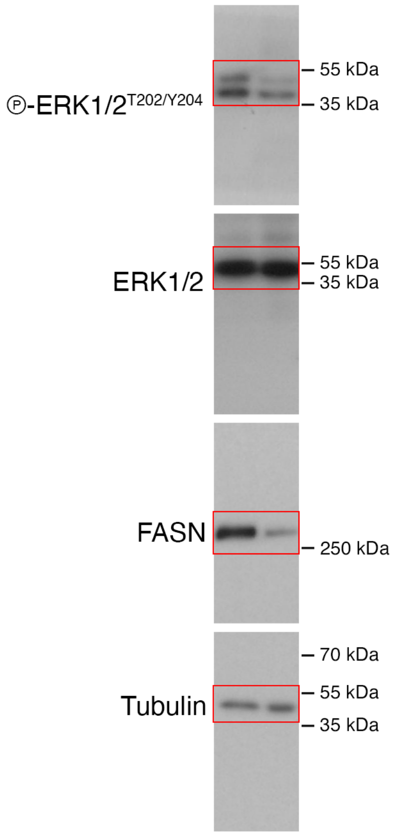

Uncropped blots for Extended Data Fig. 5p

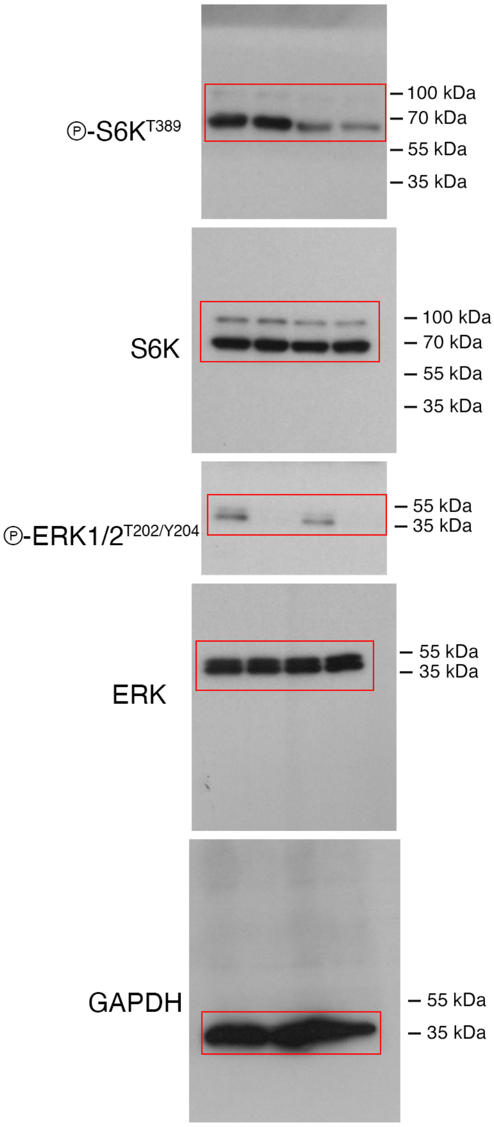

Uncropped blots for Extended Data Fig. 5q

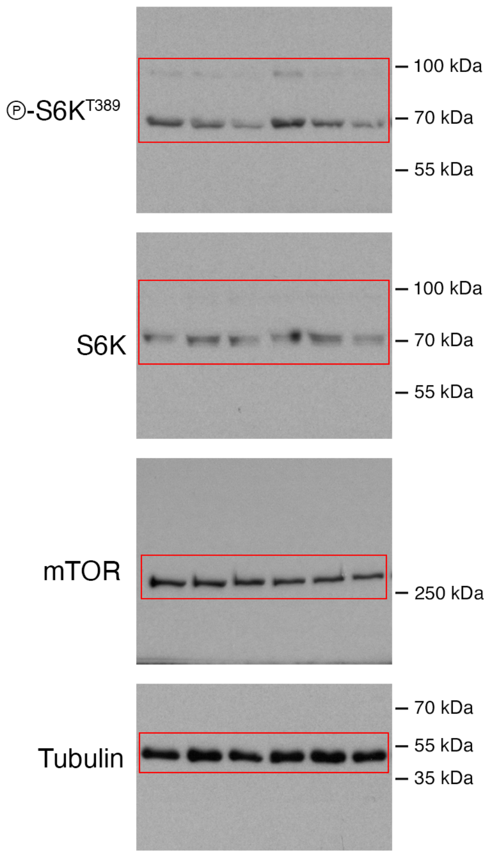

Uncropped blots for Extended Data Fig. 5s

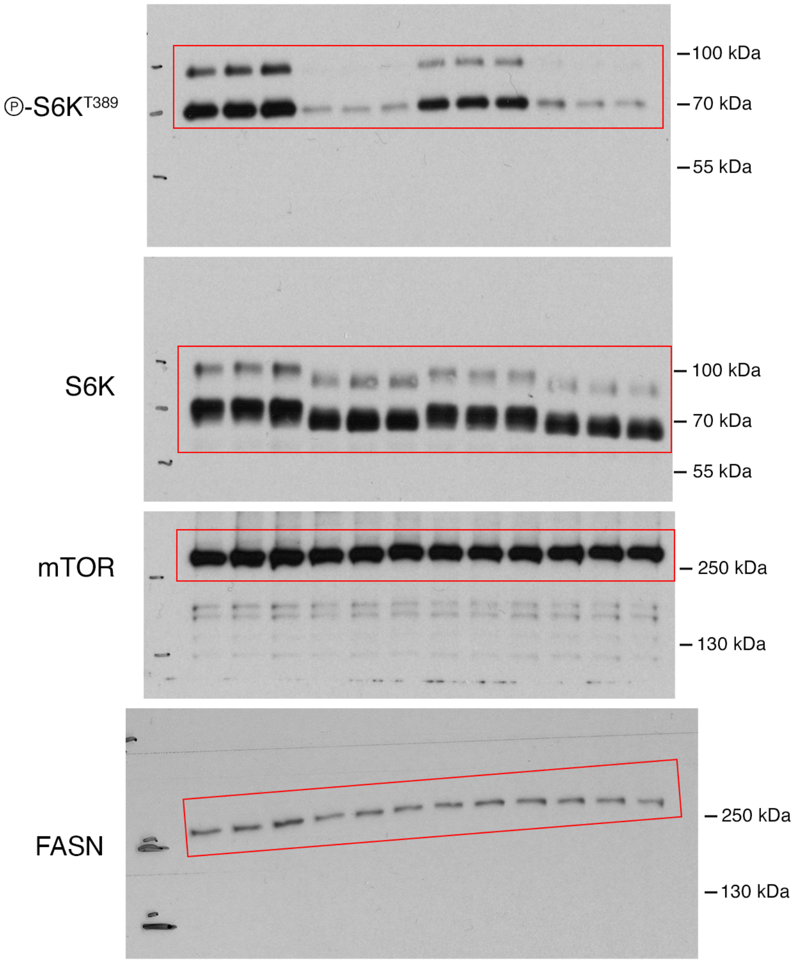

Supplement: Source Data Extended Data Fig. 5 — Uncropped blots for Extended Data Fig. 5. [file 41556_2023_1198_MOESM18_ESM.pdf]
