# Supplementary material for: Malonyl-CoA is a conserved endogenous ATP-competitive mTORC1 inhibitor
Source: Nat Cell Biol. 2023 Aug 10;25(9):1303–18. doi: 10.1038/s41556-023-01198-6 (PMC10495264; doi:10.1038/s41556-023-01198-6)

Uncropped blots for Extended Data Fig. 10b

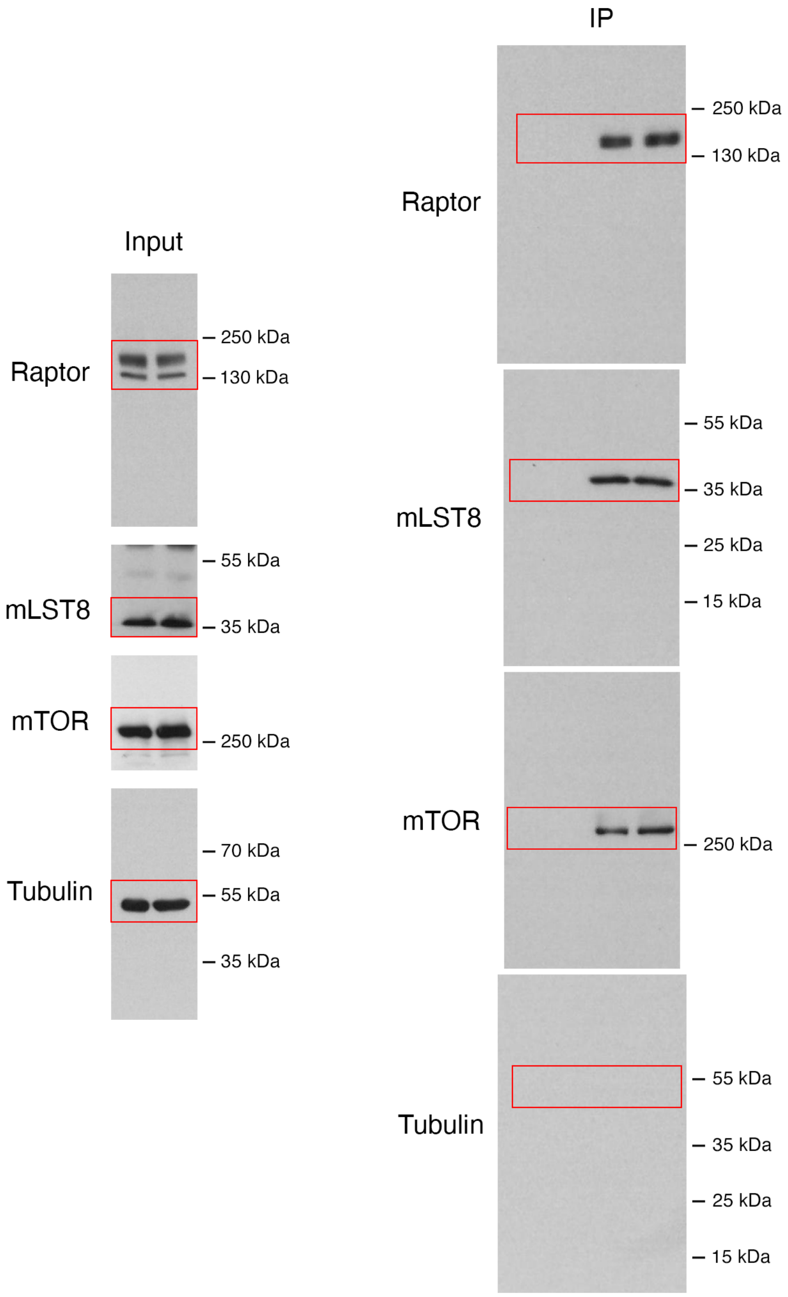

Uncropped blots for Extended Data Fig. 10c

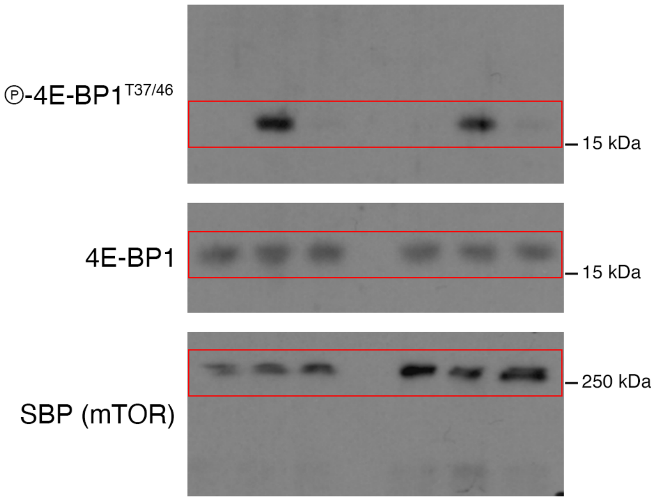

Uncropped blots for Extended Data Fig. 10d

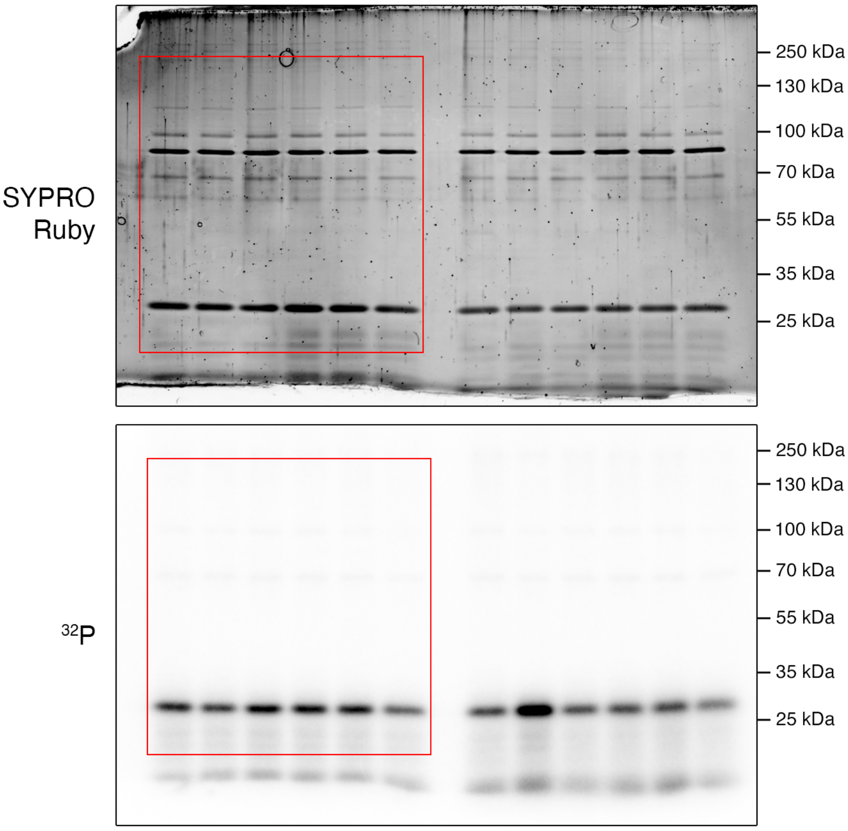

Uncropped blots for Extended Data Fig. 10f

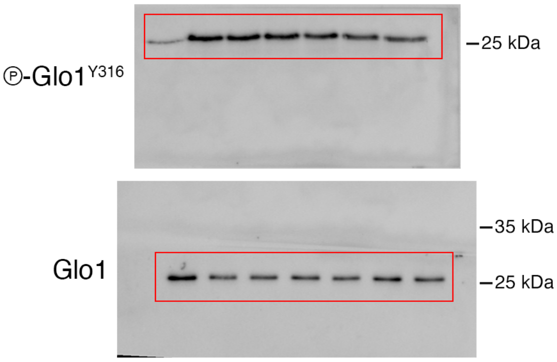

Uncropped blots for Extended Data Fig. 10h

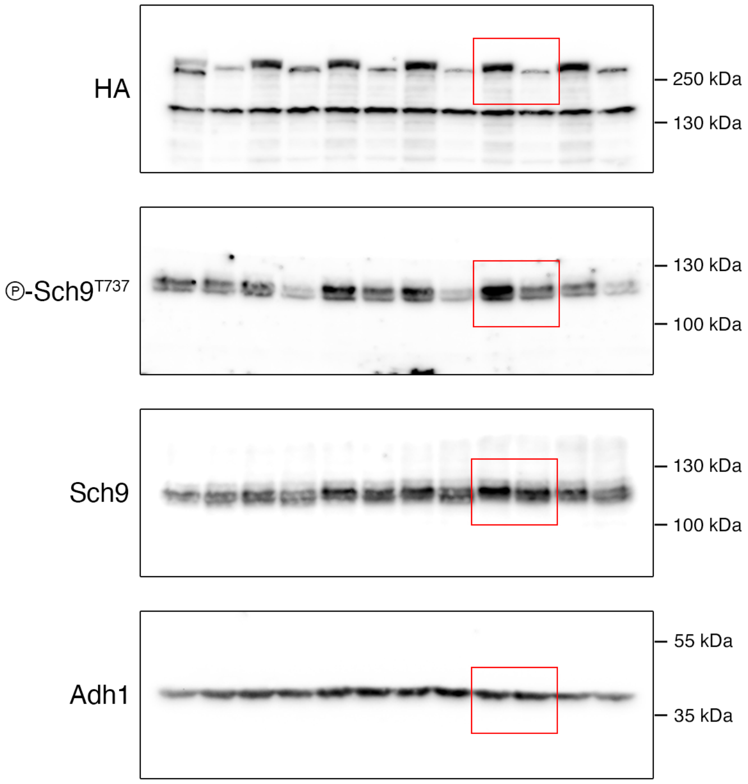

Uncropped blots for Extended Data Fig. 10j

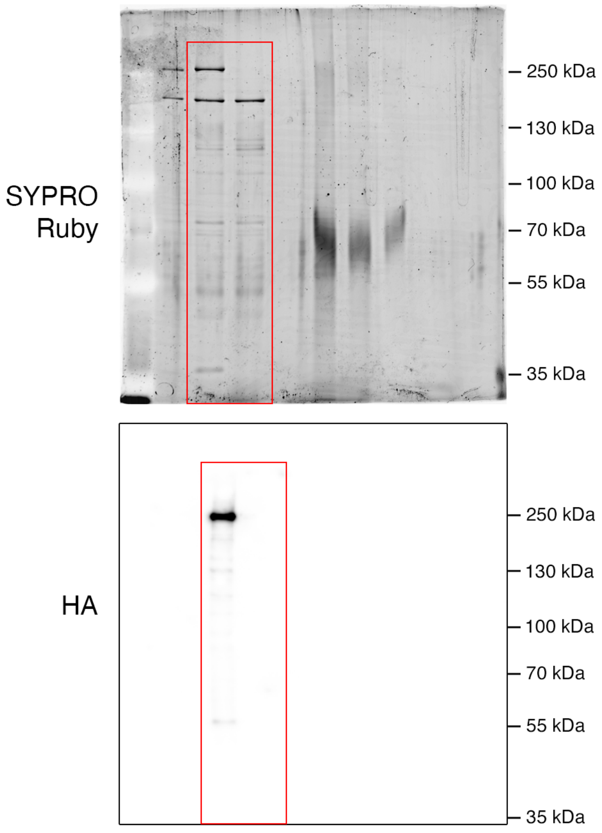

Uncropped blots for Extended Data Fig. 10k

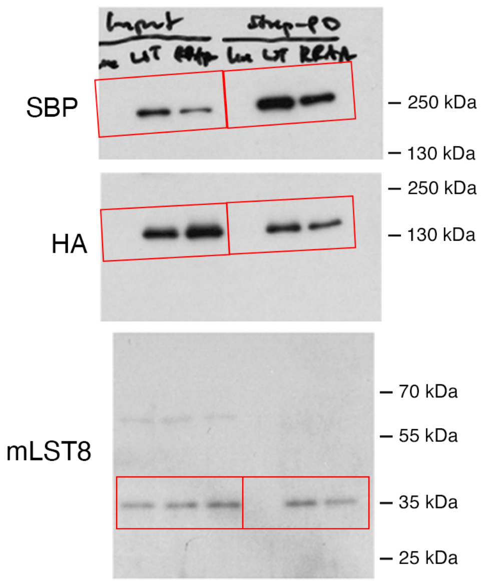

Uncropped blots for Extended Data Fig. 10l

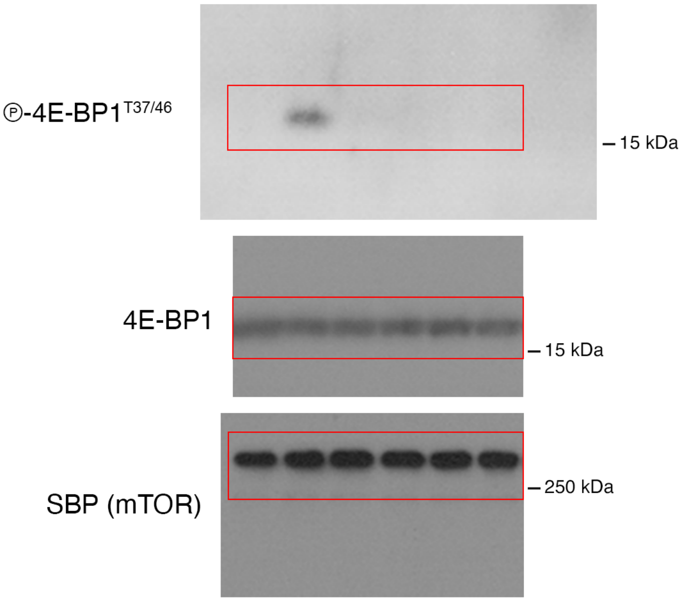

Supplement: Source Data Extended Data Fig. 10 — Uncropped blots for Extended Data Fig. 10. [file 41556_2023_1198_MOESM22_ESM.pdf]
